# Efficient Red Electroluminescent Copper Complexes with Fluorination-Balanced Dual Emission

**DOI:** 10.34133/research.1088

**Published:** 2026-01-14

**Authors:** Xinjing Lou, Gang Chen, Chunyu Liu, Jing Zhang, Jiexu He, Jixiu Niu, Chunbo Duan, Chunmiao Han, Andrey A. Karasik, Hui Xu

**Affiliations:** ^1^ Key Laboratory of Functional Inorganic Material Chemistry (Ministry of Education), School of Chemistry and Material Science, Heilongjiang University, Harbin 150080, P. R. China.; ^2^ Arbuzov Institute of Organic and Physical Chemistry, FRC Kazan Scientific Center, Russian Academy of Sciences, Kazan 420088, Russian Federation.

## Abstract

Copper complexes hold a promise for electroluminescent applications, owing to their dual emissive feature based on the moderate spin-orbital coupling effect of Cu^+^ ion for controllable singlet–triplet conversion. However, efficient red dual emission from copper complexes remains an important challenge, because emission wavelengths and thermally activated delayed fluorescence (TADF)/phosphorescence (PH) ratios are simultaneously correlated to electronic effects. Herein, fluorine atoms with suitable electron-withdrawing inductive effect were introduced into a typical tridentate phosphine ligand coordinated CuI skeleton, namely, TTPPCuI, to reduce the lowest unoccupied molecular orbital (LUMO) energy levels, giving rise to narrowed energy gaps between the highest occupied molecular orbital and LUMO, corresponding to emission wavelengths red shifted from 574 to 603 nm. Fluorine atoms simultaneously enhance metal-ligand charge transfer, therefore adjusting positive and reverse intersystem crossing for dual emission balance, leading to TADF/PH ratios changing from 56/44 over 75/25 to 83/17. The devices based on these fluorinated CuI complexes realized efficient red electroluminescence with the maximum wavelength and external quantum efficiency beyond 600 nm and 20%, respectively. These results demonstrate that, based on electronic effects from functional groups, ligand engineering is a feasible way for comprehensively manipulating excited-state characteristics of dual-emissive copper complexes.

## Introduction

**Fig. 1.**
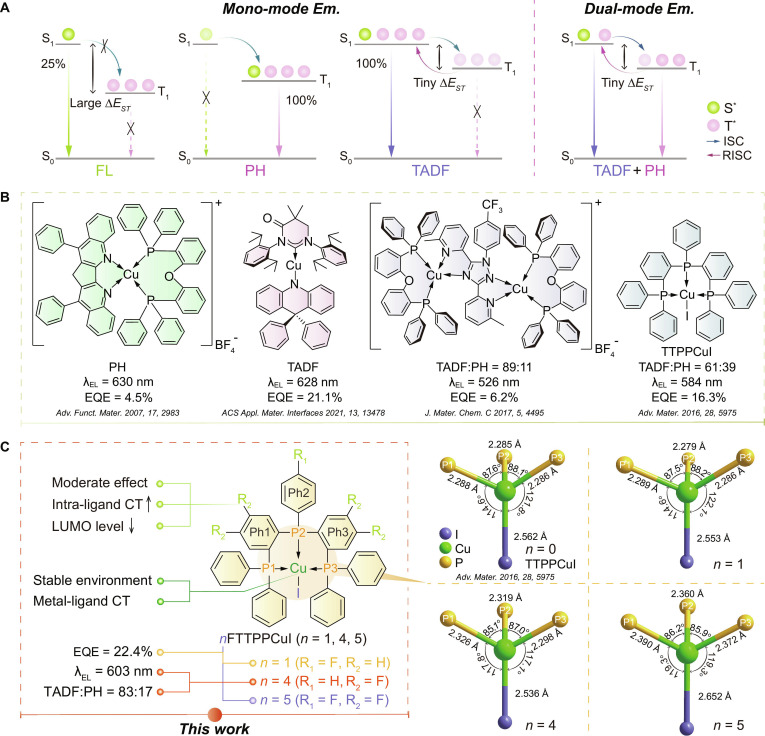
Luminescence mechanism, design strategy, and molecular structures of copper complexes. (A) Electroluminescent (EL) mechanisms with various exciton harvesting processes. FL, PH, TADF, ISC, RISC, and Δ*E*_ST_ are the fluorescence, phosphorescence, thermally activated delayed fluorescence, intersystem crossing, reverse intersystem crossing, and singlet–triplet splitting energy, respectively. Green and pink balls denote singlet and triplet excitons, respectively. S_0_, S_1_, and T_1_ states refer to the ground state, the first singlet, and triplet excited states, respectively. (B) Copper complexes featuring different TADF:PH ratios. λ is peak wavelength. EQE is external quantum efficiency. (C) Molecular design of *n*FTTPPCuI (*n* = 1, 4, and 5), and TTPPCuI (*n* = 0). Chemical structures of *n*FTTPPCuI (left), and their P–Cu–I core structures (right) are highlighted separately. Corresponding bond lengths and angles are marked. CT and SOC refer to charge transfer and spin-orbit coupling, respectively. LUMO is the lowest unoccupied molecular orbital.

Organic light-emitting diodes (OLEDs) are widely applied in modern display and lighting technologies due to their self-luminescence, wide color gamut, low power consumption, and flexible processability [1–7]. They have also demonstrated considerable potential in wearable devices [8], flexible electronics [9,10], and emerging optoelectronic applications [11]. Enhancing exciton utilization efficiency (EUE) is a key strategy for improving OLED performance [12]. According to spin statistics [13], singlet and triplet excitons are generated in a 1:3 ratio during electrical excitation. In conventional fluorescence (FL) [14], phosphorescence (PH) [15], and thermally activated delayed fluorescence (TADF) [16], either the first singlet (S_1_) or triplet (T_1_) excited state contributes to emission through a single radiative transition process (Fig. 1A). These mono-emissive pathways tend to cause exciton accumulation, which intensifies singlet–triplet and triplet–triplet annihilation (STA [17] and TTA [18], respectively). To address these limitations, dual-emissive materials that combine TADF and PH have emerged as a promising solution (Fig. 1A). These materials offer direct radiative channels for both singlet and triplet excitons, reducing energy loss and facilitating efficient exciton management by accelerating emission and suppressing quenching processes [19–27].

Among dual-emissive systems, copper complexes stand out due to their moderate spin-orbit coupling (SOC) and tunable excited-state properties [20,28], including metal-to-ligand charge transfer (MLCT), iodine-to-ligand charge transfer (ILCT), and intraligand charge transfer (LCT) states [29,30]. Moderate SOC allows both reverse intersystem crossing (RISC) from the T_1_ to the S_1_ and radiative decay from the T_1_ to the ground state (S_0_), enabling dual emission without reliance on heavy metals [31–33]. Fine-tuning the electronic effects, steric hindrance, and charge distribution of ligands enables control over excited-state energy levels and transition pathways, allowing a balanced contribution from TADF and PH. However, TADF and PH are inherently competitive. Enhancing MLCT can improve triplet localization and promote PH [20], but simultaneously narrows the singlet–triplet splitting energy (Δ*E*_ST_), which promotes RISC and enhances TADF [34]. Moreover, MLCT modulation alters the structure of excited-state energy levels and shifts the emission wavelength, making it difficult to simultaneously optimize both dual-emissive behavior and emission color [35].

Multinuclear copper cluster complexes, owing to their diverse channel emission, hold promise for applications in anti-counterfeiting, information encryption, and sensing, yet their structural complexity and challenging synthesis limit practical application [36–38]. By contrast, simple mononuclear dual-emissive copper complexes have been achieved in the blue and green regions [39–43], whereas achieving balanced dual emission in the red region remains challenging. Long-wavelength emission typically requires enhanced LCT or MLCT character to reduce the energy gap and red-shifted emission, which tends to suppress PH and favor TADF [44,45]. Additionally, smaller bandgaps are more prone to exciton thermal quenching and nonradiative decay, limiting emission efficiency [46–49]. Therefore, the development of efficient red dual-emissive copper complexes necessitates the precise optimization of intramolecular interactions. This, in turn, necessitates the development of ligand engineering strategies capable of modulating ligand-centered interactions.

For red-emitting copper complexes, most approaches rely on extending the π-conjugated ligand framework and enhancing molecular rigidity to achieve red-shifted emission and suppress nonradiative decay [50]. The first reported red-emitting mononuclear copper complex [Cu(mdpbq)(DPEphos)] (BF_4_) primarily exhibited PH but suffered from limited exciton harvesting efficiency, yielding an external quantum efficiency (EQE) lower than 5% [51]. A subsequent development introduced a carbene–metal–amide-type copper complex, MAC*–Cu–DPAC, incorporating a strongly conjugated donor, 9,9-diphenyl-9,10-dihydroacridine (DPAC). This complex achieved red emission at ~630 nm with an EQE of ~20% [44]. However, the dominant ligand-centered excited state resulted in an almost zero Δ*E*_ST_ and RISC, leading to emission exclusively through TADF. As a result, PH was nearly completely suppressed, undermining the multifunctionality of dual-emissive behavior (Fig. 1B).

By contrast, inductive effects offer greater potential for the development of red dual-emissive materials. Electron-withdrawing groups, such as trifluoromethyl, can effectively modulate charge distribution and MLCT characteristics without markedly extending the π-conjugated system. This enables precise control over excited-state energy levels and emission mechanisms [52]. Although the resulting EQE remained relatively low (<10%) and the emission shift was modest (from 523 to 526 nm), the PH contribution increased to 11%, resulting in a more balanced emission profile (Fig. 1B). Compared with strategies based on π-conjugation extension, inductive effects provide broader advantages in tuning emission mechanisms, color, and excited-state dynamics, offering a promising design framework for high-performance red dual-emissive copper complexes.

Building on our previously reported yellow dual-emissive copper complex, TTPPCuI [12], this study introduces fluorine atoms with extremely small steric hindrance and suitable electron-withdrawing inductive effects into the tridentate triphosphine ligand (TTPP) [53,54]. Three fluorinated copper complexes, *n*FTTPPCuI (*n* = 1, 4, and 5), were synthesized (Fig. 1C, Figs. S1 to S22, and Scheme S1). In mononuclear copper complexes, the highest occupied molecular orbital (HOMO) is typically localized on the copper center, while the lowest unoccupied molecular orbital (LUMO) resides on the ligand framework [55–57]. Fluorine incorporation effectively lowers the LUMO energy, reducing the HOMO–LUMO energy gap and inducing red-shifted emission [58–60]. Moreover, because fluorine lacks π-conjugation, it does not substantially alter the nature of the emissive states, helping to maintain a balanced contribution from TADF and PH. Thus, 4FTTPPCuI represents the first long-wavelength copper complex to exhibit dual emission composed of ~80% TADF/~20% PH. FTTPPCuI exhibits a more balanced emission profile (~50% TADF/~50% PH), achieving an EQE of ~22% and a luminance of ~17,000 cd cm^−2^. The emission wavelengths of the *n*FTTPPCuI series can be finely tuned within a narrow range of 574 to 603 nm, confirming the effectiveness of ligand engineering strategies in simultaneously regulating emission color and dual-emissive composition. These findings provide a viable pathway for the design of high-performance, long-wavelength dual-emissive copper complexes.

## Results

### Coordination structures and theoretical simulation

The tridentate phosphine ligands containing fluorine atoms form highly stable and rigid copper complexes, as confirmed by single-crystal structural analysis (Figs. S23, S35, and S36) [12,43,61–64]. In the *n*FTTPPCuI series, the overall tetrahedral coordination framework of the P–Cu–I core remains largely unchanged (Fig. 1C). However, stepwise fluorine substitution systematically modulates the local coordination environment and molecular geometry (Fig. S24 and Tables S1 to S4). In FTTPPCuI, the introduction of a single fluorine atom at the R_1_ position of Ph2 (connected to the P2 atom) slightly reduces the electron-donating ability of the ligand, resulting in a marginal shortening of the Cu–I bond. In contrast, 4FTTPPCuI, with 4 fluorine atoms positioned at R_2_ sites on Ph1 and Ph3, exhibits a stronger electron-withdrawing effect. This leads to a marked elongation of the P–Cu bond, while the Cu–I bond continues to shorten, suggesting an enhancement of metal–iodine interactions. Further fluorination in 5FTTPPCuI, through the addition of a fifth fluorine atom, causes excessive electron withdrawal from the metal center. This markedly alters the metal–ligand interaction mode. Both the P–Cu and C–P bonds are further elongated, increasing steric hindrance around the Cu^+^ ion. As a result, the Cu–I bond is extended to 2.652 Å. Despite these changes, variation in bond angles is minimized, and the coordination geometry of 5FTTPPCuI closely approaches an ideal tetrahedral configuration. These observations confirm that fluorine substitution leads to distinct variations in coordination structures. Notably, no obvious intermolecular interactions are observed in 5FTTPPCuI due to its highly symmetrical ligand structure (Fig. S27). In contrast, the more distorted FTTPPCuI and 4FTTPPCuI exhibit weak hydrogen bonding interactions in the crystal lattice (Figs. S25 and S26).

Thermogravimetric analysis and differential scanning calorimetry reveal excellent thermal stability for all 3 complexes. The melting temperatures (*T*_m_) exceed 310 °C, and decomposition temperatures (*T*_d_) are higher than 340 °C. This high thermal resilience makes these complexes suitable candidates for vacuum-evaporated device fabrication (Fig. S28 and Table S8) [65].

**Fig. 2.**
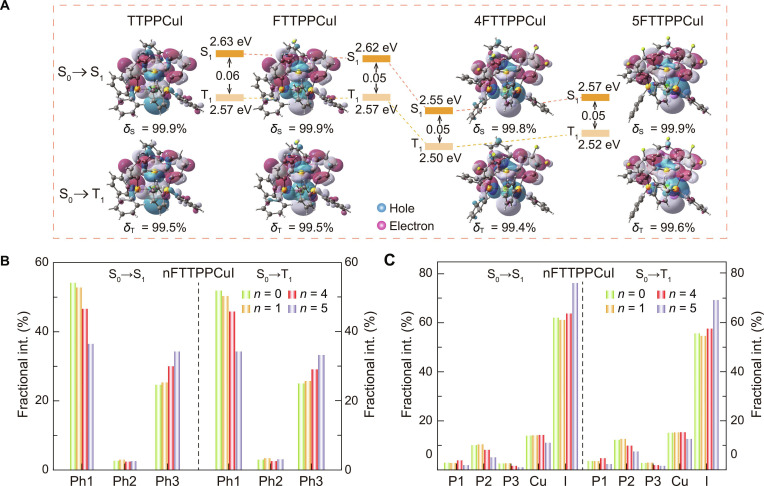
Simulated electronic properties of *n*FTTPPCuI (*n* = 0, 1, 4, and 5) complexes. (A) Frontier molecular orbital (FMO) contours and key parameters of the S_0_→S_1_ and S_0_→T_1_ excitations simulated with natural transition orbital (NTO) analysis. The contours of “holes” and “electrons” are distinguished with blue and pink colors, respectively. *δ* and E represent the contribution weight and excited-state energy level, respectively. Subscripts “S” and “T” represent “singlet” and “triplet”, respectively. (B) Contribution percentage of 3 phenyl rings (Ph1, Ph2, and Ph3) in *n*FTTPPCuI complexes to the electron during the S_0_→S_1_ and S_0_→T_1_ transitions. (C) Contribution percentage of representative atoms (P1, P2, P3, Cu, and I) to the hole during the S_0_→S_1_ and S_0_→T_1_ transitions.

The S_0_ and excited singlet and triplet states were investigated using density functional theory (DFT) and time-dependent DFT (TDDFT) methods (Fig. 2A). The HOMOs of the 4 complexes are primarily localized on the CuI core and partially delocalized onto the nFTTPP segment, whereas the LUMOs are mainly distributed over the *o*-phenylene groups and the central phosphorus bridge of the nFTTPP ligands (Fig. S29). This spatial separation between the HOMOs and the LUMOs indicates pronounced charge-transfer interactions between the ligands and the CuI core. This separation effectively reduces the Δ*E*_ST_, thereby creating favorable conditions for RISC from the T_1_ to the S_1_ excited state [66–68]. With increasing fluorine substitution, the LUMO energy levels are progressively lowered from −1.5 eV in TTPPCuI to −2.1 eV in 5FTTPPCuI. Concurrently, the HOMO–LUMO energy gap decreases from 3.3 eV (TTPPCuI) to 3.1 eV (5FTTPPCuI), consistent with the observed red-shifted emissions. This trend arises from the electron-withdrawing inductive effect of fluorine atoms, which weakens the MLCT and enhances the LCT component (Table S5) [69]. Experimental cyclic voltammetry (CV) measurements show HOMO and LUMO energy levels of approximately 5.5 and 2.9 eV, respectively, for the *n*FTTPPCuI complexes—well aligned with typical host and carrier-transporting materials (Fig. S32 and Table S8).

Natural transition orbital (NTO) analysis of the S_0_→S_1_ and the S_0_→T_1_ transitions reveals that the “holes” and “electrons” orbitals in both excited states are mainly localized on the CuI core and nFTTPP ligands, respectively, indicating that these excitations primarily correspond to HOMO→LUMO transitions with mixed M/ILCT characteristics. Fluorine substitution redistributes electron density within the ligands and enhances LCT characteristics (Fig. S30 and Tables S6 and S7).

In the S_0_→S_1_ transition of FTTPPCuI, the fluorine atom at the R_1_ position of Ph3 induces only a weak inductive effect, resulting in marginal increases in the electron contributions from P2 and Ph2 by 0.25% and 0.38%, respectively (Fig. 2B and Fig. S31). In contrast, 4FTTPPCuI features fluorine atoms at the R_2_ positions of Ph1 and Ph3, substantially enhancing the inductive effect. This modification drives electron density from the phenyl rings toward the central phosphorus atoms, increasing the electron contributions from P1 and P3 by 0.41% and 0.54%, respectively, while reducing that from Ph1 by 7.52%. The resulting excited-state reorganization strengthens the LCT character, lowers the LUMO energy level, narrows the HOMO–LUMO gap, and contributes to a more pronounced red shift [70]. In 5FTTPPCuI, an additional fluorine atom at the R_2_ position of Ph2 works synergistically with the existing fluorine at the R_1_ position. This induces a notable increase in the electron contribution from P2 by 5.57% and a modest increase from Ph2. Simultaneously, the contributions from P1 and Ph1 decrease markedly, resulting in strongly localized excited-state orbitals in the P2–Ph2 region. This increased asymmetry reduces charge separation, elevates the LUMO energy, and limits further red shifting. Moreover, the greater contribution of iodine to the hole orbitals in 5FTTPPCuI enhances ILCT character and diminishes MLCT, thereby increasing SOC and improving PH efficiency (Fig. 2C and Fig. S31) [71,72].

All 3 fluorinated complexes exhibit small Δ*E*_ST_ of approximately 0.05 eV (Table S8), which facilitate efficient intersystem crossing (ISC) and RISC between the S_1_ and the T_1_ states [73]. This enables dual emission and enhances EUE. Thus, fluorine-induced electron density redistribution not only alters excited-state configurations but also finely tunes the LUMO energy and overall bandgap, providing a mechanism for precise control of both emission wavelength and dual-emissive ratio.

### Photophysical properties

**Fig. 3.**
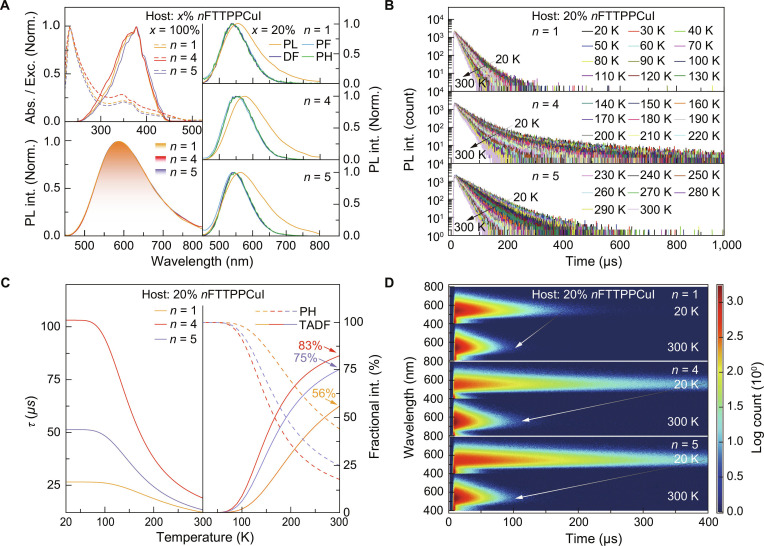
Photophysical properties of *n*FTTPPCuI (*n* = 1, 4, and 5) complexes. (A) Electronic absorption spectra (dashed lines, upper left), excitation spectra (solid lines, upper left), and photoluminescence (PL) spectra (bottom left) of neat *n*FTTPPCuI films. PL, prompt fluorescence (PF), delayed fluorescence (DF), and PH spectra of Host:20% *n*FTTPPCuI-doped films (right). PF, DF, and PH spectra were measured with time-resolved technology, which recorded in the ranges of <1 μs, 1 to 200 μs, and >200 μs, respectively. (B) Temperature-dependent time decays for Host:20% *n*FTTPPCuI-doped films in the range of 20 to 300 K with an interval of 10 K. For *n* = 1, 4, and 5, the host materials are *m*CP, CBP, and *m*CP, respectively. The variation tendencies are marked with arrows. (C) Lifetime vs. temperature relationship (left) and temperature-dependent TADF:PH ratios (right) for doped films simulated with time decay data from (B). TADF fractional intensities at 300 K are marked. (D) Temperature-dependent time-resolved emission spectra (TRES) of doped films at 20 and 300 K. The variation tendencies are marked with arrows.

The electronic absorption spectra of neat *n*FTTPPCuI films exhibit 4 absorption bands within the 200 to 500 nm range (Fig. 3A and Table S8). A strong absorption peak appears at ~240 nm, and a weaker band centered around 350 nm corresponds to π→π* and n→π* transitions, respectively. The absorption features near 370 nm are attributed to LCT, while the extended absorption tails from 400 to 500 nm are assigned to M/ILCT, similar to TTPPCuI (Fig. S33) [12]. Excitation spectra indicate multiple transition types—including LCT, MLCT, and ILCT—whose relative intensities vary among the complexes (Fig. 3A). Notably, 4FTTPPCuI displays the strongest excitation intensity at ~370 nm, indicating an enhanced LCT character. In contrast, LCT transitions in 5FTTPPCuI are reduced, while the excitation intensity near 400 nm is enhanced, pointing to an increased ILCT contribution. These trends are consistent with the excited-state compositions revealed by NTO analysis.

Compared with TTPPCuI, the photoluminescence (PL) spectra of neat *n*FTTPPCuI films show red-shifted emission by ~67 nm, with emission maxima near 590 nm at room temperature, and exhibit excellent photostability (Fig. 3A and Figs. S33 and S37) [25]. This red shift is attributed to fluorine-induced lowering of the LUMO energy, narrowing the HOMO–LUMO gap [60]. In solution, the PL spectra exhibit pronounced blue shifts in the emission wavelength, transitioning from approximately 590 nm in neat films to 410 nm. This notable blue shift can be attributed to a marked reduction in intermolecular interactions within the solution, which contrasts with the stronger interactions present in the films (Fig. S38). In doped films, PL emission peaks are observed at 555 nm for 1,3-bis(carbazol-9-yl)benzene (*m*CP):20% FTTPPCuI, 575 nm for 4,4′-bis(9H-carbazol-9-yl)biphenyl (CBP):20% 4FTTPPCuI, and 563 nm for *m*CP:20% 5FTTPPCuI (Fig. 3A and Fig. S44). These emissions show negligible temperature dependence, indicating a dual-emissive origin (Fig. S46) [74]. Furthermore, the prompt fluorescence (PF), delayed fluorescence (DF), and PH spectra of the doped films are nearly identical (Fig. 3A), suggesting similar electronic distributions and emission pathways for the S_1_ and the T_1_ states. From the PH and FL spectra of the neat films, the Δ*E*_ST_ for the *n*FTTPPCuI complexes are estimated to be ~0.04 eV, in agreement with theoretical values (Fig. S43). This small Δ*E*_ST_ facilitates efficient ISC and RISC, supporting the occurrence of dual emission.

Time decay curves of doped *n*FTTPPCuI films are bi-exponential, reflecting microsecond-scale lifetimes (Fig. 3B). At room temperature, these films exhibit shorter lifetimes (~20 μs) compared to their neat films (Figs. S34 and S45). In solution, lifetimes are further reduced due to increased nonradiative relaxation (Fig. S39). Furthermore, all 3 complexes exhibit high RISC rate constants (*k*_RISC_), up to 10^5^ s^−1^, with quantum efficiencies of RISC (*ϕ*_RISC_) reaching as high as 90% (Fig. S45 and Table S9). These findings suggest a transition from the T_1_ to the S_1_ excited state, thereby confirming the TADF behavior. Therefore, at 20 K, the excited state is predominantly confined to the T_1_ state, resulting in PH emission characterized by a relatively extended lifetime. As the temperature rises to 300 K, the excited state undergoes a thermally activated RISC process, transitioning from the long-lived T_1_ state to the short-lived S_1_ state, therefore decreasing the emission lifetime (Fig. 3B) [43,62,75].

Analysis of the dual-emissive components at 300 K shows that FTTPPCuI displays a balanced contribution from 56% TADF/44% PH (Fig. 3C). In contrast, 4FTTPPCuI exhibits a markedly enhanced TADF contribution (83%) due to its stronger LCT characteristics. In comparison, 5FTTPPCuI, owing to its more pronounced ILCT characteristics, shows an increased PH contribution, decreasing the TADF ratio to 75%. Consistent with these observations, the TADF/PH ratios measured in neat films exhibit the same trend as those in doped films (Figs. S40 and S41). This consistency confirms that the variations in TADF/PH ratios originate from the intrinsic excited-state compositions of the complexes rather than host–matrix effects. It is noteworthy that 4FTTPPCuI exhibits a relatively low *k*_RISC_ and the ratio of the singlet radiative to nonradiative rate constant ($k_{r}^{S}/k_{\mathrm{nr}}^{S}$), resulting in a prolonged decay process and longer lifetime (Table S9). In comparison, 5FTTPPCuI shows a slight increase in the $k_{r}^{S}/k_{\mathrm{nr}}^{S}$ ratio, resulting in a modest reduction in its lifetime [20,65,72,76,77]. Additionally, temperature-dependent time-resolved emission spectra (TRES) show that, compared with neat films, the lifetimes in doped films are slightly prolonged due to suppression of nonradiative quenching by the host matrix (Fig. 3D and Figs. S42 and S47).

### Electroluminescence properties

**Fig. 4.**
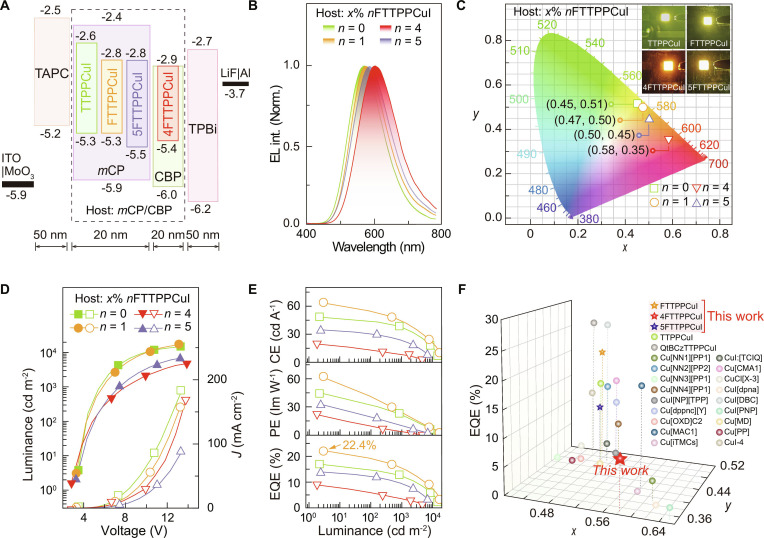
EL performance of *n*FTTPPCuI (*n* = 0, 1, 4, and 5)-based OLEDs. (A) Energy level diagram of the devices. (B) EL spectra at the respective optimal doping concentrations. (C) Device photos (inset) at 5 V, and the corresponding chromatic coordinates at CIE 1931 panel. CIE coordinates at the respective optimal doping concentrations are marked. (D) Current density (*J*, open symbols)–luminance (solid symbols)–voltage characteristics of the devices at the optimal doping concentrations (*x*%). (E) Efficiencies vs. luminance relationships of the devices. CE and PE refer to current and power efficiencies, respectively. EQE value of FTTPPCuI at the maximum are marked with an arrow. (F) Milestones of the EQE values and the corresponding chromatic coordinates at CIE achieved by copper complexes with EL wavelengths in the long-wavelength range in devices. The corresponding detailed performances are listed in Table S12. The 4FTTPPCuI complex is highlighted with a red pentagram, representing the first red-emitting copper complex exhibiting dual-emissive behavior.

Vacuum-evaporated light-emitting devices with the configuration ITO|MoO_3_ (6 nm)|TAPC (50 nm)|Host:*x*% *n*FTTPPCuI (*n* = 0, 1, 4, and 5) (20 nm)|TPBi (50 nm)|LiF (1 nm)|Al (100 nm) were fabricated. TAPC (di-[4-(N,N-ditolylamino)phenyl]cyclohexane) and TPBi (1,3,5-tris(N-phenylbenzimidazol-2-yl)benzene) served as the hole- and electron-transporting layers, respectively. *m*CP and CBP were used as the host materials (Fig. 4A and Fig. S51).

At optimized doping levels, the electroluminescent (EL) emission wavelengths were precisely tuned by varying the number of fluorine atoms in the ligand framework—from 572 nm (TTPPCuI) to 574 nm (FTTPPCuI), 584 nm (5FTTPPCuI), and 603 nm (4FTTPPCuI) (Fig. 4B). FTTPPCuI and 5FTTPPCuI both exhibited yellow EL emission, whereas 4FTTPPCuI exhibited a marked red shift (~30 nm), with corresponding Commission Internationale de l’Eclairage (CIE) coordinates of (0.58, 0.35) (Fig. 4C). Fluorine substitution also markedly improved electrical properties, reducing the turn-on voltage from 3.5 to 2.8 V (Figs. S52 to S61 and Tables S10 and S11), indicating enhanced carrier injection and recombination of 4FTTPPCuI [78]. Furthermore, the current–voltage characteristics of these devices were consistent with those of single-carrier transport devices based on *n*FTTPPCuI, confirming the important contribution of Cu(I) dopants to carrier transport (Fig. S62).

FTTPPCuI demonstrated the excellent device performance, achieving a peak EQE of ~22%, a current efficiency (CE) of ~64 cd A^−1^, a power efficiency (PE) of ~63 lm W^−1^, and a maximum luminance of ~17,000 cd m^−2^ (Fig. 4D and E). These values represent a ~6% improvement in EQE over TTPPCuI (Fig. S52) [12]. 5FTTPPCuI achieved an EQE of 14% and a peak luminance of ~7,000 cd m^−2^ (Fig. S55). Notably, 4FTTPPCuI produced red-shifted emission while retaining favorable device performance, with an EQE of 9%, a CE of ~20 cd A^−1^, a PE of ~22 lm W^−1^, and a maximum luminance of ~4,700 cd m^−2^ (Fig. S58). Moreover, 4FTTPPCuI was the first red-emitting copper complex to exhibit dual-emissive behavior and delivered better device performance in the red region with a CIE *x*-coordinate exceeding 0.56 (Fig. 4F), compared with other types of materials, such as perovskite emitters, highlighting its potential for red OLED applications [79,80]. Meanwhile, the PL quantum yield of 4FTTPPCuI (53%) was markedly lower than that of FTTPPCuI (88%) and 5FTTPPCuI (71%), consistent with its lower device EQE (Figs. S48 to S50). This suggests that the reduced efficiency arises from intrinsic emitter rather than device structure, highlighting the key role of emissive properties in device performance.

**Fig. 5.**
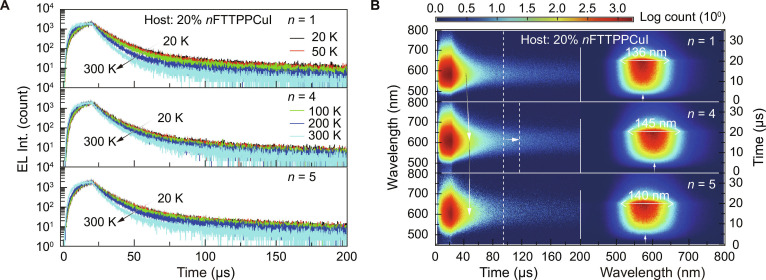
Exciton kinetics in the devices. (A) EL time decays of Host:20% *n*FTTPPCuI (*n* = 1, 4, and 5)-based devices in the temperature range of 20 to 300 K. (B) Contours of time-resolved electroluminescence emission spectra (TREES, left) in the range of 200 μs and sliced contours (right) of the OLEDs at the first 35 μs. Recombination ranges are at the first 20 μs.

The exciton conversion dynamics in EL differ from those in PL. PL primarily excites the S_1_ state, with the T_1_ states generated through ISC, resulting in a limited population of triplet states. By contrast, EL directly forms approximately 75% T_1_ excitons through carrier injection and recombination, serving as a more abundant source of triplets for the TADF process [81]. Although the EL spectra were temperature-independent, the emission lifetimes of *n*FTTPPCuI-based devices decreased by increasing temperature (Fig. 5A and Figs. S63 to S65). Among them, 4FTTPPCuI devices exhibited minimal temperature dependence in time-resolved decay, indicating a more efficient RISC process at low temperatures and a greater contribution from TADF.

Time-resolved electroluminescence emission spectra (TREES) showed that emissions from FTTPPCuI and 5FTTPPCuI devices were more temporally concentrated, whereas 4FTTPPCuI devices exhibited extended tails due to slower radiative decay (Fig. 5B). This behavior was attributed to stronger LCT character in 4FTTPPCuI, which reduced its T_1_ energy level (2.50 eV) compared to FTTPPCuI (2.57 eV) and moved it further away from the triplet level of the *m*CP host (~2.9 eV) [82]. This alignment prolonged exciton lifetimes and extended the TREES tail. Sliced TREES further revealed that exciton confinement and recombination in 4FTTPPCuI were delayed by ~1 μs compared to FTTPPCuI. Unlike in PL, excited-state formation in the EL process is driven by charge injection, which further enhances charge-transfer characteristics [83]. FTTPPCuI-based devices exhibit lower TADF contributions and narrower emission bandwidths (~136 nm), while 4FTTPPCuI and 5FTTPPCuI devices show broader emission bands (~145 and ~140 nm, respectively) due to higher TADF components. These results underscore the importance of excited-state engineering in controlling EL behavior.

## Discussion

“Ligand fluorination” strategy is adopted to rationally optimize the excited-state characteristics of CuI complexes for realizing efficient red dual emission. The feature of fluorine atom with only electron-withdrawing inductive effect is utilized to continuously adjust the emission wavelengths of *n*FTTPPCuI complexes in a range from 574 to 603 nm by changing the substitution position and number of fluorine atoms. Simultaneously, the modulated MLCT by fluorination makes TADF/PH ratios varied from 56/44 to 83/17. FTTPPCuI with a balanced TADF/PH ratio of ~50/50 endowed its device with the EQE up to ~22%, while 4FTTPPCuI-based devices achieved the desired red emission purity with CIE*_x_* beyond 0.56, demonstrating the first red dual-emissive OLED. This work demonstrates not only the feasibility of controllable excited-state optimization by single electronic effects, but also the potential of dual-emissive systems for practical display and lighting applications.

## Materials and Methods

Experimental details, thermal properties, DFT simulation, electrochemical and photophysical properties, and device performance are included in the Supplementary Materials.

## Data Availability

All data supporting the findings of this study are presented in the article and the Supplementary Materials. Additional data are available from the corresponding authors upon reasonable request.
