# Supplementary material for: Efficient Red Electroluminescent Copper Complexes with Fluorination-Balanced Dual Emission
Source: Research (Wash D C). 2026 Jan 14;9:1088. doi: 10.34133/research.1088 (PMC12799920; doi:10.34133/research.1088)
Supplement: Supplementary 1 — Figs. S1 to S65 Tables S1 to S12 [file research.1088.f1.zip › Research-Supplemental Information.docx]

Supplementary Information

Efficient Red Electroluminescent Copper Complexes with Fluorination-Balanced Dual Emission

Xinjing Lou^1^, Gang Chen^1^, Chunyu Liu^1^, Jing Zhang^1*^, Jiexu He^1^, Jixiu Niu^1^, Chunbo Duan^1^, Chunmiao Han^1^, Andrey A. Karasik^2^, and Hui Xu^1*^

^1^Key Laboratory of Functional Inorganic Material Chemistry (Ministry of Education), School of Chemistry and Material Science, Heilongjiang University, 74 Xuefu Road, Harbin 150080, P. R. China.

^2^Arbuzov Institute of Organic and Physical Chemistry, FRC Kazan Scientific Center, Russian Academy of Sciences, 8 Arbuzov Street, Kazan 420088, Russian Federation.

^*^Address correspondence to: zhangjing@hlju.edu.cn (J.Z.); hxu@hlju.edu.cn (H.X.)

**Content**

[Experimental Details 3](#_Toc212574305)

[Single Crystal Structures 22](#_Toc212574306)

[Thermal Properties 29](#_Toc212574311)

[DFT and TDDFT Simulations 30](#_Toc212574312)

[Electrochemical Properties 36](#_Toc212574316)

[Photophysical Properties 37](#_Toc212574317)

[Electroluminescent Performance 51](#_Toc212574320)

[References 86](#_Toc212574324)

### Experimental Details

*1. Materials and Instruments*

All the reagents and solvents used for synthesis were purchased from Aldrich and Acros companies and used without further purification.

^1^H NMR spectra were recorded using a Varian Mercury plus 400NB spectrometer and tetramethylsilane (TMS) as internal standard, CDCl_3_ as solvent. ^13^C NMR spectra were obtained on the same instrument at an operating frequency of approximately 100 MHz, using CDCl_3_ as solvent and TMS as internal reference. ^19^F NMR spectra were measured on the Varian Mercury plus 400 NB spectrometer at an operating frequency of approximately 376 MHz, using CDCl_3_ as solvent and CFCl_3_ as the external reference (*δ* = 0 ppm). The crystal suitable for single-crystal XRD analysis was obtained through slowly diffusing hexane into dichloromethane (DCM) solution of **nFTTPPCuI** at room temperature. All diffraction data were collected at 295 K on a Rigaku Xcalibur E diffractometer with graphite monochromatized Mo Kα (λ = 0.71073 Å) radiation in ω scan mode. All structures were solved by direct method and difference Fourier syntheses. Non-hydrogen atoms were refined by full-matrix least-squares techniques on F2 with anisotropic thermal parameters. The hydrogen atoms attached to carbons were placed in calculated positions with C-H = 0.93 Å and U (H) = 1.2Ueq (C) in the riding model approximation. All calculations were carried out with the SHELXL97 program. Absorption and photoluminescence (PL) emission spectra of the target compound were measured using a SHIMADZU UV-3150 spectrophotometer and a SHIMADZU RF-5301PC spectrophotometer, respectively. Thermogravimetric analysis (TGA) and differential scanning calorimetry (DSC) were performed on Shimadzu DSC-60A and DTG-60A thermal analyzers under nitrogen atmosphere at a heating rate of 10 °C min^-1^. Cyclic voltammograms (CV) studies were conducted using an Eco Chemie B. V. AUTOLAB potentiostat in a typical three-electrode cell with a glassy carbon working electrode, a platinum wire counter electrode, and a silver/silver chloride (Ag/AgCl) reference electrode. All electrochemical experiments were carried out under a nitrogen atmosphere at room temperature in DCM for oxidation and THF for reduction. Steady-state emission spectra were measured using an Edinburgh FPLS 1000 fluorescence spectrophotometer. Time-resolved emission spectra (TRES) were measured with Time-Correlated Single Photon Counting (TCSPC) method with a microsecond pulsed Xenon light source for 10 *μ*s-10 s lifetime measurement, the synchronization photomultiplier for signal collection and the Multi-Channel Scaling Mode of the PCS900 fast counter PC plug-in card for data processing. Spectra of prompt fluorescence (PF), delayed fluorescence (DF) and phosphorescence (PH) were sliced from TRES in the time ranges of <1 *μ*s, 1-200 *μ*s, and >200 *μ*s, respectively. PL quantum yields (PLQY, *ϕ*_PL_) of these films were measured through a labsphere 1-M-2 (*ϕ* = 6'') integrating sphere coated by Benflect with efficient light reflection in a wide range of 200-1600 nm, which was integrated with FLS 1000. The absolute PLQY determination of the sample was performed by two spectral (emission) scans, with the emission monochromator scanning over the Rayleigh scattered light from the sample and from a blank substrate. The first spectrum recorded the scattered light and the emission of the sample, and the second spectrum contained the scattered light of Benflect coating. The integration and subtraction of the scattered light parts in these two spectra arrived at the photon number absorbed by the samples (N_a_); while, integration of the emission of the samples to calculate the emissive photon number (N_e_). Then, the absolute PLQY (*η*) can be estimated according to the equation of *η* = N_e_/N_a_. Spectral correction (emission arm) was applied to the raw data after background subtraction, and from these spectrally corrected curves, *ϕ*_PL_ was calculated using aF900 software wizard.


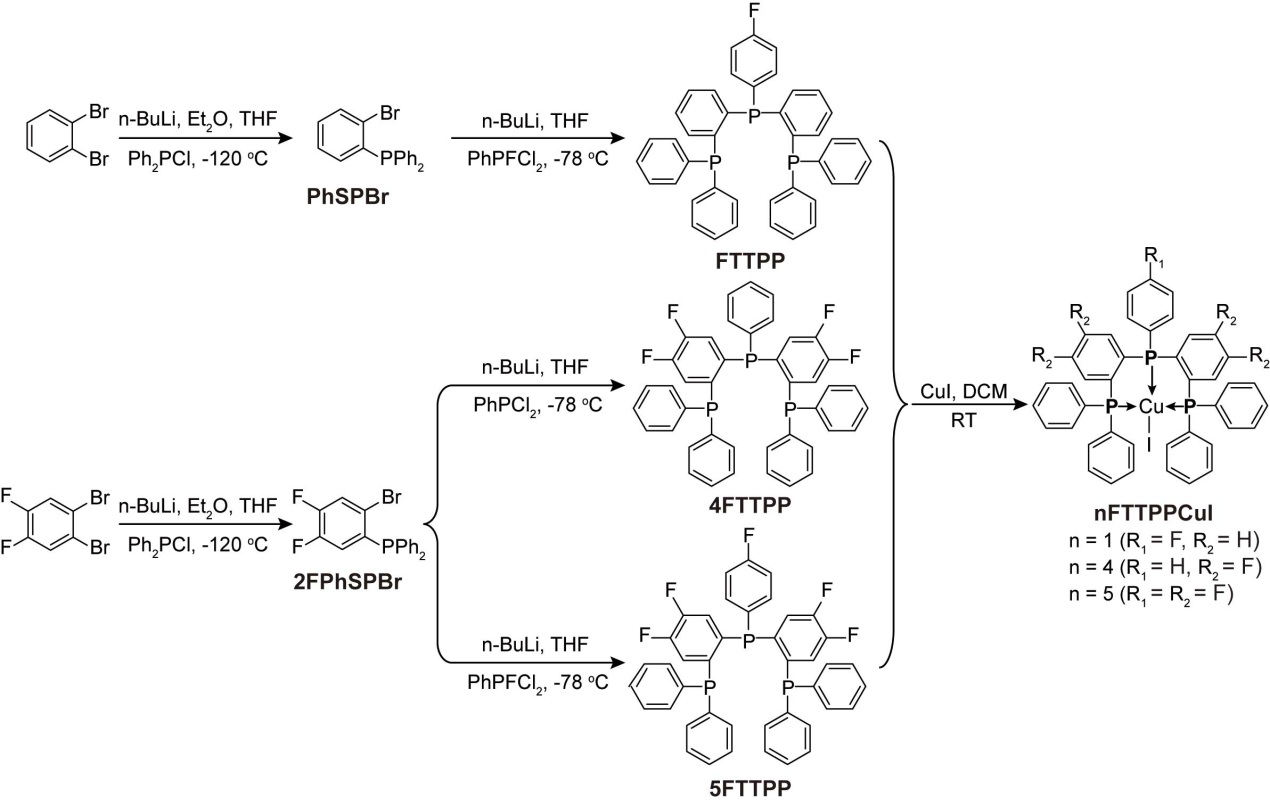


**Scheme S1.** Synthetic procedures of **FTTPPCuI**, **4FTTPPCuI**, and **5FTTPPCuI**.

*2. Synthesis Details*

**PhSPBr**

Under Ar, 236 mg (1 mmol) of 1,2-dibromobenzene and 1.80 mL of Et_2_O and 1.80 mL of THF were added to a three-necked flask and mixed by stirring. The mixture was cooled to -120 °C. Then, 0.40 mL (1 mmol) of *n*-BuLi in hexane solution was added in dropwise. After Stirring for half an hour, 0.18 mL (1 mmol) of Ph_2_PCl was added slowly and stirred for another half an hour at the same temperature, and then the system was naturally warmed to room temperature and stirred by 12 h. After quenched with ice water, the system was extracted with H_2_O and DCM. The organic layer was combined and dried over anhydrous Na_2_SO_4_ and the solvent was removed in vacuo. The mixture was purified with column chromatography to afford 255 mg of white powder with a yield of 75%.^1^H NMR (400 MHz, CDCl_3_) *δ* = 7.64-7.55 (m, 1H), 7.41-7.33 (m, 6H), 7.32-7.23 (m, 5H), 7.22-7.17 (m, 2H), 6.79-6.70 (m, 1H). LDI-TOF: m/z (%) 340 (100) [M^+^]; elemental analysis for C_18_H_14_BrP: calculated: C 63.37, H 4.14; found: C 63.28, H 4.11.

**2FPhSPBr**

Under Ar, 270 mg (1 mmol) of 1,2-dibromo-4,5-difluorobenzene and 1.80 mL of Et_2_O and 1.80 mL of THF were added to a three-necked flask and mixed by stirring. The mixture was cooled to -120 °C. Then, 0.40 mL (1 mmol) of *n*-BuLi in hexane solution was added in dropwise. After Stirring for half an hour, 0.18 mL (1 mmol) of Ph_2_PCl was added slowly and stirred for another half an hour at the same temperature, and then the system was naturally warmed to room temperature and stirred by 12 h. After quenched with ice water, the system was extracted with H_2_O and DCM. The organic layer was combined and dried over anhydrous Na_2_SO_4_ and the solvent was removed in vacuo. The mixture was purified with column chromatography to afford 260 mg of white powder with a yield of 69%. ^1^H NMR (400 MHz, CDCl_3_) *δ* = 7.48-7.41 (m, 1H), 7.37 (dt, *J* = 3.7, 3.2 Hz, 6H), 7.26 (td, *J* = 7.7, 1.6 Hz, 5H), 6.62-6.53 (m, 1H). ^13^C NMR (100 MHz, CDCl_3_) *δ* = 136.55, 136.51, 136.49, 136.39, 136.32, 135.18, 135.07, 134.07, 133.86, 129.56, 129.06, 128.99, 122.87, 122.68, 122.34, 122.32, 122.15, 122.12 ppm. ^19^F NMR (376 MHz, CDCl_3_) *δ* = -137.64, -137.69 ppm. LDI-TOF: m/z (%) 376 (100) [M^+^]; elemental analysis for C_18_H_12_BrF_2_P: calculated: C 57.32, H 3.21; found: C 57.18, H 3.19.

**FTTPP**

Under Ar, 340 mg (1 mmol) of PhSPBr was dissolved in 5 mL of dry THF. The solution was cooled to -78 °C. Then, 0.48 mL (1.20 mmol) of *n*-BuLi in hexane solution was added in dropwise. The mixture was stirred at -78 °C for half an hour. Then, 0.10 g (0.50 mmol) of PhPFCl_2_ was added slowing, and the system was stirred at -78 °C for half an hour. The system was further stirred at room temperature for 12 h, and then quenched by ice water. After extraction with H_2_O and DCM, the organic layer was combined and dried over anhydrous Na_2_SO_4_, and the solvent was removed in vacuo. The mixture was purified with column chromatography to afford 300 mg of white powder with a yield of 46%. ^1^H NMR (400 MHz, CDCl_3_) *δ* = 7.27-7.11 (m, 19H), 7.07 (d, *J* = 6.0 Hz, 10H), 6.81 ppm (s, 4H). ^13^C NMR (100 MHz, CDCl_3_) *δ* = 143.55, 143.45, 143.36, 143.25, 143.15, 143.05, 142.27, 142.23, 142.19, 142.16, 141.96, 141.92, 141.89, 141.85, 136.38, 136.33, 136.26, 136.21, 136.09, 136.03, 135.63, 135.55, 135.41, 135.33, 133.53, 133.47, 133.21, 133.17, 133.12, 132.92, 132.87, 132.82, 132.77, 132.72, 132.68, 127.89, 127.72, 127.15, 127.12, 114.43, 114.35, 114.22, 114.14 ppm. ^19^F NMR (376 MHz, CDCl_3_) *δ* = -113.31 ppm. LDI-TOF: m/z (%) 648 (100) [M^+^]; elemental analysis for C_42_H_32_FP_3_: calculated: C 77.77, H 4.97; found: C 77.38, H 4.85.

**4FTTPP**

Under Ar, 376 mg (1 mmol) of 2FPhSPBr was dissolved in 5 mL of dry THF. The solution was cooled to -78 °C. Then, 0.48 mL (1.20 mmol) of n-BuLi in hexane solution was added in dropwise. The mixture was stirred at -78 °C for half an hour. Then, 0.09 mL (0.50 mmol) of PhPCl_2_ was added slowing, and the system was stirred at -78 °C for half an hour. The system was further stirred at room temperature for 12 h, and then quenched by ice water. After extraction with H_2_O and DCM, the organic layer was combined and dried over anhydrous Na_2_SO_4_, and the solvent was removed in vacuo. The mixture was purified with column chromatography to afford 346 mg of white powder with a yield of 49%. ^1^H NMR (400 MHz, CDCl_3_) *δ =* 7.35-7.22 (m, 8H), 7.18 (dt, *J* = 13.6, 6.9 Hz, 12H), 7.04 (dd, *J* = 12.7, 6.8 Hz, 6H), 6.83 (t, *J* = 8.9 Hz, 2H), 6.52 (t, *J* = 9.2 Hz, 2H). ^13^C NMR (100 MHz, CDCl_3_) *δ =* 136.45, 136.39, 136.34, 136.28, 135.76, 135.71, 135.64, 135.59, 134.25, 134.05, 133.98, 133.95, 133.84, 133.74, 133.71, 133.67, 133.58, 133.48, 133.44, 133.40, 129.13, 128.88, 128.71, 128.66, 128.64, 128.54, 128.50, 128.47, 128.44, 128.41, 128.37, 123.35, 123.28, 123.23, 123.19, 123.18, 123.12, 123.11, 123.06, 123.02, 122.97 ppm. ^19^F NMR (376 MHz, CDCl_3_) *δ =* -135.73, -135.79, -136.33, -136.39 ppm. LDI-TOF: m/z (%) 702 (100) [M^+^]; elemental analysis for C_42_H_29_F_4_P_3_: calculated: C 71.80, H 4.16; found: C 71.24, H 4.12.

**5FTTPP**

Under Ar, 376 mg (1 mmol) of 2FPhSPBr was dissolved in 5 mL of dry THF. The solution was cooled to -78 °C. Then, 0.48 mL (1.20 mmol) of *n*-BuLi in hexane solution was added in dropwise. The mixture was stirred at -78 °C for half an hour. Then, 0.10 g (0.50 mmol) of PhPFCl_2_ was added slowing, and the system was stirred at -78 °C for half an hour. The system was further stirred at room temperature for 12 h, and then quenched by ice water. After extraction with H_2_O and DCM, the organic layer was combined and dried over anhydrous Na_2_SO_4_, and the solvent was removed in vacuo. The mixture was purified with column chromatography to afford 350 mg of white powder with a yield of 49%. ^1^H NMR (400 MHz, CDCl_3_) *δ* = 7.33-7.21 (m, 10H), 7.16 (dd, *J* = 14.0, 6.7 Hz, 8H), 7.08-6.97 (m, 6H), 6.84 (t, *J* = 8.4 Hz, 3H), 6.56-6.44 (m, 2H). ^13^C NMR (100 MHz, CDCl_3_) *δ* = 136.47, 136.39, 136.24, 136.16, 136.07, 136.01, 135.63, 135.57, 135.51, 135.46, 133.91, 133.80, 133.77, 133.75, 133.72, 133.70, 133.63, 133.56, 133.53, 133.48, 128.93, 128.73, 128.57, 128.54, 128.51, 128.47, 128.44, 128.41, 123.45, 123.38, 123.28, 123.21, 123.06, 123.00, 122.96, 122.89, 122.84, 122.80, 116.05, 115.97, 115.83, 115.76 ppm. ^19^F NMR (376 MHz, CDCl_3_) *δ* = -111.53, -135.52, -135.57, -136.10, -136.15 ppm. LDI-TOF: m/z (%) 720 (100) [M^+^]; elemental analysis for C_42_H_28_F_5_P_3_: calculated: C 70.01, H 3.92; found: C 69.85, H 3.78.

**FTTPPCuI**

Under Ar, 648 mg (1 mmol) of FTTPP and 190 mg (1 mmol) of CuI were dissolved in 5 mL of CH_2_Cl_2_. The solution was stirred for 3 h at room temperature (RT). Then, the solvent was evaporated to obtain crude material, which was further recrystallized from CH_2_Cl_2_/*n*-hexane solution to afford yellow crystal of 714 mg with a yield of 85%. ^1^H NMR (400 MHz, CDCl_3_) *δ* = 7.85 (s, 4H), 7.53 (s, 2H), 7.40 (t, *J* = 7.3 Hz, 2H), 7.36-7.23 (m, 11H), 7.20 (t, *J* = 7.3 Hz, 2H), 7.13 (d, *J* = 3.5 Hz, 2H), 7.03 (t, *J* = 7.5 Hz, 4H), 6.92 (t, *J* = 8.4 Hz, 2H), 6.68 (s, 4H). ^13^C NMR (100 MHz, CDCl_3_) *δ* = 135.05, 134.97, 134.82, 134.75, 134.67, 134.21, 134.05, 133.89, 133.83, 133.73, 133.32, 133.28, 133.24, 132.40, 132.35, 132.30, 130.44, 130.10, 130.07, 129.97, 129.83, 129.80, 129.67, 128.97, 128.81, 128.67, 128.55, 128.46, 128.36, 128.33, 128.22, 128.18, 128.14, 127.92, 127.83, 127.74, 116.01, 115.91, 115.80, 115.70 ppm. ^19^F NMR (376 MHz, CDCl_3_) *δ* = -110.70 ppm. LDI-TOF: m/z (%) 838 (100) [M^+^]; elemental analysis for C_42_H_32_CuFIP_3_: calculated: C 60.12, H 3.84; found: C 59.85, H 3.79. CCDC No. 2395226.

**4FTTPPCuI**

Under Ar, 702 mg (1 mmol) of 4FTTPP and 190 mg (1 mmol) of CuI were dissolved in 5 mL of CH_2_Cl_2_. The solution was stirred for 3 h at RT. Then, the solvent was evaporated to obtain crude material, which was further recrystallized from CH_2_Cl_2_/*n*-hexane solution to afford yellow crystal of 768 mg with a yield of 86%. ^1^H NMR (400 MHz, CDCl_3_) *δ* = 7.80 (d, *J* = 4.5 Hz, 4H), 7.38-7.20 (m, 16H), 7.15 (t, *J* = 7.5 Hz, 4H), 6.97-6.86 (m, 2H), 6.80 (s, 4H). ^13^C NMR (100 MHz, CDCl_3_) *δ* = 134.64, 134.57, 134.49, 132.80, 132.79, 132.77, 132.62, 132.61, 132.58, 132.55, 132.54, 132.52, 132.51, 132.46, 132.46, 132.41, 132.39, 130.62, 130.61, 130.59, 130.54, 130.28, 129.67, 129.16, 129.12, 129.09, 129.06, 129.03, 129.00, 128.76, 128.71, 128.66, 128.61, 128.57, 128.53, 122.65, 122.54, 122.48, 122.37, 121.96, 121.90, 121.83, 121.79 ppm. ^19^F NMR (376 MHz, CDCl_3_) *δ* = -131.64, -131.70, -132.16, -132.21 ppm. LDI-TOF: m/z (%) 892 (100) [M^+^]; elemental analysis for C_42_H_29_CuF_4_IP_3_: calculated: C 56.49, H 3.27; found: C 55.85, H 3.19. CCDC No. 2395227.

**5FTTPPCuI**

Under Ar, 720 mg (1 mmol) of 5FTTPP and 190 mg (1 mmol) of CuI were dissolved in 5 mL of CH_2_Cl_2_. The solution was stirred for 3 h at RT. Then, the solvent was evaporated to obtain crude material, which was further recrystallized from CH_2_Cl_2_/*n*-hexane solution to afford yellow crystal of 788 mg with a yield of 86%. ^1^H NMR (400 MHz, CDCl_3_) *δ* = 7.79 (dd, *J* = 10.4, 6.7 Hz, 4H), 7.39-7.27 (m, 13H), 7.18 (t, *J* = 7.5 Hz, 4H), 7.00 (t, *J* = 8.0 Hz, 2H), 6.94-6.87 (m, 2H), 6.84 (t, *J* = 8.5 Hz, 4H). ^13^C NMR (100 MHz, CDCl_3_) *δ* = 140.04, 139.85, 139.73, 139.47, 139.33, 137.00, 136.75, 136.52, 136.33, 134.31, 134.22, 134.13, 134.04, 133.60, 133.53, 133.45, 131.54, 131.47, 131.40, 131.01, 130.95, 130.85, 130.79, 130.69, 130.58, 130.47, 130.36, 129.57, 128.85, 127.69, 127.65, 127.61, 121.74, 121.63, 121.57, 121.52, 120.87, 120.70, 115.64, 115.54, 115.42, 115.32 ppm. ^19^F NMR (376 MHz, CDCl_3_) *δ* = -108.64, -131.45, -131.50, -132.02, -132.08 ppm. LDI-TOF: m/z (%) 911 (100) [M^+^]; elemental analysis for C_42_H_28_CuF_5_IP_3_: calculated: C 55.37, H 3.10; found: C 54.96, H 3.05. CCDC No. 2395225.

*3. DFT and TDDFT Calculation*

Density functional theory (DFT) and time-dependent DFT (TDDFT) computations were carried out with different parameters for structure optimizations and vibration analyses. The ground state (S_0_) configuration was established according to single crystal data. The S_0_, singlet and triplet states in vacuum were simulated by the restricted and unrestricted formalism of Beck's three-parameter hybrid exchange functional[1] and Lee, and Yang and Parr correlation functional[2] B3LYP/6-31G(d, p) for C, H and P, and the LANL2DZ effective core potentials and valence basis set for Cu and I[3], respectively. The fully optimized stationary points were further characterized by harmonic vibrational frequency analysis to ensure that real local minima had been found without imaginary vibrational frequency. The total energies were also corrected by zero-point energy both for the ground state and triplet state. Natural transition orbital (NTO) analysis was performed on the basis of optimized ground-state geometries at the same level [4]. The contours were visualized with Gauss view 5.0. All computations were performed using the Gaussian 09 package [5].

*4. Transition Parameter Calculation*

The calculation formulas for the rate constants of prompt fluorescence (*k*_PF_), delayed fluorescence (*k*_DF_), singlet radiation ($k_{r}^{S}$), singlet ($k_{nr}^{S}$), and triplet nonradiation ($k_{nr}^{T}$), reverse intersystem crossing (*k*_RISC_) and intersystem crossing (*k*_ISC_), and corresponding quantum efficiencies (*ϕ*) are expressed as following list: [6, 7]

$$k_{\mathrm{PF}}=k_{r}^{S}+k_{nr}^{S}+k_{\mathrm{ISC}} (\mathrm{Eq}.S1)$$

$$k_{\mathrm{DF}}=k_{nr}^{T}+\left( 1-\frac{k_{\mathrm{ISC}}}{k_{\mathrm{PF}}} \right)\cdot k_{\mathrm{RISC}}(\mathrm{Eq}.S2)$$

$$k_{r}^{S}=\phi_{\mathrm{PF}}\cdot k_{\mathrm{PF}} (\mathrm{Eq}.S3)$$

$$k_{nr}^{S}=k_{\mathrm{PF}}-k_{r}^{S}-k_{\mathrm{ISC}}=k_{\mathrm{PF}}-k_{r}^{S}-k_{\mathrm{PF}}\cdot\frac{\eta_{\mathrm{PF}}}{n}(\mathrm{Eq}.S4)$$

$$k_{\mathrm{PF}}=k_{r}^{S}+k_{nr}^{S}+k_{\mathrm{ISC}} (\mathrm{Eq}.S5)$$

$$k_{\mathrm{ISC}}=\left( 1-\phi_{PF} \right)\cdot k_{\mathrm{PF}} (\mathrm{Eq}.S6)$$

$$k_{nr}^{T}=k_{\mathrm{DF}}-\left( 1-\frac{k_{\mathrm{ISC}}}{k_{\mathrm{PF}}} \right)\cdot k_{\mathrm{RISC}}=k_{\mathrm{DF}}-\left( 1-\frac{k_{\mathrm{ISC}}}{k_{\mathrm{PF}}} \right)\cdot\frac{k_{\mathrm{DF}}\cdot k_{\mathrm{PF}}\cdot\phi_{\mathrm{DF}}}{k_{\mathrm{ISC}}\cdot\phi_{\mathrm{PF}}}(\mathrm{Eq}.S7)$$

*5.* *Emission Composition Estimation*

The proportions of thermal activated delayed fluorescence (TADF) and PH in emissions can be quantitatively evaluated with their fractional emission intensities *I*_PH_ and *I*_TADF_, which are functions of temperature:

$$\begin{aligned} \frac{I_{\mathrm{PH}}}{I_{\mathrm{tot}}}=\frac{1}{1+\frac{\tau_{T}}{\tau_{S}}.\exp\left( -\frac{\Delta E_{\mathrm{ST}}}{k_{B}T} \right)} (Eq.S8) \end{aligned}$$

$$\begin{aligned} \frac{I_{\mathrm{TADF}}}{I_{\mathrm{tot}}}=1-\frac{I_{\mathrm{PH}}}{I_{\mathrm{tot}}} (Eq.S9) \end{aligned}$$

where *I* is emission intensity, *T* is temperature, and *τ*_S_ and *τ*_T_ refer to the singlet and triplet lifetimes. Subscripts of “tot” refer to total emission. Δ*E*_ST_ is singlet-triplet splitting energy. *k*_B_ is Boltzmann constant.[8]

*6. Device Fabrication and Testing*

Before loading into a deposition chamber, the ITO substrate was cleaned with detergents and deionized water, dried in an oven at 120 °C for 4 h, and treated with UV-ozone for 20 min. Devices were fabricated by evaporating organic layers at a rate of 0.1-0.3 nm s^-1^ onto the ITO substrate sequentially at a pressure below 1×10^-6^ Pa. Onto the electron transporting layer, a layer of LiF with 1 nm thickness was deposited at a rate of 0.1 nm s^-1^ to improve electron injection. Finally, a 100 nm thick layer of Al was deposited at a rate of 0.6 nm s^-1^ as the cathode. The emission area of the devices was 0.09 cm^2^ as determined by the overlap area of the anode and the cathode. After fabrication, the devices were immediately transferred to a glove box for encapsulation with glass cover slips using epoxy glue. The EL spectra and CIE coordinates were measured using a PR655 spectra colorimeter. The current-density-voltage and brightness-voltage curves of the devices were measured using a Keithley 4200 source meter and a calibrated silicon photodiode. All the measurements were carried out at room temperature in glove box.

*6. ^1^H NMR, ^13^C NMR, and ^19^F NMR Spectra*


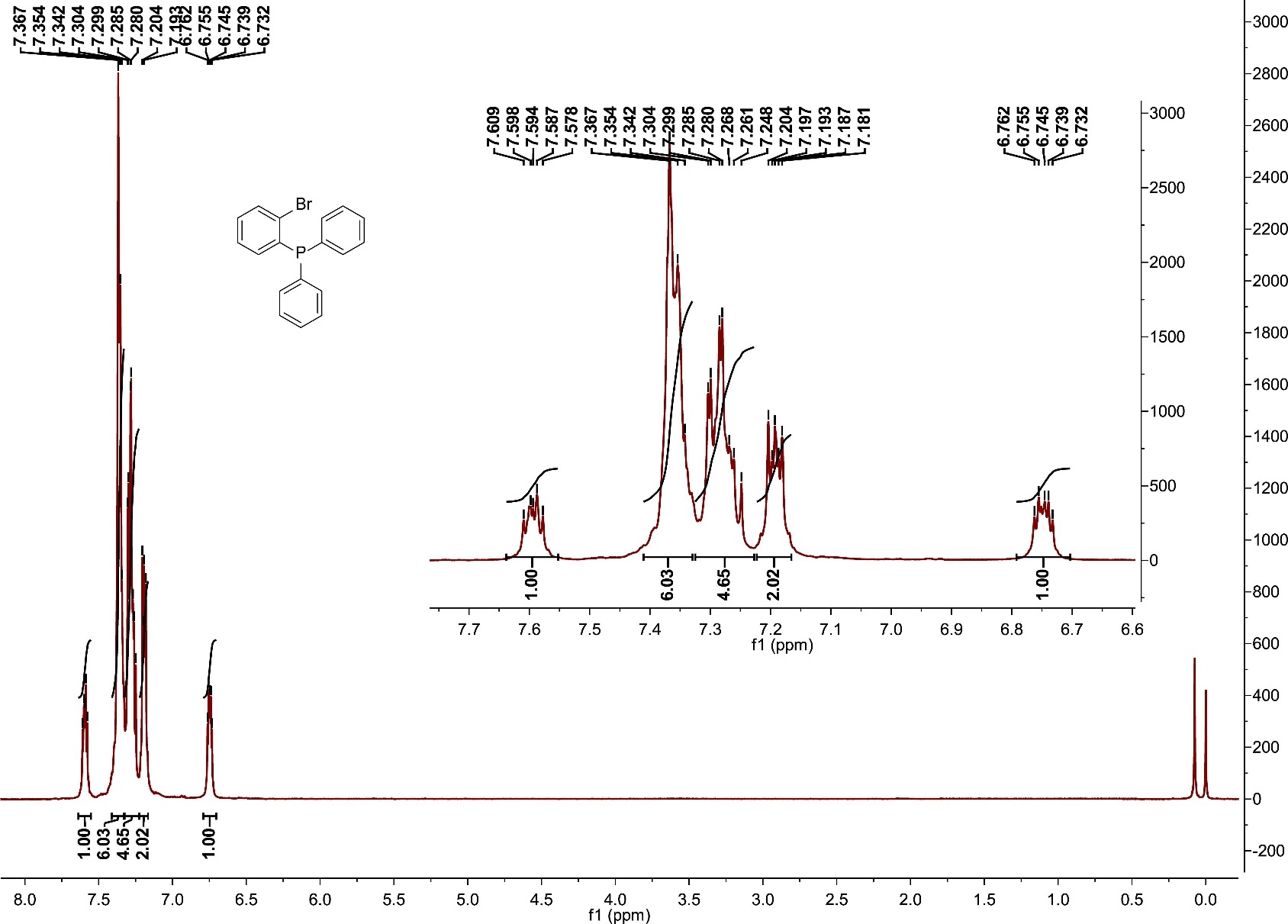


**Fig.** **S1.** ^1^H NMR (400 MHz) spectrum of **PhSPBr** in CDCl_3_.


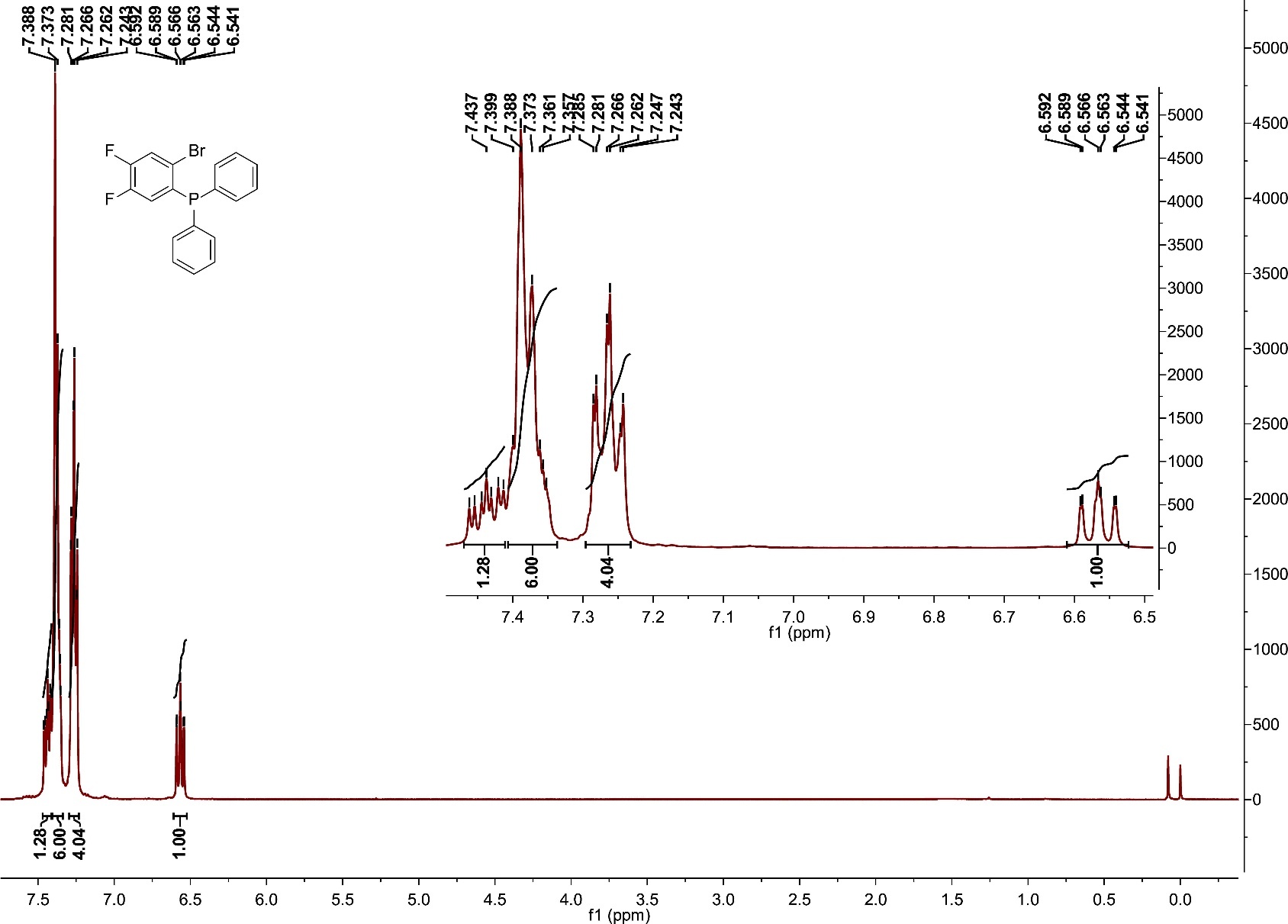


**Fig. S2.** ^1^H NMR (400 MHz) spectrum of **2FPhSPBr** in CDCl_3_.


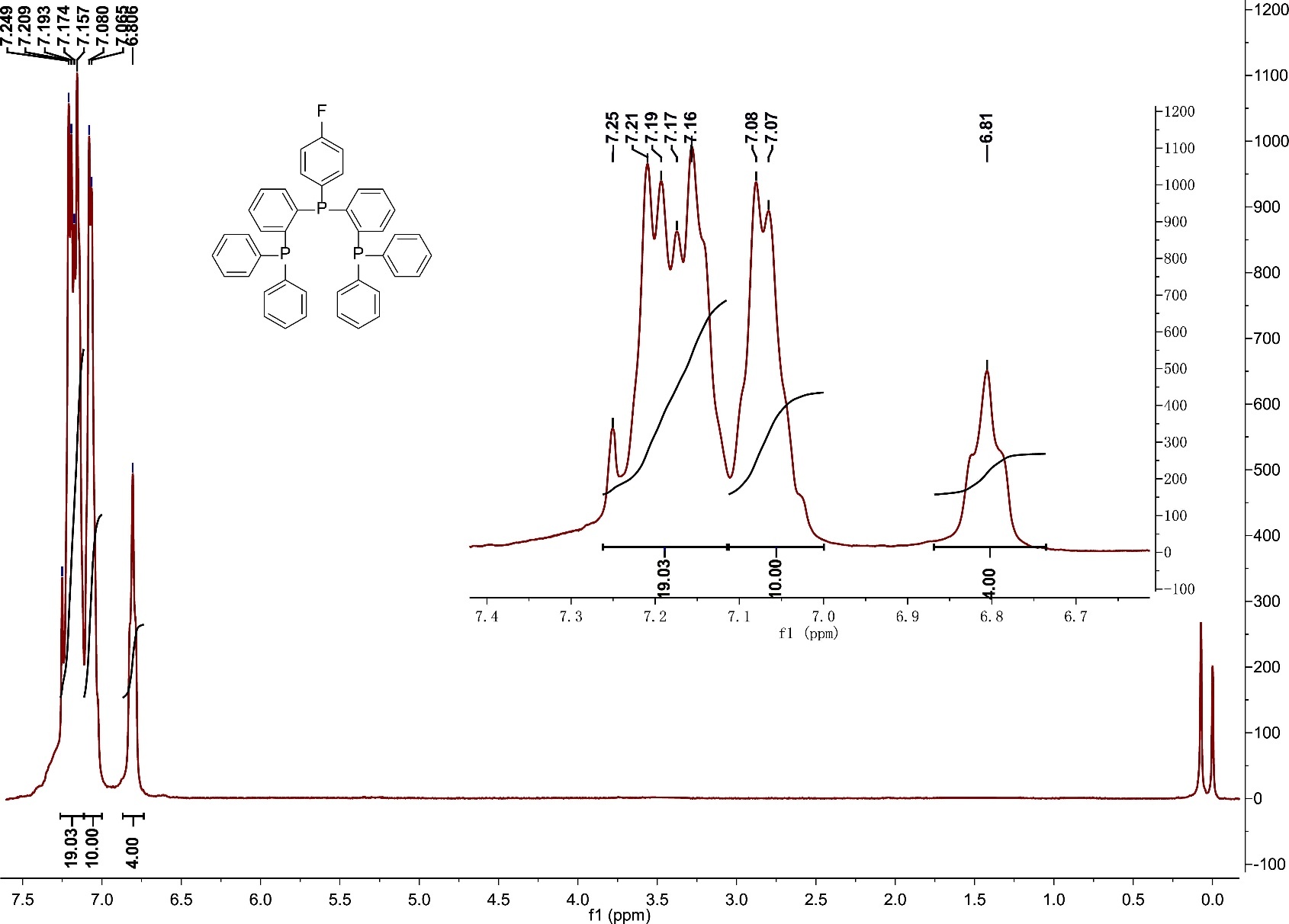


**Fig. S3.** ^1^H NMR (400 MHz) spectrum of **FTTPP** in CDCl_3_.


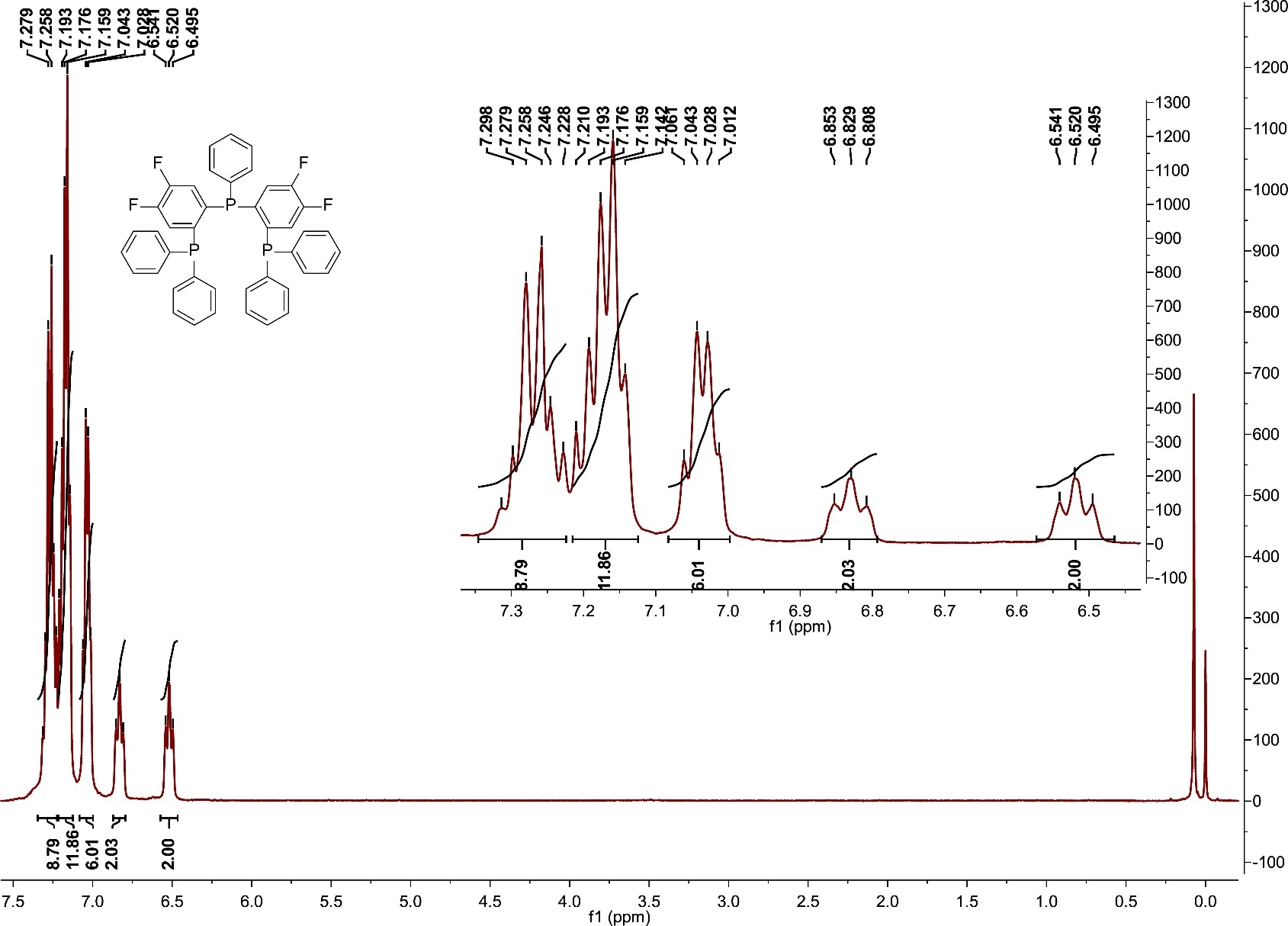


**Fig. S4.** ^1^H NMR (400 MHz) spectrum of **4FTTPP** in CDCl_3_.


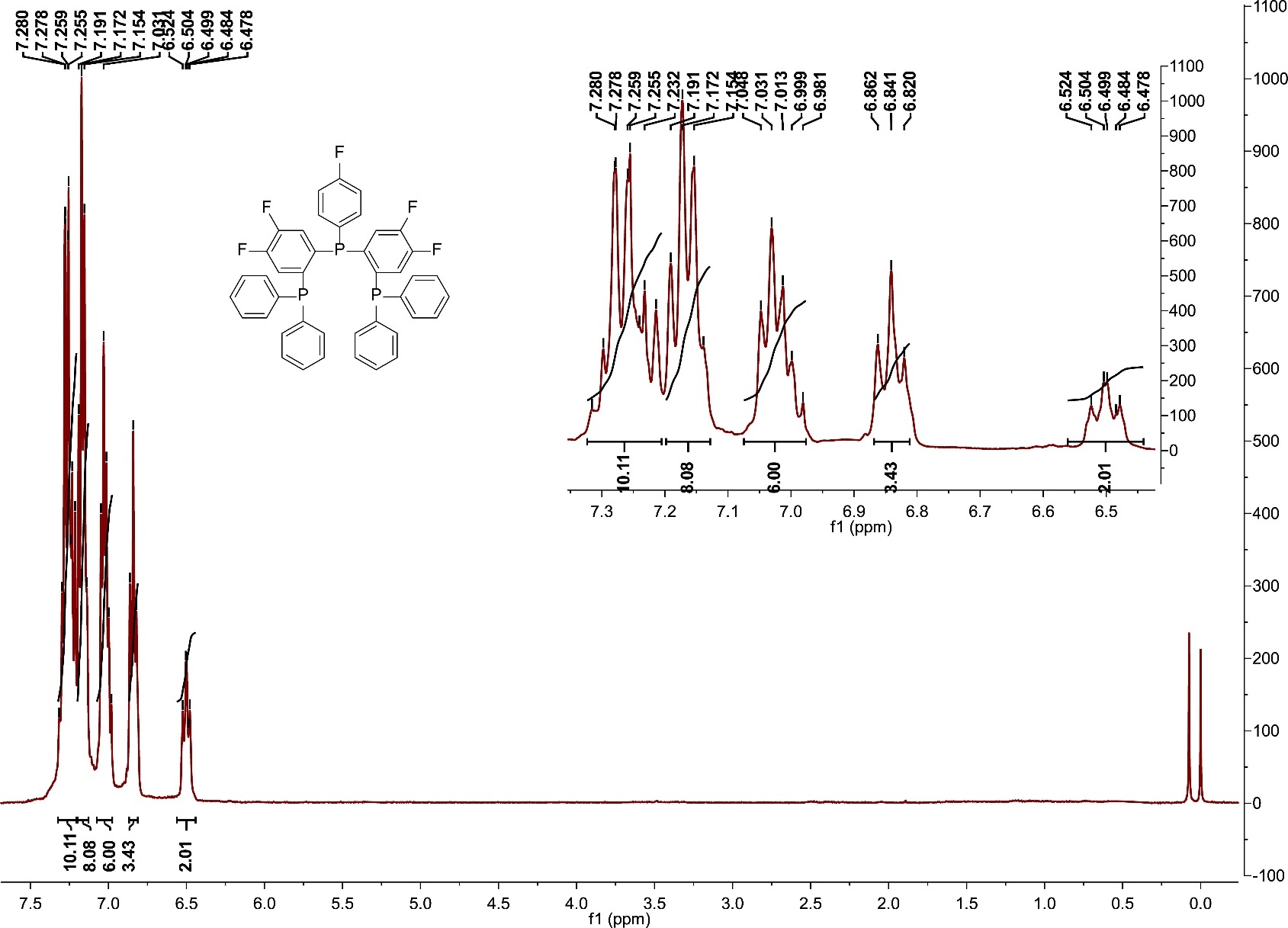


**Fig. S5.** ^1^H NMR (400 MHz) spectrum of **5FTTPP** in CDCl_3_.


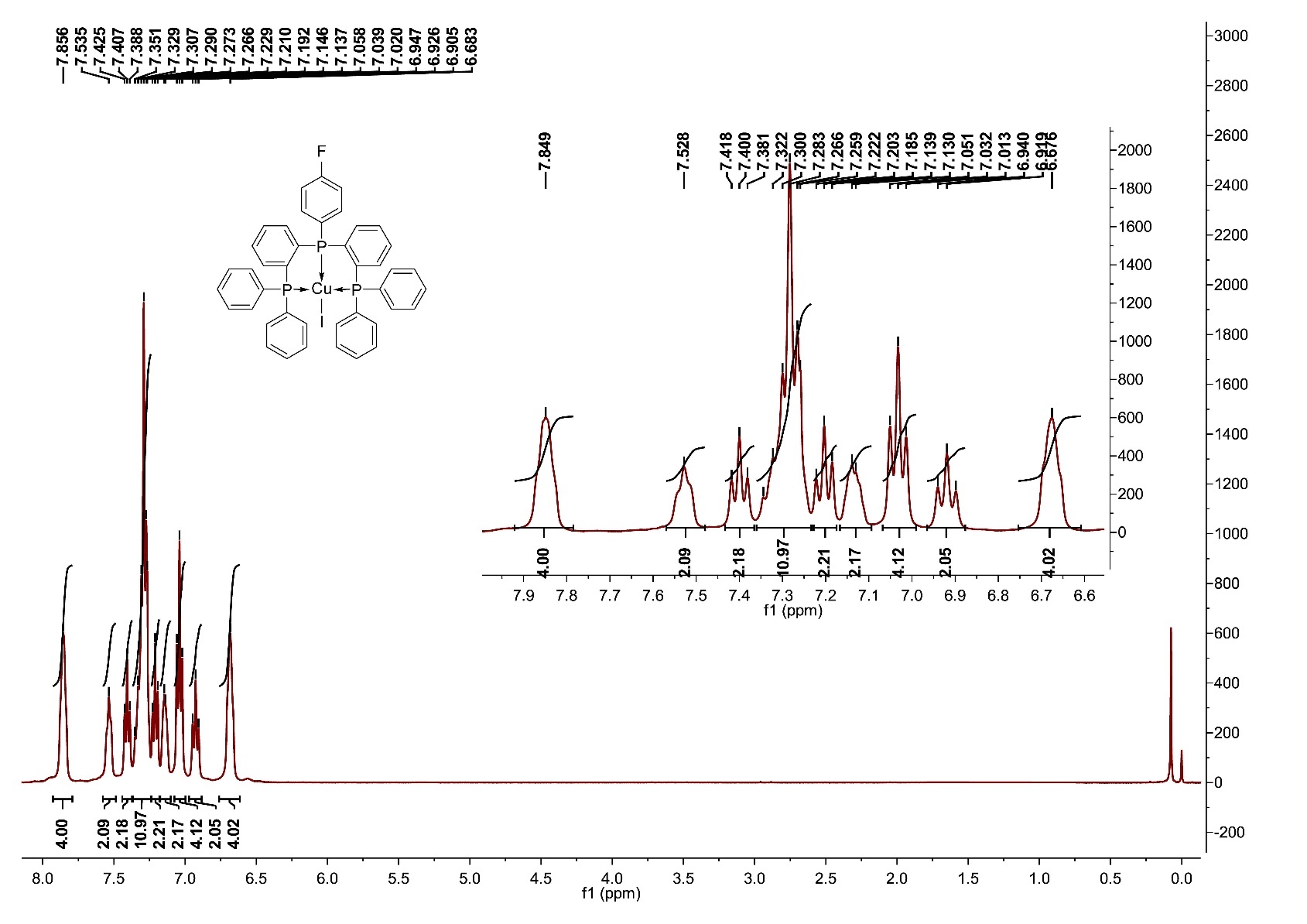


**Fig. S6.** ^1^H NMR (400 MHz) spectrum of **FTTPPCuI** in CDCl_3_.


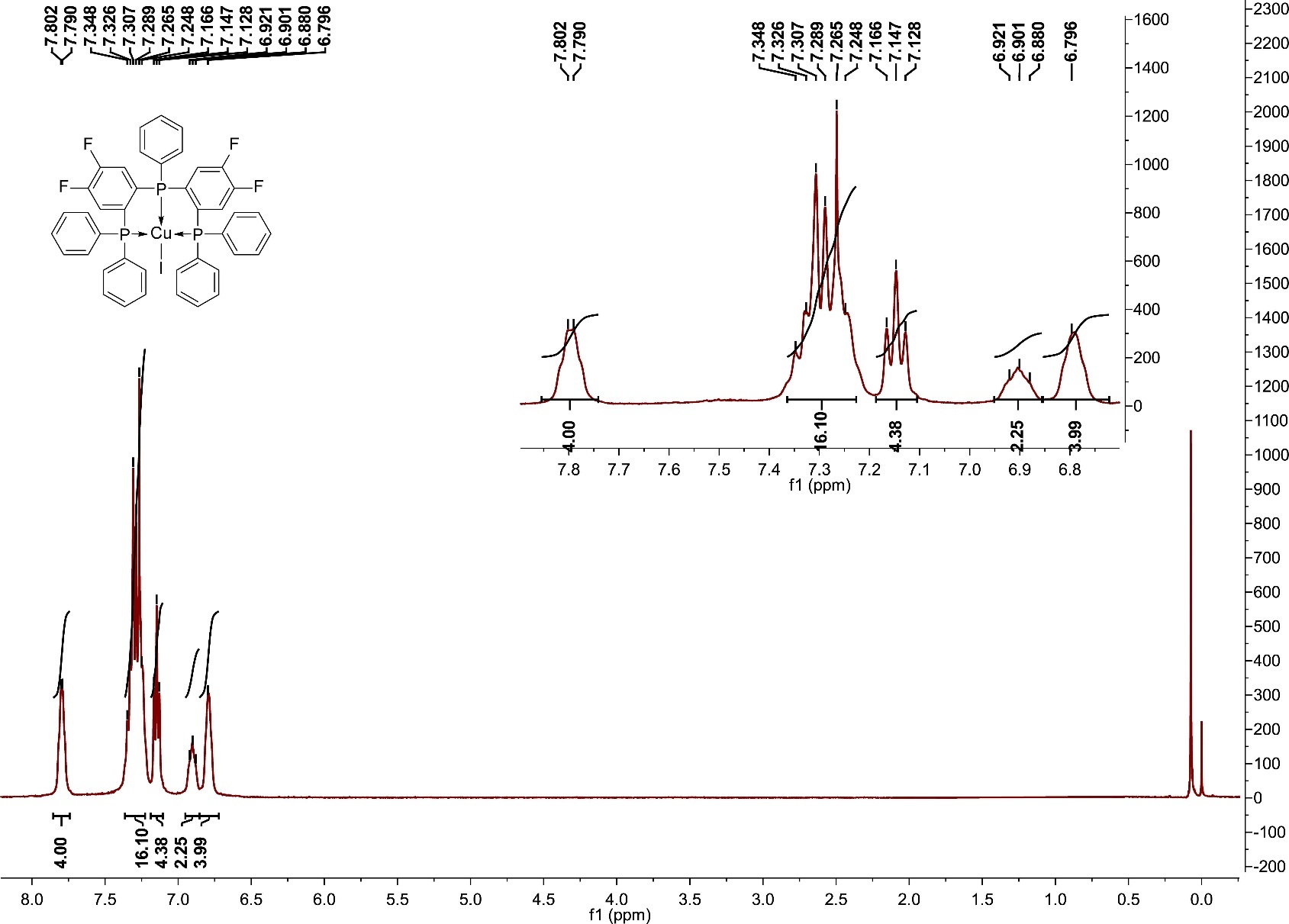


**Fig. S7.** ^1^H NMR (400 MHz) spectrum of **4FTTPPCuI** in CDCl_3_.


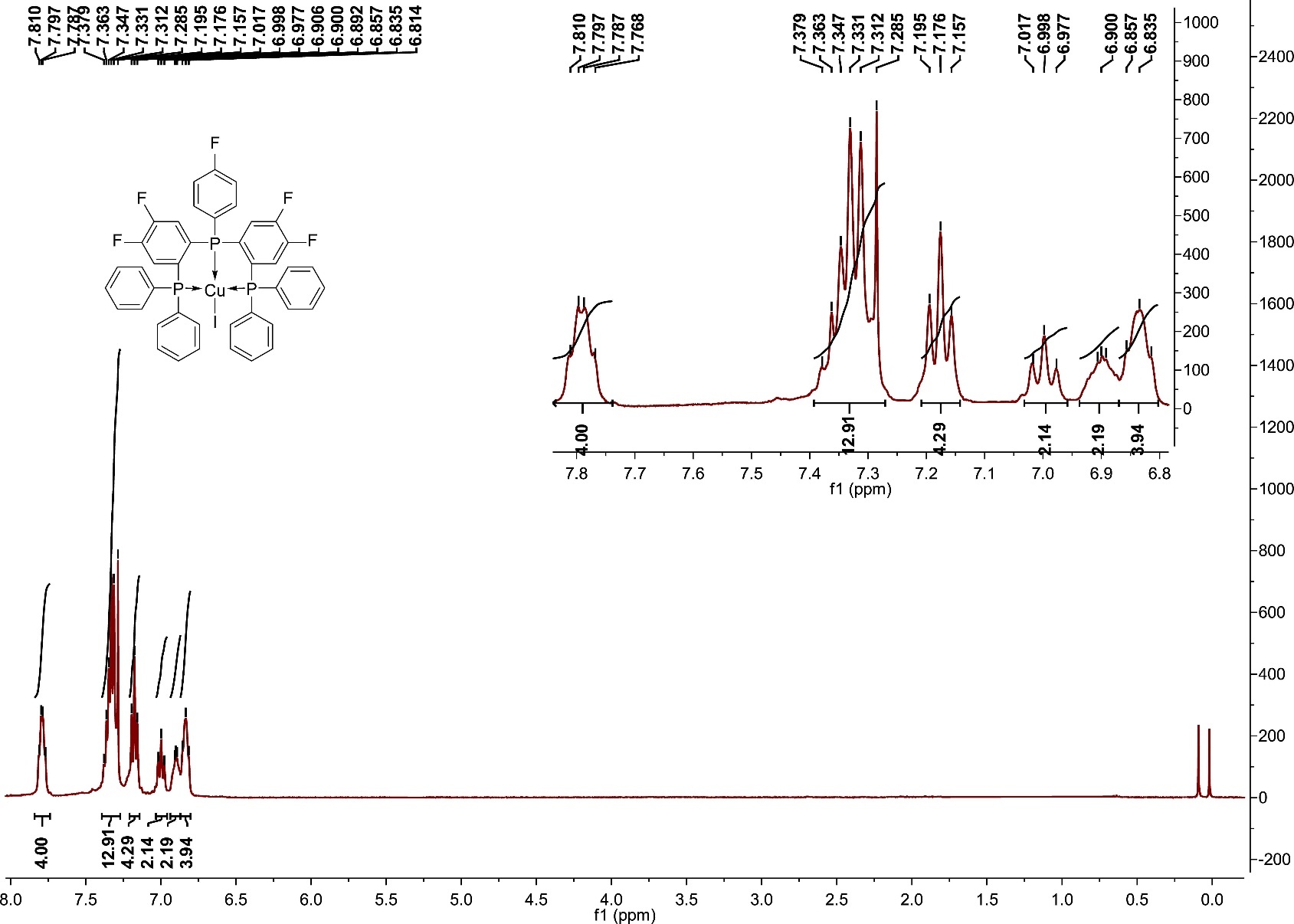


**Fig. S8.** ^1^H NMR (400 MHz) spectrum of **5FTTPPCuI** in CDCl_3_.


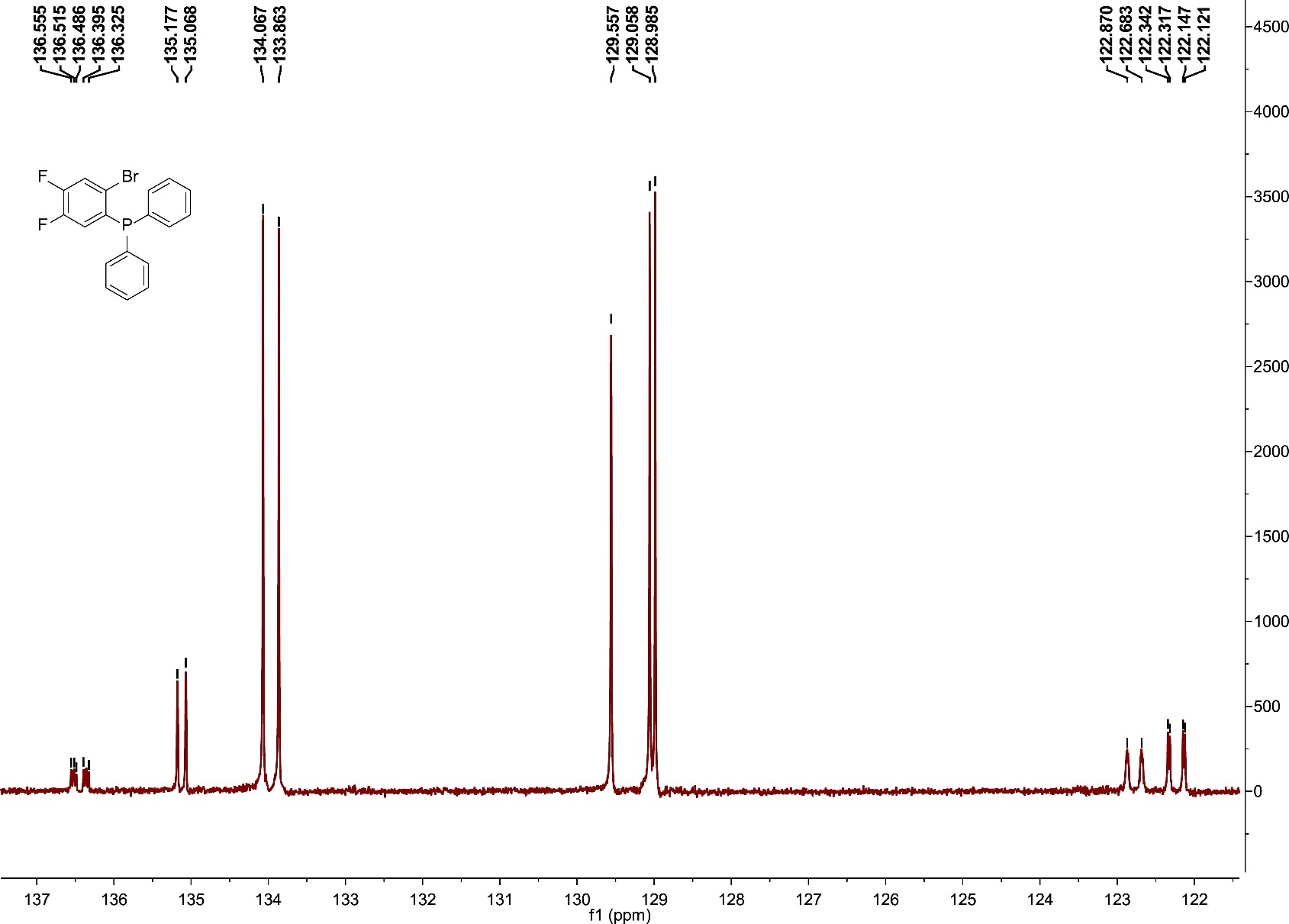


**Fig. S9.** ^13^C NMR (100 MHz) spectrum of **2FPhSPBr** in CDCl_3_.


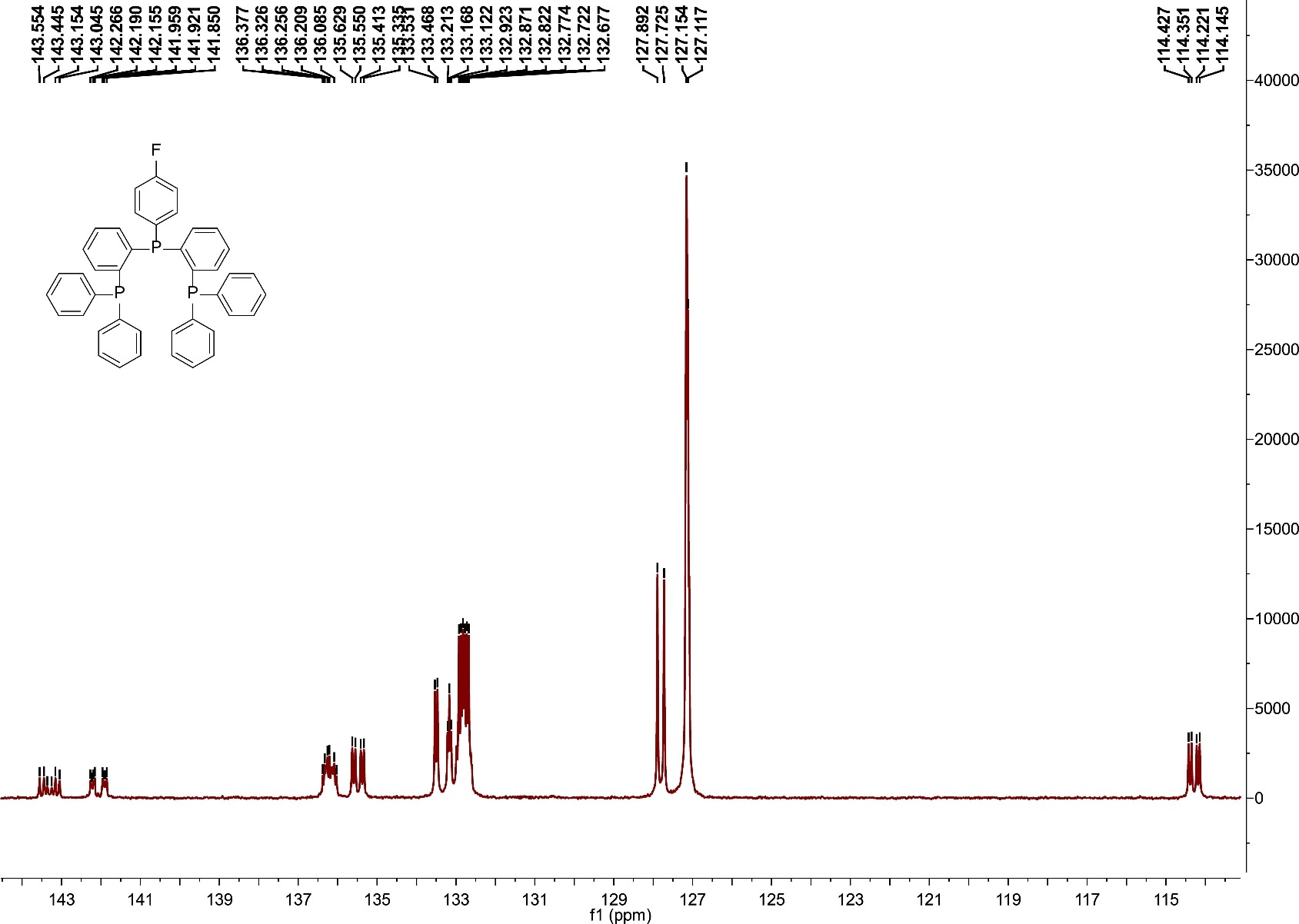


**Fig. S10.** ^13^C NMR (100 MHz) spectrum of **FTTPP** in CDCl_3_.


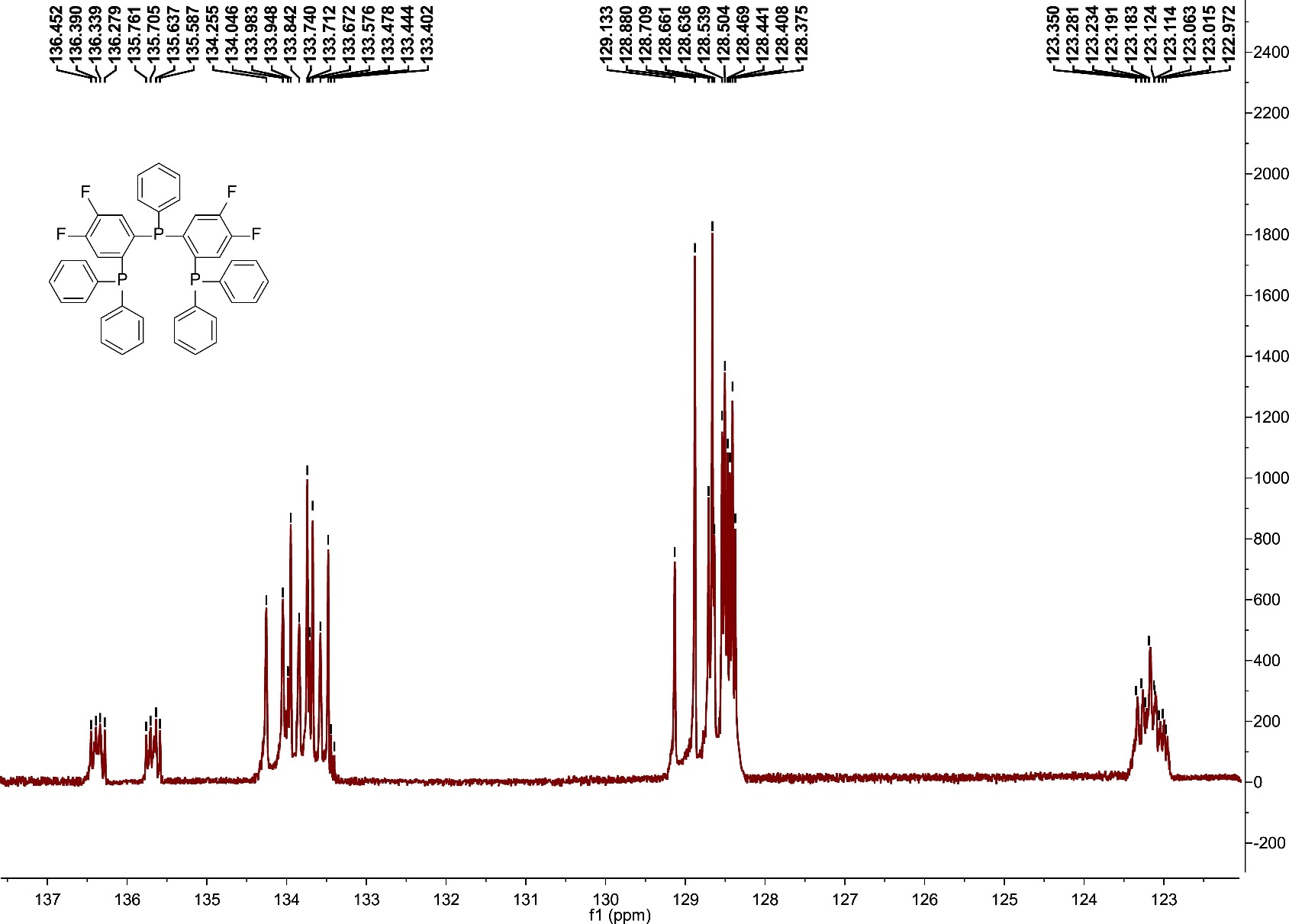


**Fig. S11.** ^13^C NMR (100 MHz) spectrum of **4FTTPP** in CDCl_3_.


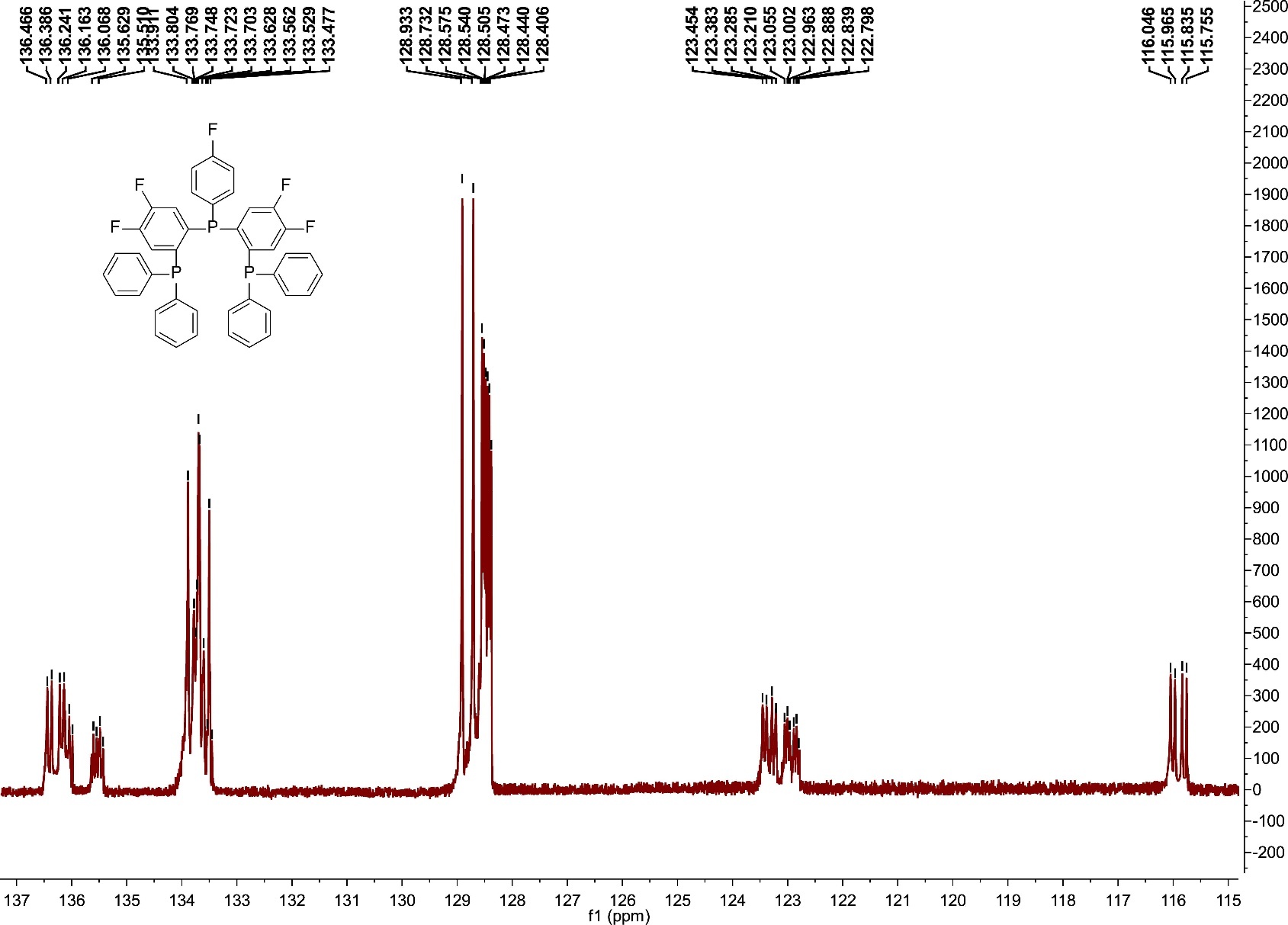


**Fig. S12.** ^13^C NMR (100 MHz) spectrum of **5FTTPP** in CDCl_3_.


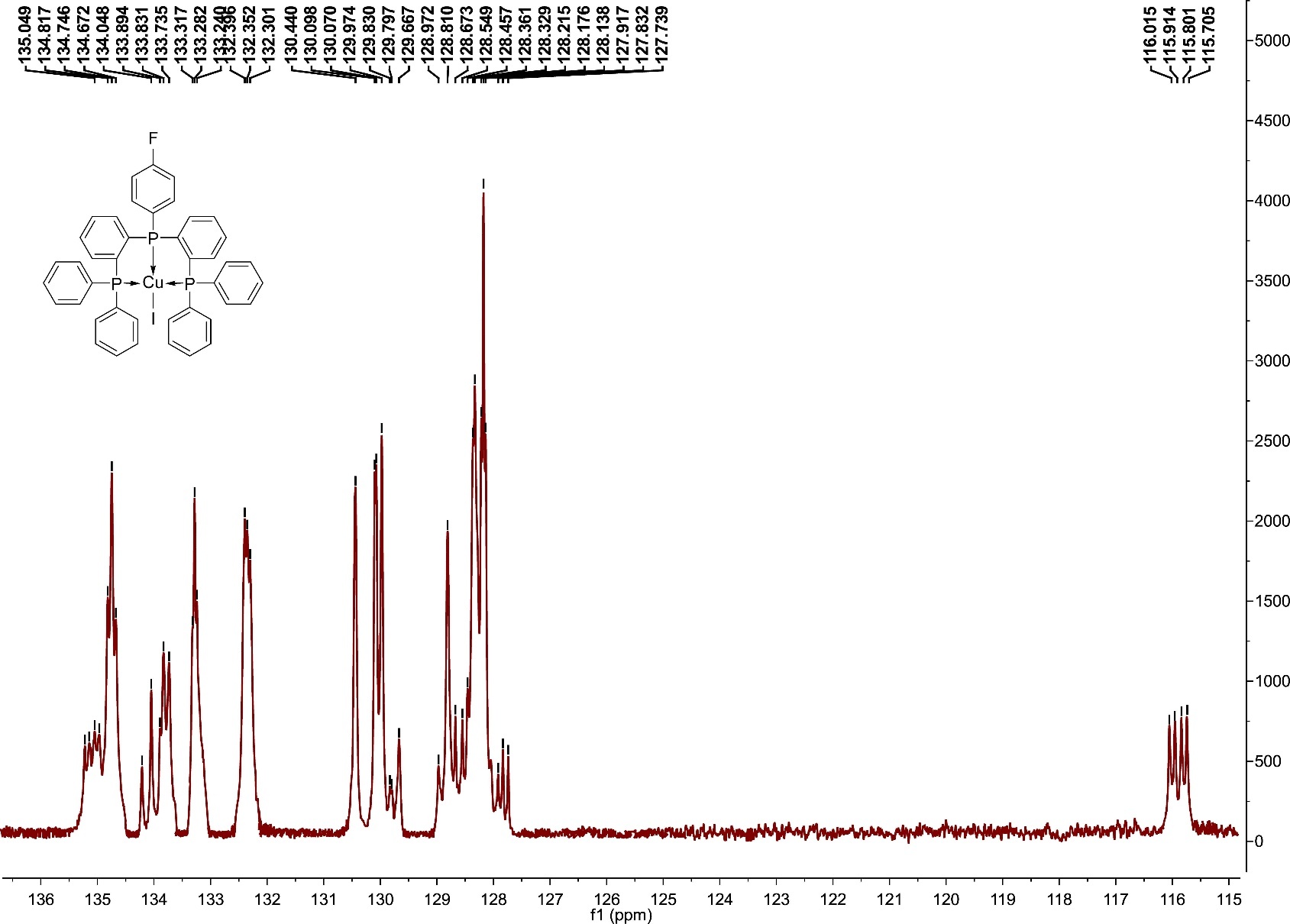


**Fig. S13.** ^13^C NMR (100 MHz) spectrum of **FTTPPCuI** in CDCl_3_.


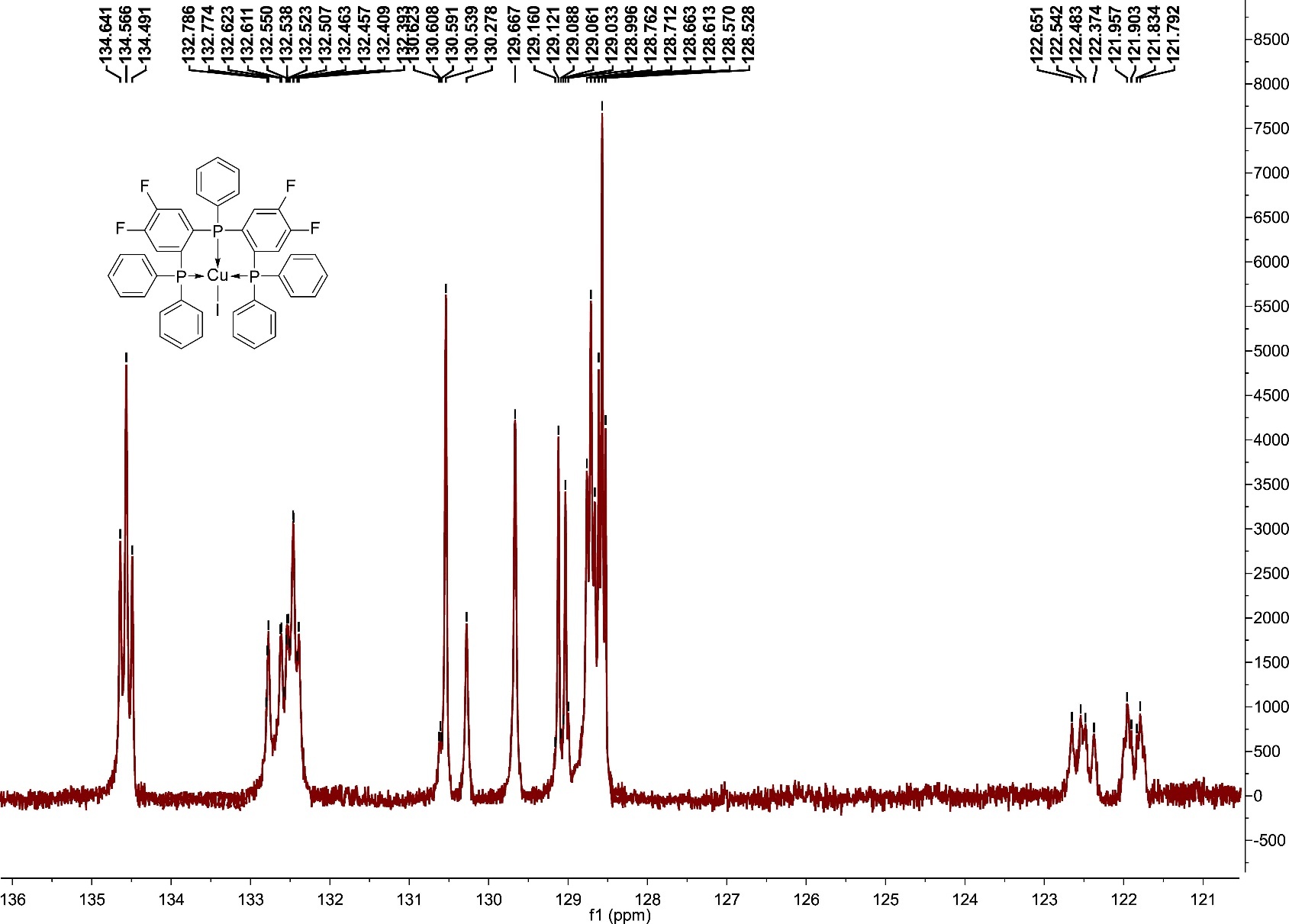


**Fig. S14.** ^13^C NMR (100 MHz) spectrum of **4FTTPPCuI** in CDCl_3_.


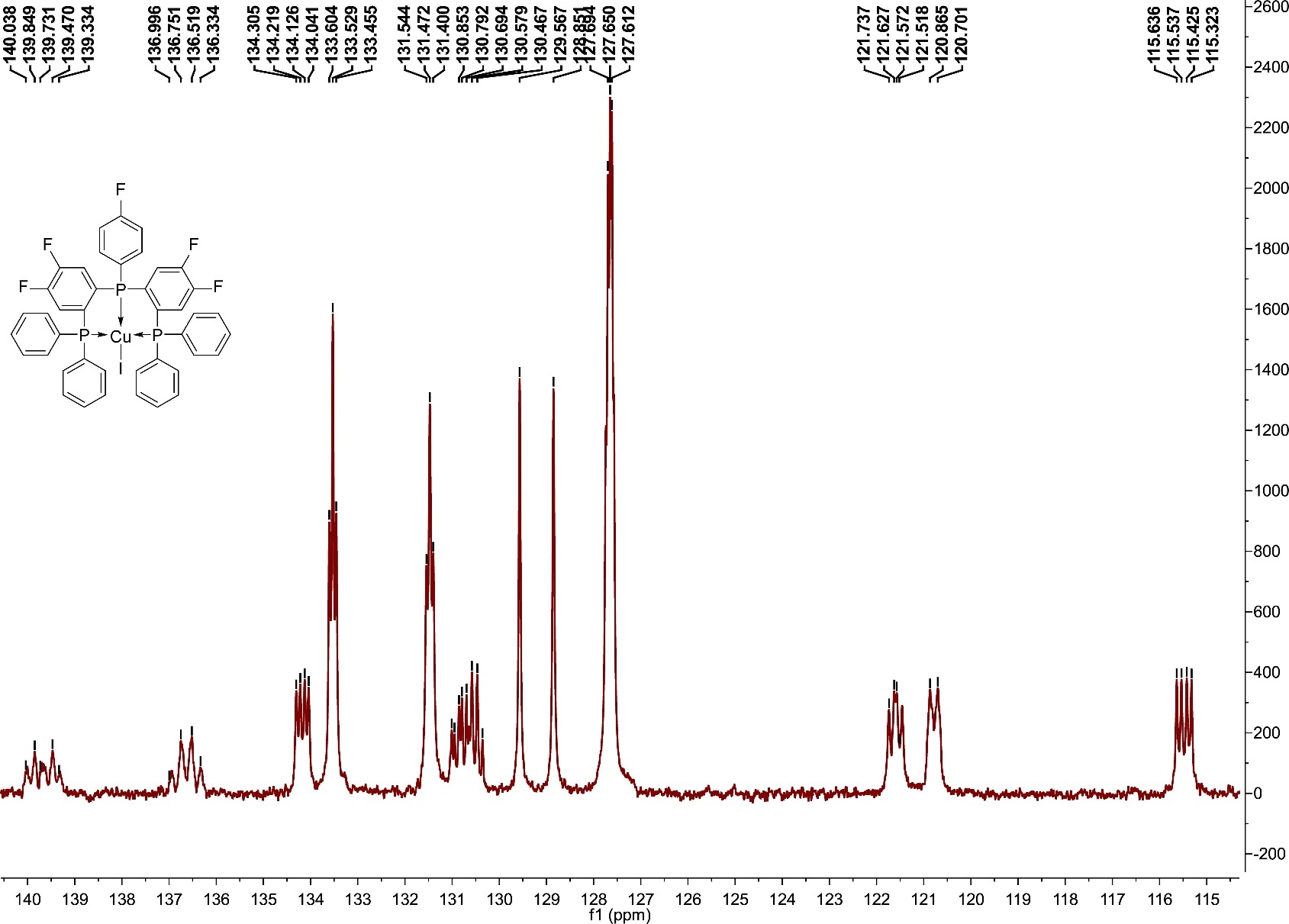


**Fig. S15.** ^13^C NMR (100 MHz) spectrum of **5FTTPPCuI** in CDCl_3_.


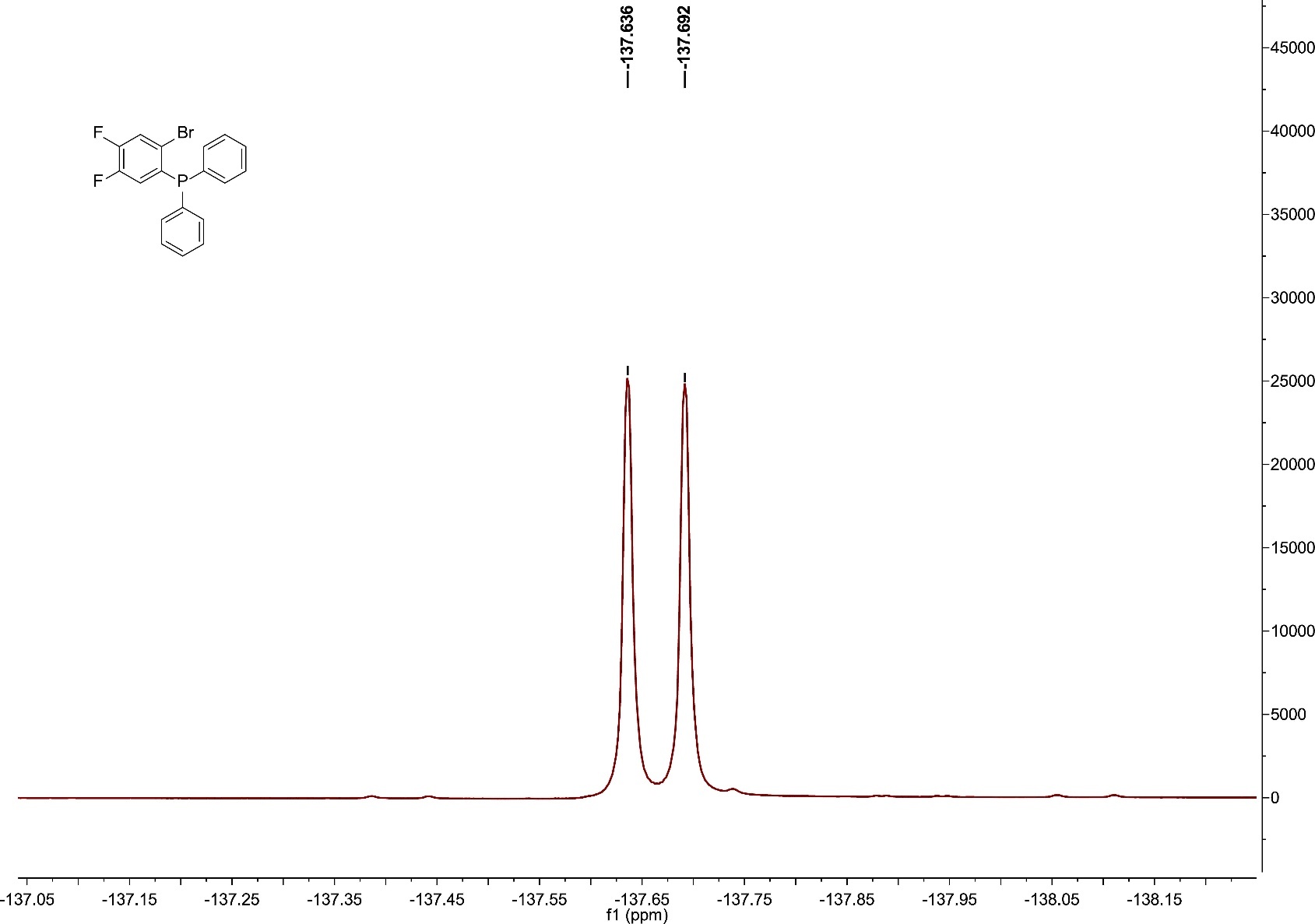


**Fig. S16.** ^19^F NMR (376 MHz) spectrum of **2FPhSPBr** in CDCl_3_.


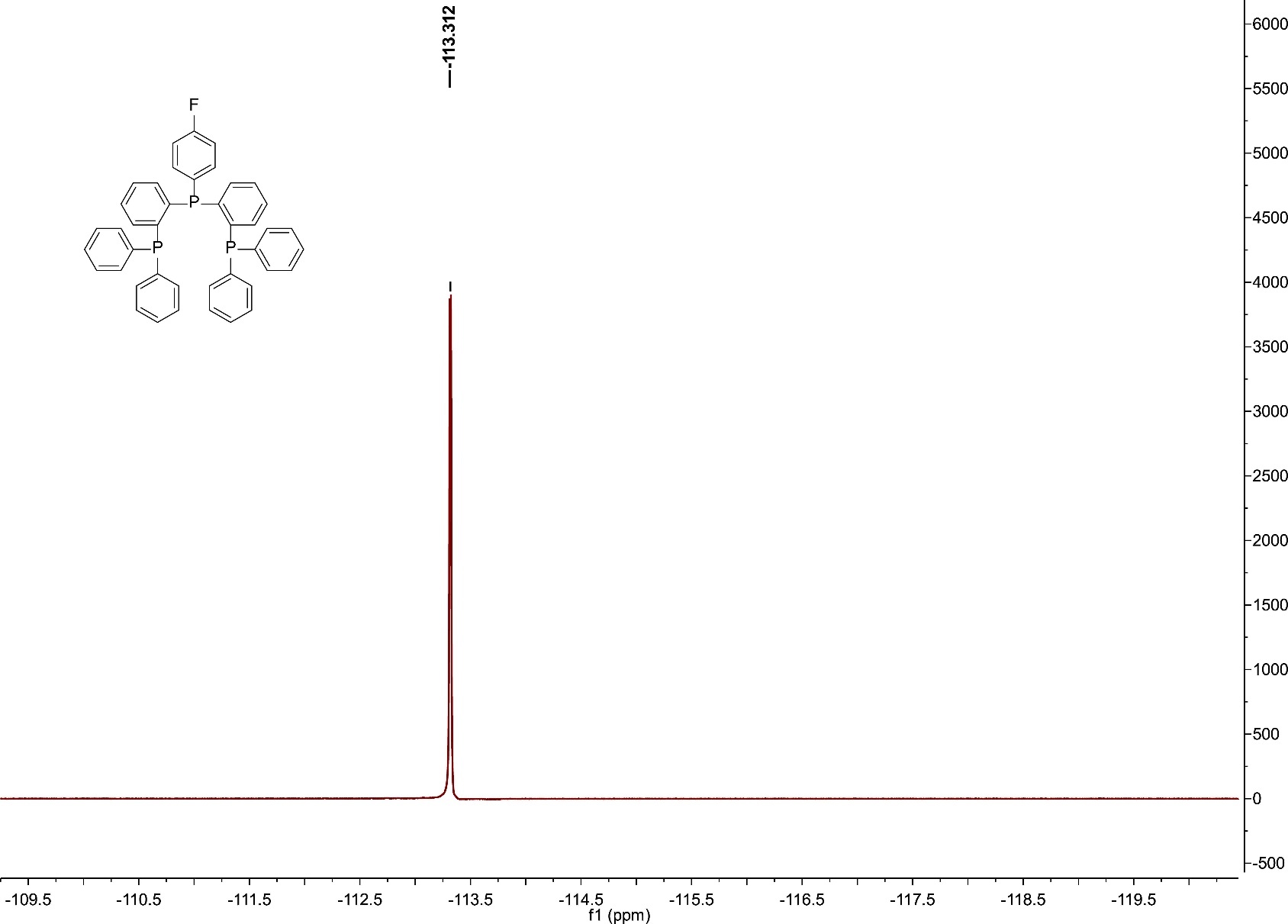


**Fig. S17.** ^19^F NMR (376 MHz) spectrum of **FTTPP** in CDCl_3_.


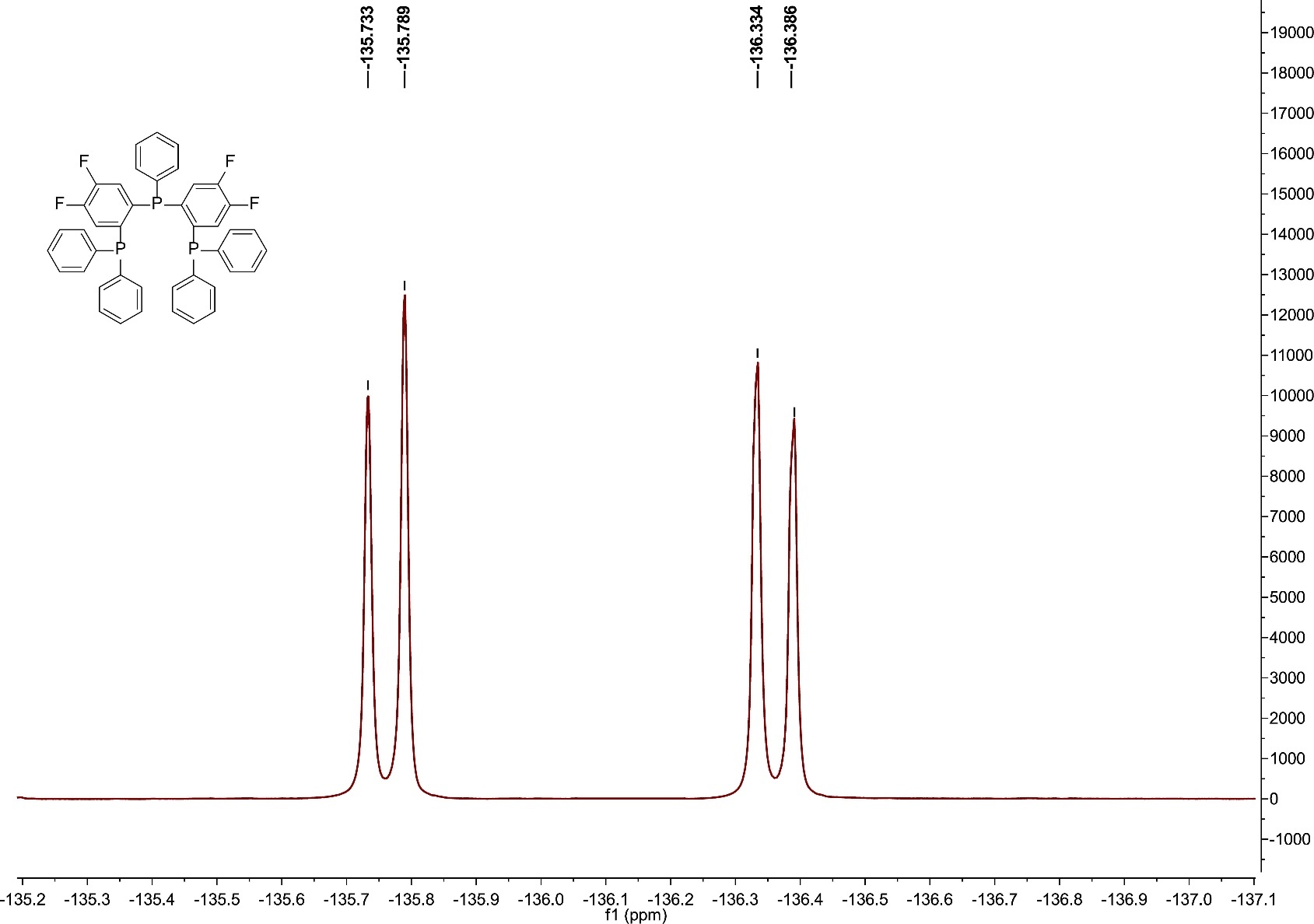


**Fig. S18.** ^19^F NMR (376 MHz) spectrum of **4FTTPP** in CDCl_3_.


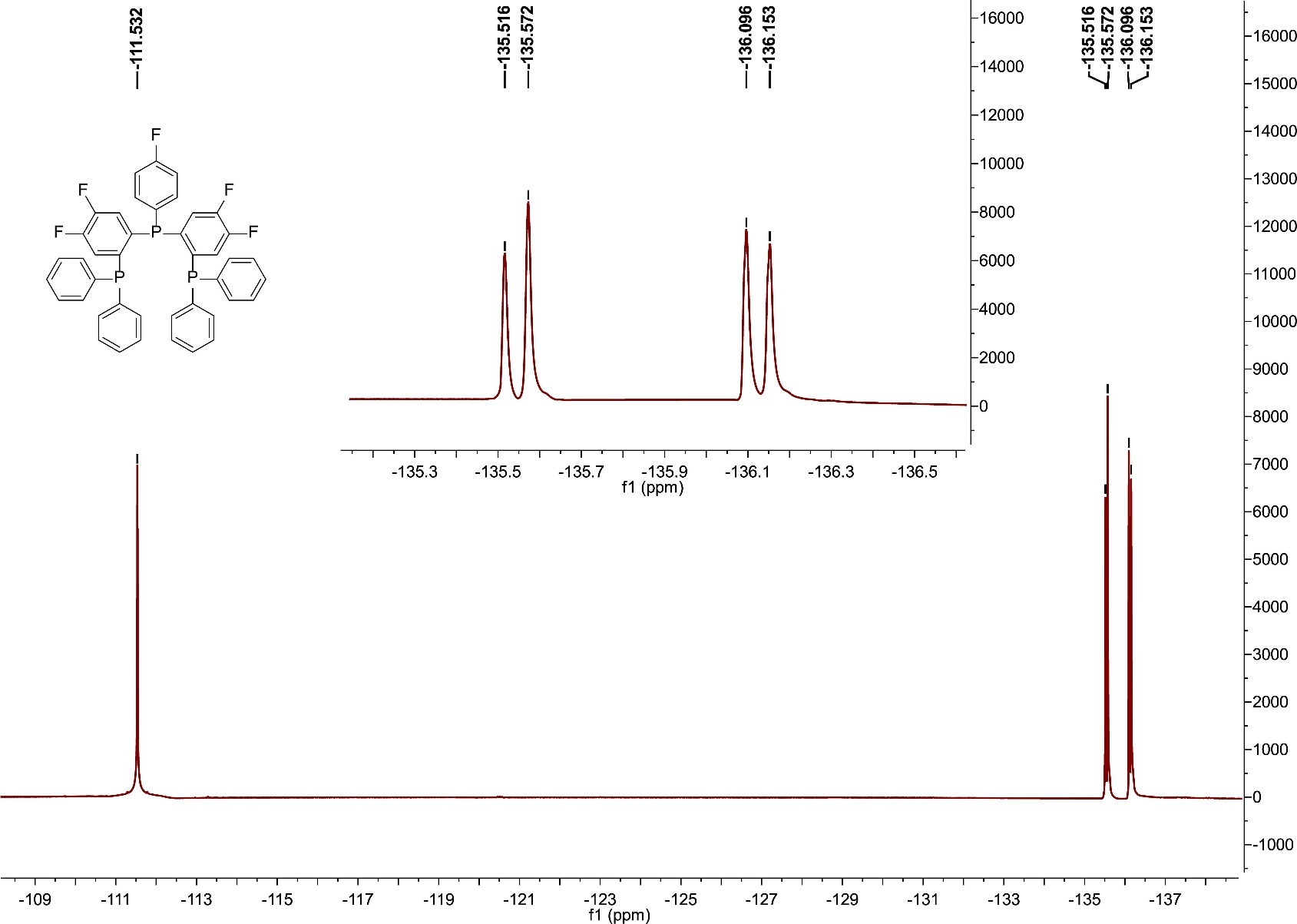


**Fig. S19.** ^19^F NMR (376 MHz) spectrum of **5FTTPP** in CDCl_3_.


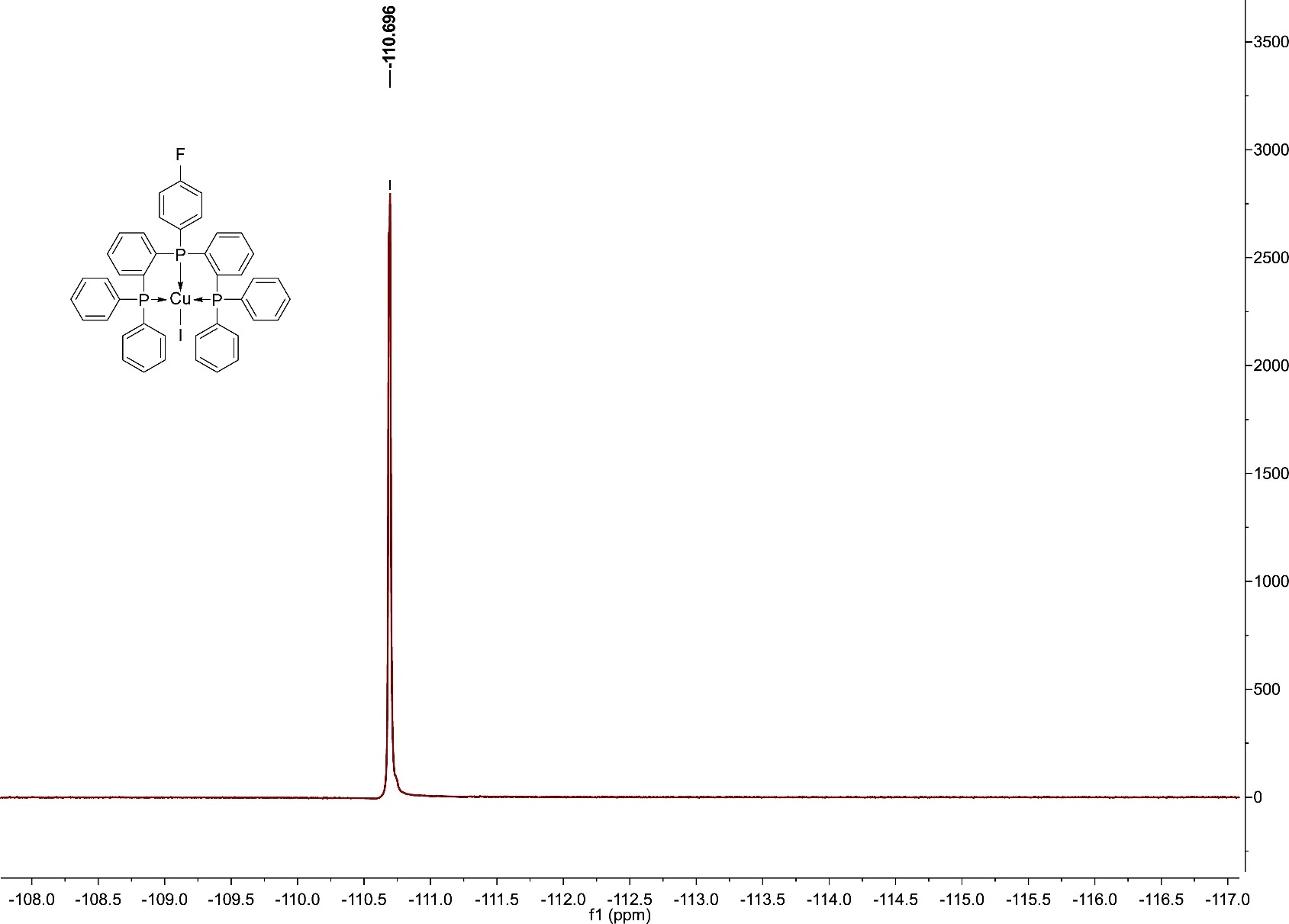


**Fig. S20.** ^19^F NMR (376 MHz) spectrum of **FTTPPCuI** in CDCl_3_.


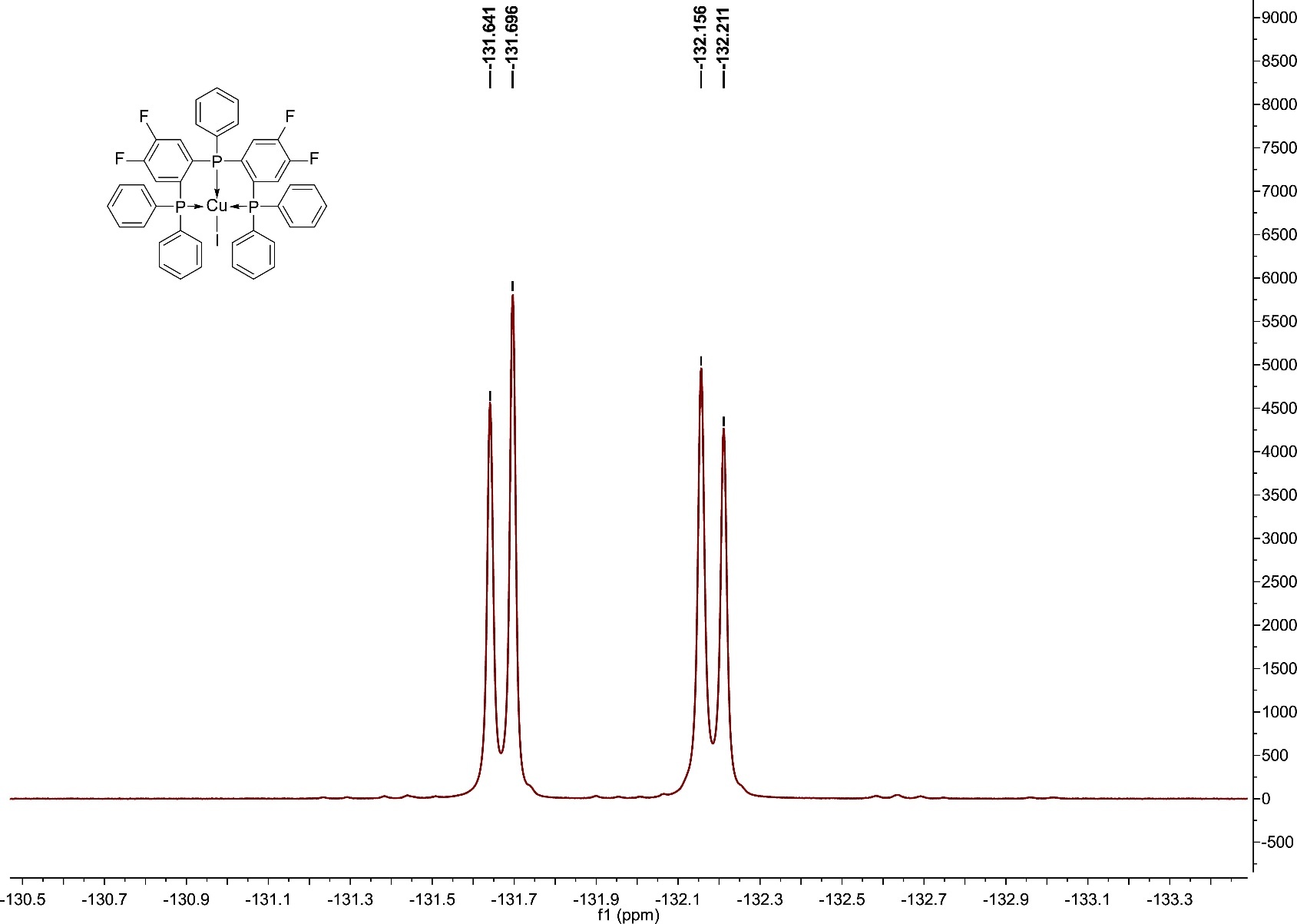


**Fig. S21.** ^19^F NMR (376 MHz) spectrum of **4FTTPPCuI** in CDCl_3_.


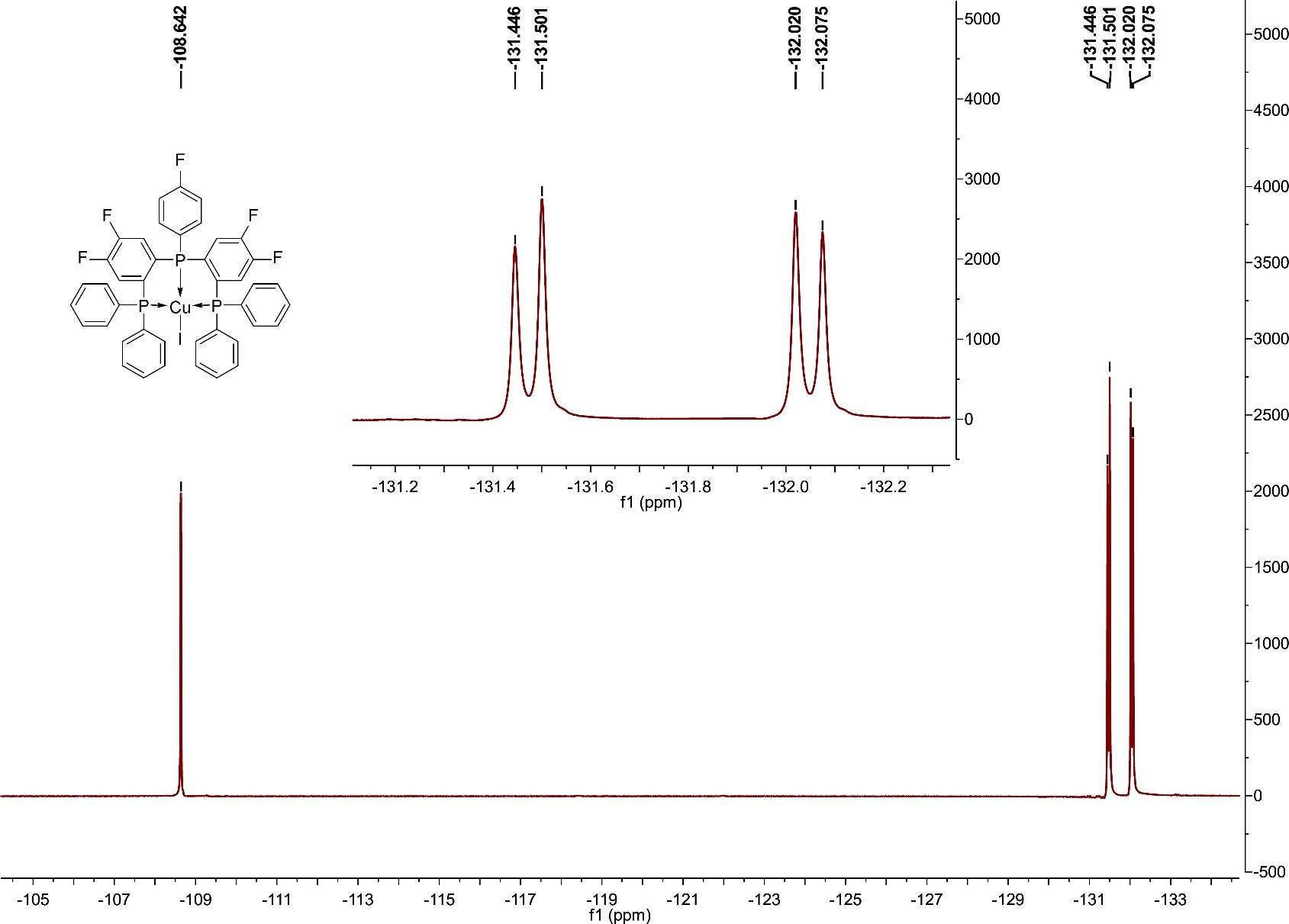


**Fig. S22.** ^19^F NMR (376 MHz) spectrum of **5FTTPPCuI** in CDCl_3_.

### Single Crystal Structures


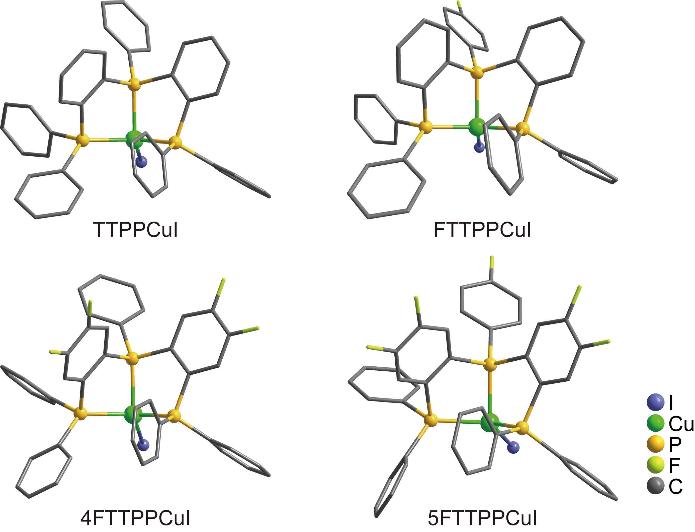


**Fig. S23.** Single crystal structures of **TTPPCuI**, **FTTPPCuI**, **4FTTPPCuI**, and **5FTTPPCuI**.


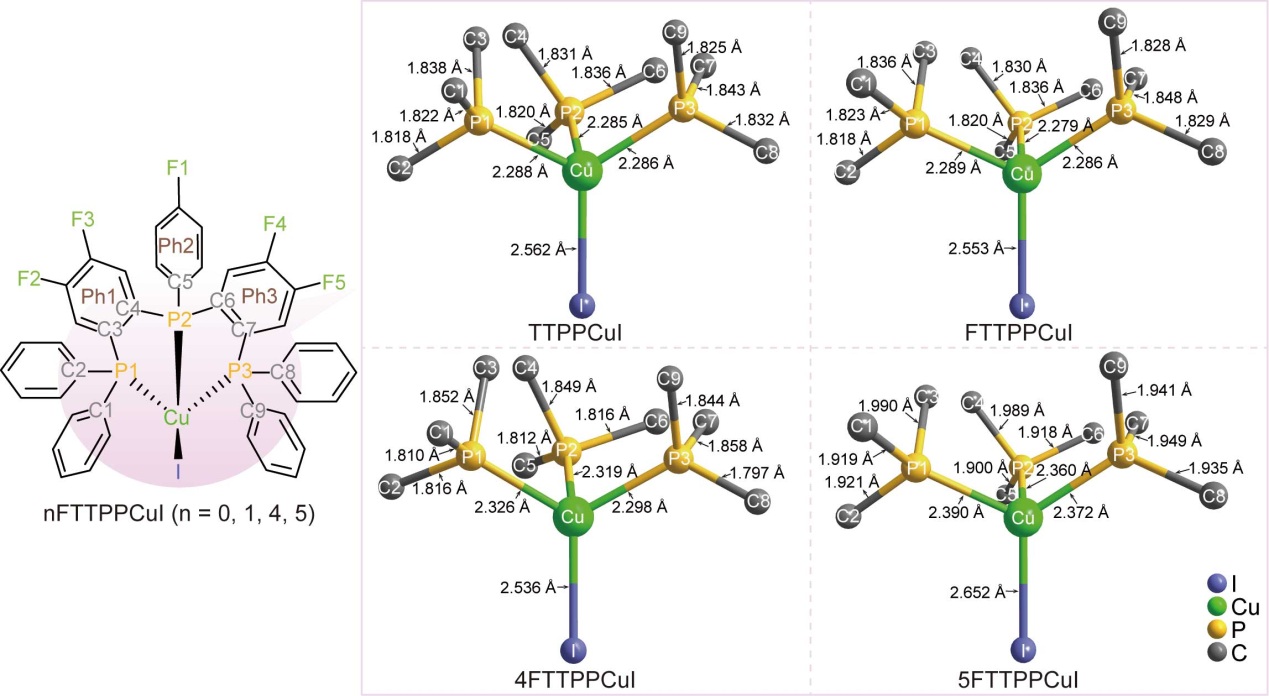


**Fig. S24.** Chemical structures of **TTPPCuI**, **FTTPPCuI**, **4FTTPPCuI**, and **5FTTPPCuI**. The bond lengths of complex cores are highlighted.


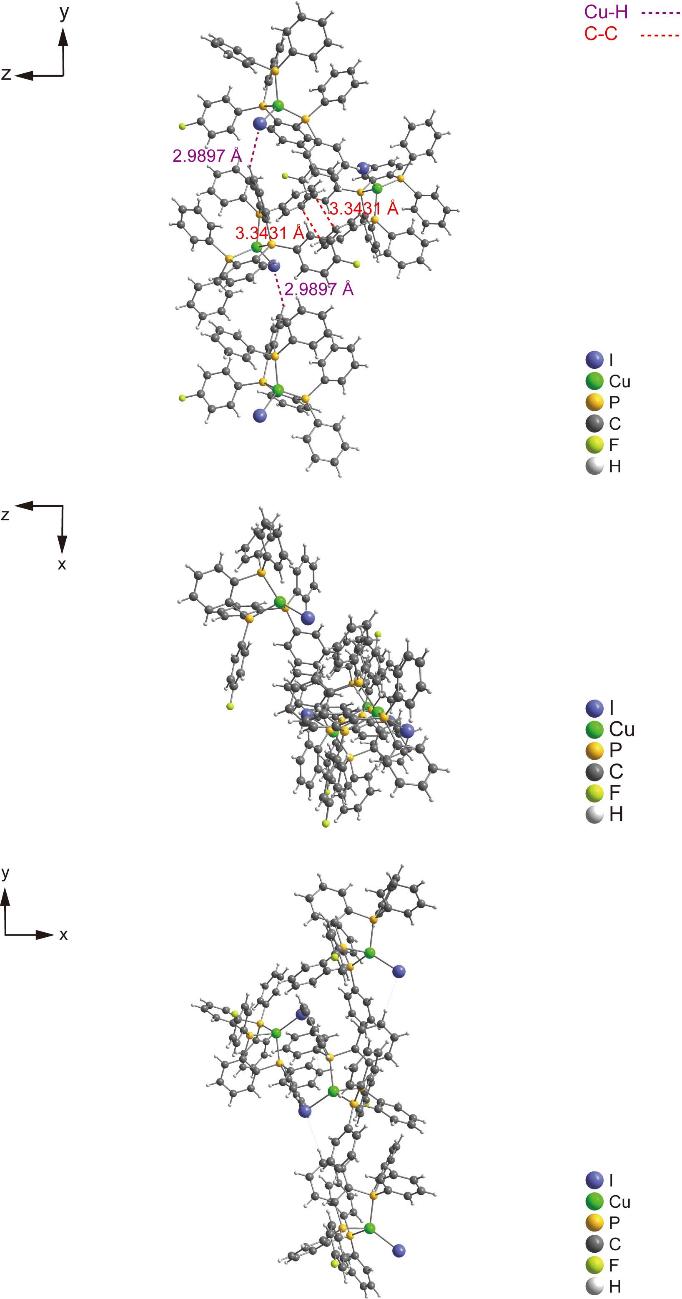


**Fig. S25.** Single-crystal packing diagrams of **FTTPPCuI** viewed along x, y, and z axes, respectively.

**
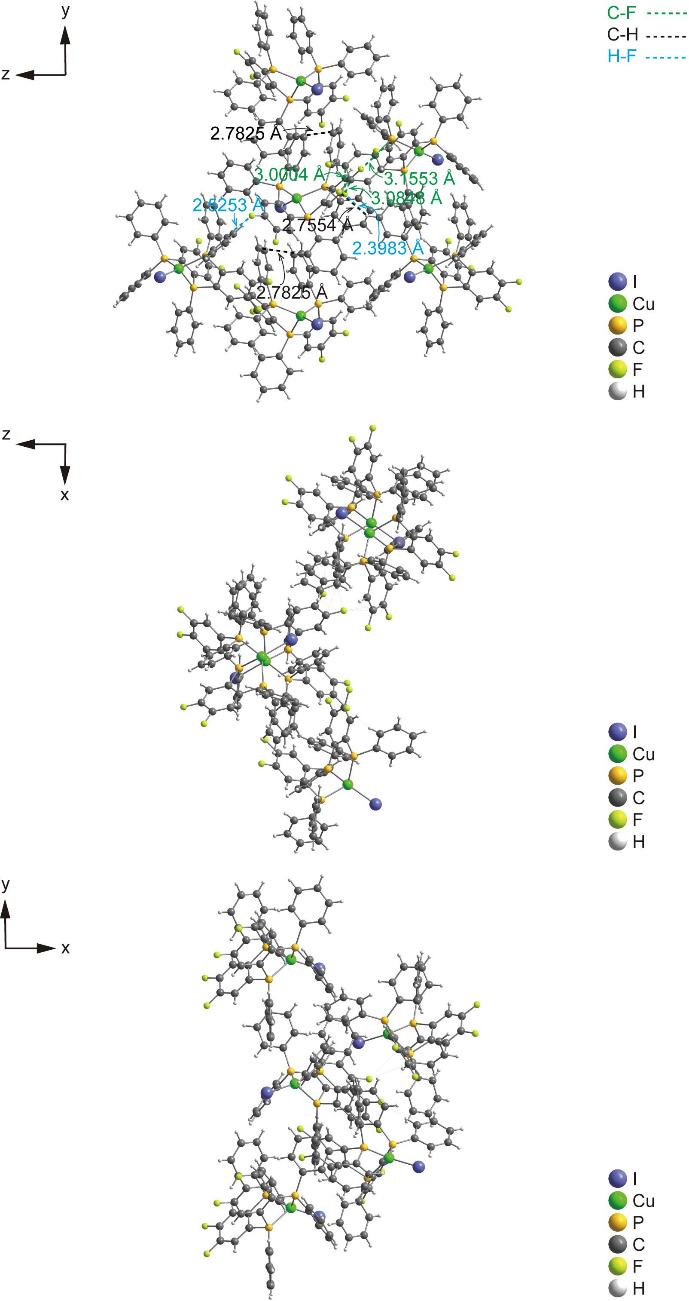
**

**Fig. S26.** Single-crystal packing diagrams of **4FTTPPCuI** viewed along x, y, and z axes, respectively.


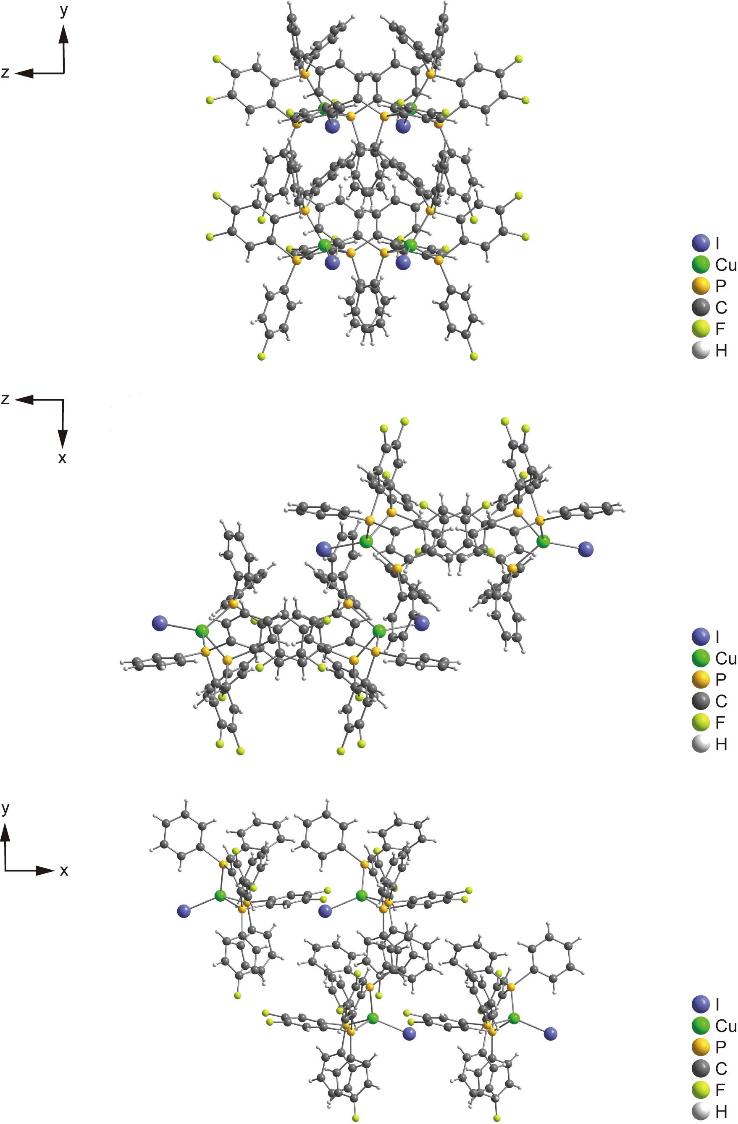


**Fig. S27.** Single-crystal packing diagrams of **5FTTPPCuI** viewed along x, y, and z axes, respectively.

### Table S1. The selected bond lengths (Å) of TTPPCuI single crystal.

| **Cu-I** | 2.562 | **P2-C4** | 1.831 |
| --- | --- | --- | --- |
| **Cu-P1** | 2.288 | **P2-C5** | 1.820 |
| **Cu-P2** | 2.285 | **P2-C6** | 1.836 |
| **Cu-P3** | 2.286 | **P3-C7** | 1.843 |
| **P1-C1** | 1.822 | **P3-C8** | 1.832 |
| **P1-C2** | 1.818 | **P3-C9** | 1.825 |
| **P1-C3** | 1.838 |  |  |

### Table S2. The selected bond lengths (Å) of FTTPPCuI single crystal.

| **Cu-I** | 2.553 | **P2-C4** | 1.830 |
| --- | --- | --- | --- |
| **Cu-P1** | 2.289 | **P2-C5** | 1.820 |
| **Cu-P2** | 2.279 | **P2-C6** | 1.836 |
| **Cu-P3** | 2.286 | **P3-C7** | 1.848 |
| **P1-C1** | 1.823 | **P3-C8** | 1.829 |
| **P1-C2** | 1.818 | **P3-C9** | 1.828 |
| **P1-C3** | 1.836 |  |  |

### Table S3. The selected bond lengths (Å) of 4FTTPPCuI single crystal.

| **Cu-I** | 2.536 | **P2-C4** | 1.849 |
| --- | --- | --- | --- |
| **Cu-P1** | 2.326 | **P2-C5** | 1.812 |
| **Cu-P2** | 2.319 | **P2-C6** | 1.816 |
| **Cu-P3** | 2.298 | **P3-C7** | 1.858 |
| **P1-C1** | 1.810 | **P3-C8** | 1.797 |
| **P1-C2** | 1.816 | **P3-C9** | 1.844 |
| **P1-C3** | 1.852 |  |  |

### Table S4. The selected bond lengths (Å) of 5FTTPPCuI single crystal.

| **Cu-I** | 2.652 | **P2-C4** | 1.989 |
| --- | --- | --- | --- |
| **Cu-P1** | 2.390 | **P2-C5** | 1.900 |
| **Cu-P2** | 2.360 | **P2-C6** | 1.918 |
| **Cu-P3** | 2.372 | **P3-C7** | 1.949 |
| **P1-C1** | 1.919 | **P3-C8** | 1.935 |
| **P1-C2** | 1.921 | **P3-C9** | 1.941 |
| **P1-C3** | 1.990 |  |  |

### Thermal Properties


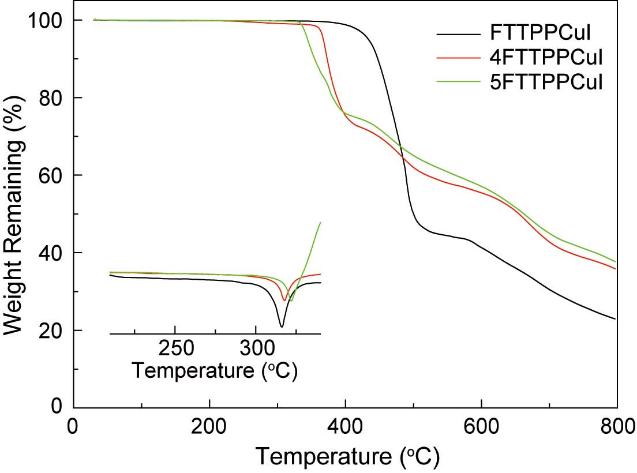


**Fig. S28.** Thermogravimetric analysis (TGA) and differential scanning calorimetry (DSC) curves of **FTTPPCuI**, **4FTTPPCuI**, and **5FTTPPCuI** powders.

### DFT and TDDFT Simulations


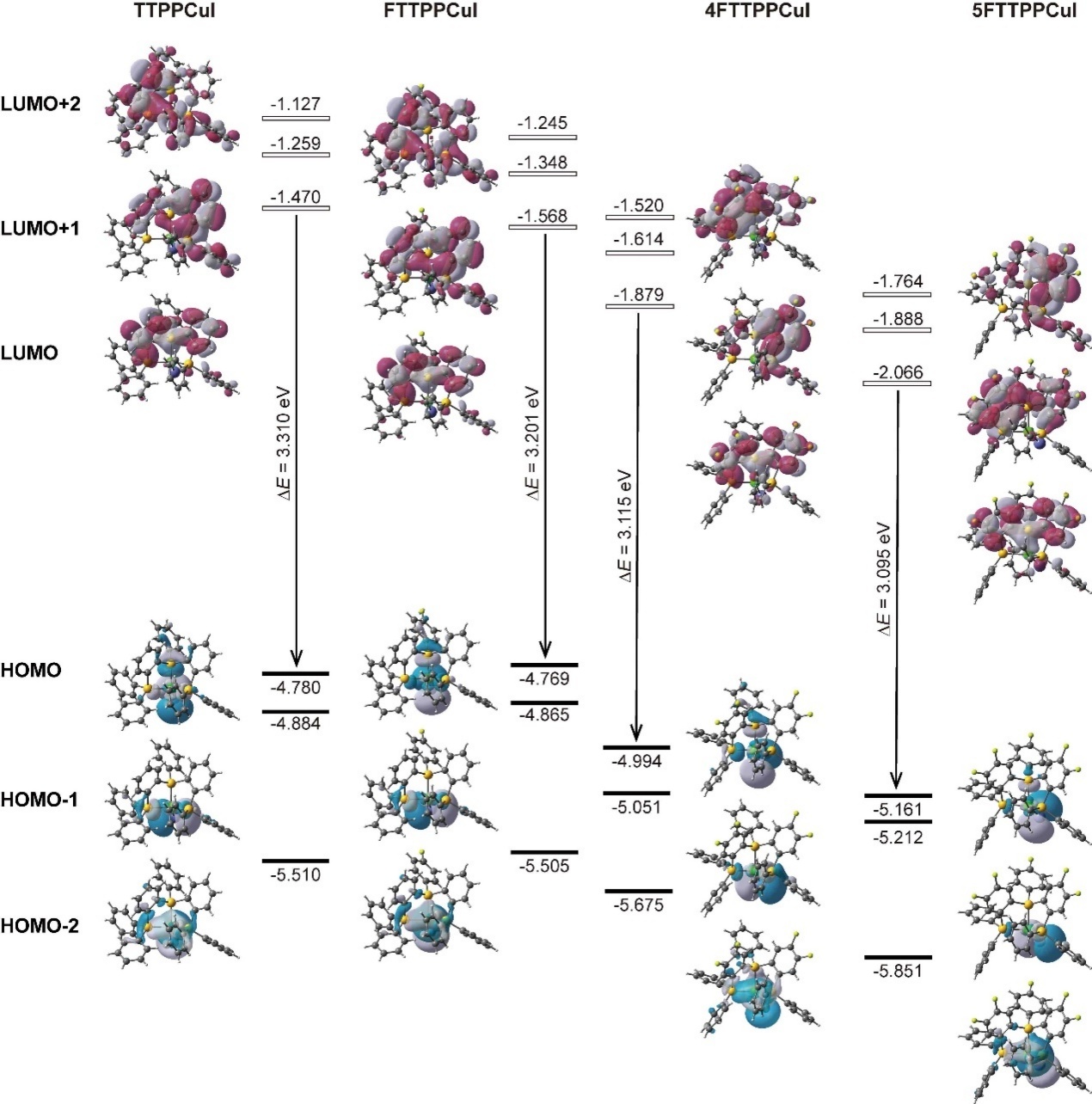


**Fig.** **S29.** Energy levels and contours of frontier molecular orbitals (FMO) for **TTPPCuI**, **FTTPPCuI**, **4FTTPPCuI**, and **5FTTPPCuI** simulated at the level of B3LYP/6-31G*.


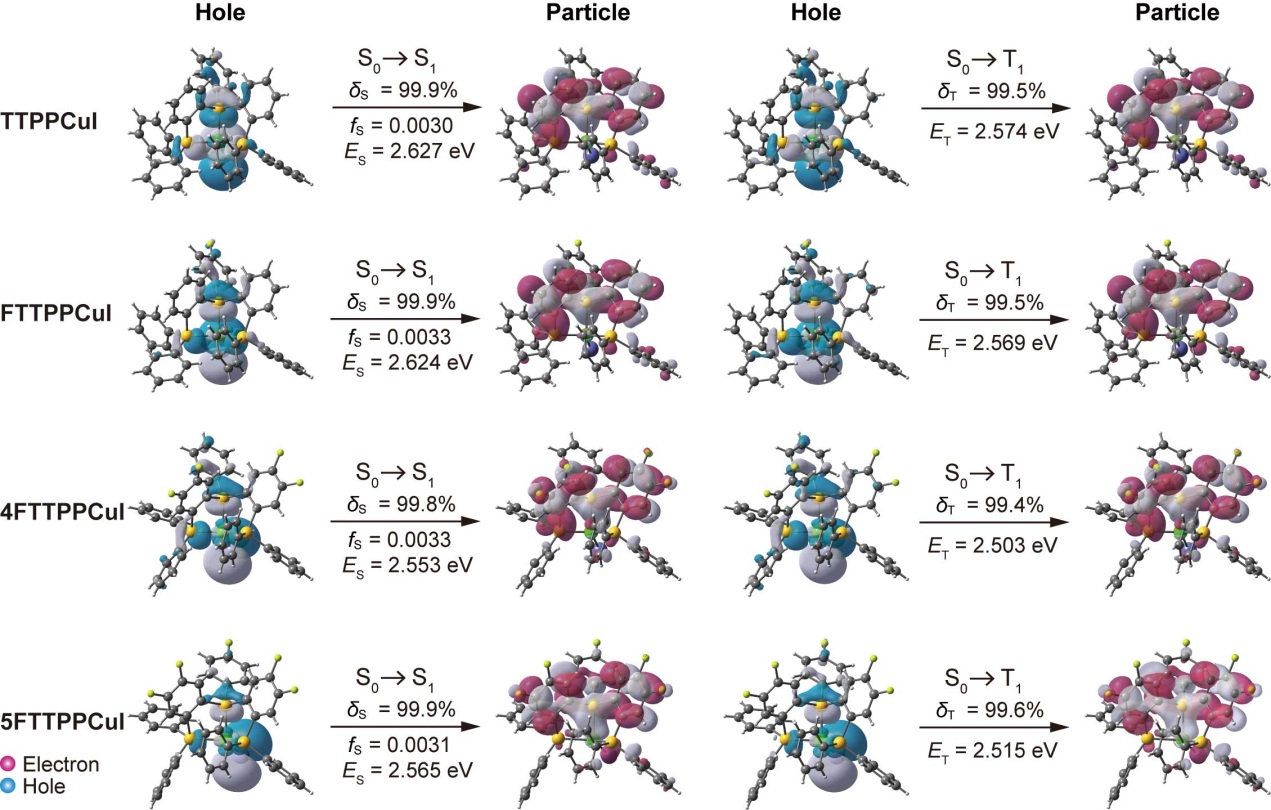


**Fig.** **S30.** Contours and key transition parameters of the S_0_→S_1_ and S_0_→T_1_ excitations for **TTPPCuI**, **FTTPPCuI**, **4FTTPPCuI**, and **5FTTPPCuI** calculated with natural transition orbital (NTO) approach. *δ*, *f* and *E* refer to contribution weight, oscillator strength, and excited-state energy level. Subscripts of “S” and “T” represent “singlet” and “triplet”, respectively.


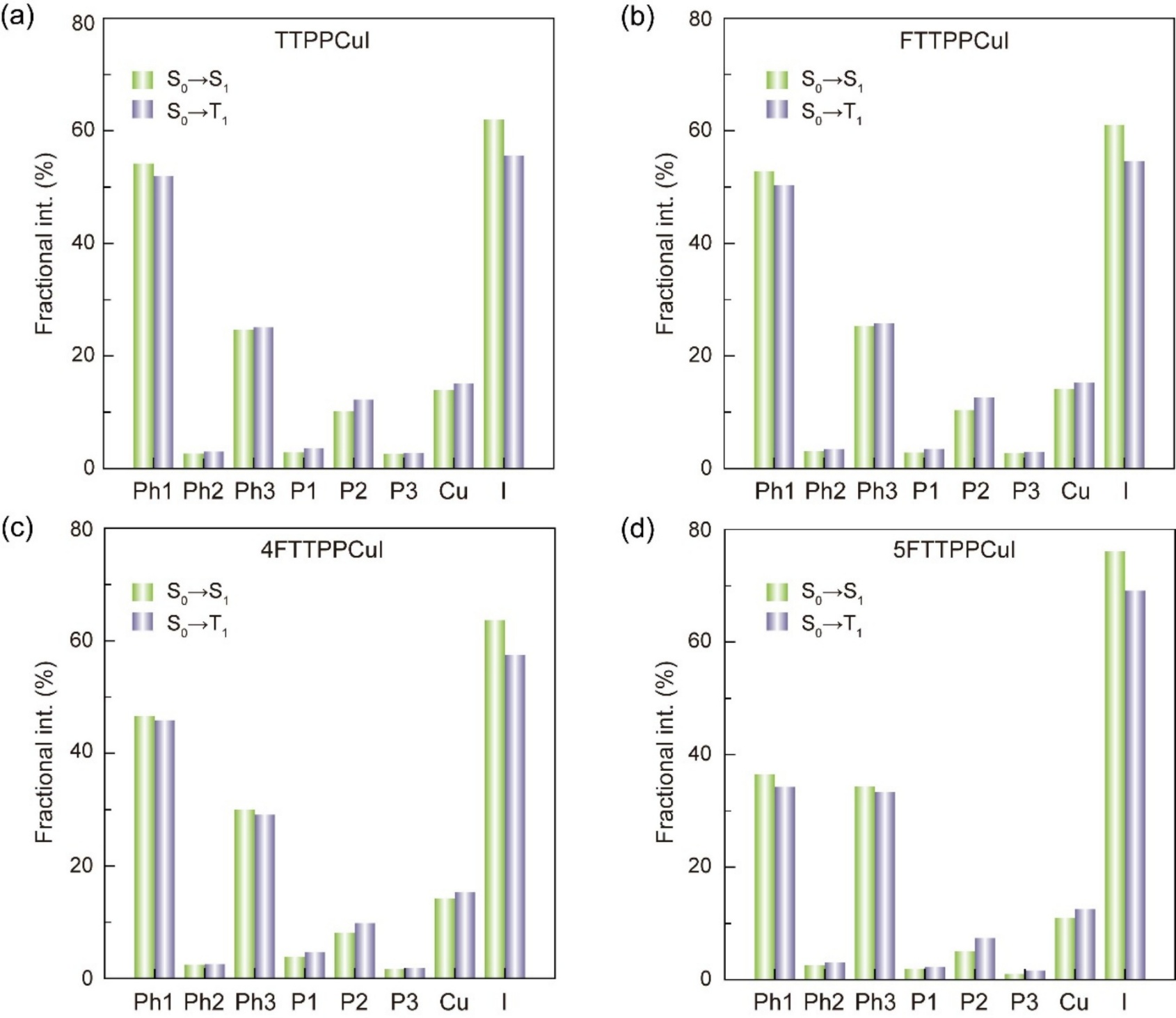


**Fig.** **S31.** Contribution percentage of three phenyl rings (Ph1, Ph2, and Ph3) to the electron and representative atoms (P1, P2, P3, Cu, and I) to the hole in **nFTTPPCuI** complexes during the S_0_→S_1_ and S_0_→T_1_ transitions. (a) **TTPPCuI.** (b) **FTTPPCuI.** (c) **4FTTPPCuI.** (d) **5FTTPPCuI.**

### Table S5. Fractional intensities of representative atoms of TTPPCuI, FTTPPCuI, 4FTTPPCuI, and 5FTTPPCuI to FMO.

| Cu(I) complexes | energy level | Ph1 | Ph2 | Ph3 | P1 | P2 | P3 | Cu | I |
| --- | --- | --- | --- | --- | --- | --- | --- | --- | --- |
| TTPPCuI | LUMO | 53.92% | 2.55% | 25.49% | 0.87% | 6.49% | 0.91% | 0.92% | 0.12% |
|  | HOMO | - | - | - | 1.80% | 7.22% | 2.28% | 10.62% | 71.32% |
| FTTPPCuI | LUMO | 53.37% | 2.96% | 25.92% | 0.81% | 6.54% | 0.94% | 0.94% | 0.13% |
|  | HOMO | - | - | - | 1.40% | 6.60% | 2.24% | 10.86% | 72.54% |
| 4FTTPPCuI | LUMO | 33.88% | 2.27% | 29.11% | 1.14% | 5.20% | 1.44% | 1.54% | 0.27% |
|  | HOMO | - | - | - | 2.85% | 5.25% | 0.81% | 11.19% | 74.02% |
| 5FTTPPCuI | LUMO | 40.47% | 2.38% | 34.17% | 0.56% | 9.68% | 2.44% | 1.51% | 0.48% |
|  | HOMO | - | - | - | 1.38% | 3.12% | 0.53% | 8.79% | 82.93% |

### Table S6. Fractional intensities of representative atoms of TTPPCuI, FTTPPCuI, 4FTTPPCuI, and 5FTTPPCuI to NTO of S_0_→S_1_.

| Cu(I) complexes | S_0_→S_1_ | | Ph1 | | Ph2 | Ph3 | P1 | P2 | P3 | Cu | I |
| --- | --- | --- | --- | --- | --- | --- | --- | --- | --- | --- | --- |
| TTPPCuI | | Electron | 54.17% | 2.64% | | 24.66% | 0.90% | 7.50% | 1.06% | 1.12% | 0.15% |
|  |  | Hole | - | - | | - | 2.86% | 10.08% | 2.58% | 13.90% | 61.96% |
| FTTPPCuI | | Electron | 52.82% | 3.02% | | 25.35% | 0.81% | 7.75% | 1.14% | 1.15% | 0.17% |
|  |  | Hole | - | - | | - | 2.82% | 10.36% | 2.70% | 14.10% | 61.06% |
| 4FTTPPCuI | | Electron | 46.65% | 2.43% | | 30.01% | 1.32% | 6.25% | 1.60% | 1.84% | 0.31% |
|  |  | Hole | - | - | | - | 3.84% | 8.14% | 1.64% | 14.22% | 63.67% |
| 5FTTPPCuI | | Electron | 36.5% | 2.56% | | 34.31% | 0.78% | 11.82% | 2.95% | 2.04% | 0.66% |
|  |  | Hole | - | - | | - | 1.94% | 5.10% | 1.01% | 11.00% | 76.09% |

### Table S7. Fractional intensities of representative atoms of TTPPCuI, FTTPPCuI, 4FTTPPCuI, and 5FTTPPCuI to NTO of S_0_→T_1_.

| Cu(I) complexes | S_0_→T_1_ | Ph1 | Ph2 | Ph3 | P1 | P2 | P3 | Cu | I |
| --- | --- | --- | --- | --- | --- | --- | --- | --- | --- |
| TTPPCuI | Electron | 51.91% | 3.01% | 25.07% | 0.77% | 8.96% | 1.27% | 1.41% | 0.21% |
|  | Hole | - | - | - | 3.59% | 12.22% | 2.78% | 15.07% | 55.63% |
| FTTPPCuI | Electron | 50.33% | 3.44% | 25.76% | 0.67% | 9.30% | 1.39% | 1.47% | 0.23% |
|  | Hole | - | - | - | 3.51% | 12.57% | 2.93% | 15.26% | 54.54% |
| 4FTTPPCuI | Electron | 45.89% | 2.58% | 29.11% | 1.49% | 7.41% | 1.53% | 2.12% | 0.33% |
|  | Hole | - | - | - | 4.70% | 9.82% | 1.87% | 15.31% | 57.52% |
| 5FTTPPCuI | Electron | 34.26% | 3.11% | 33.27% | 1.00% | 13.6% | 3.16% | 2.50% | 0.81% |
|  | Hole | - | - | - | 2.30% | 7.1% | 1.64% | 12.53% | 69.15% |

### Electrochemical Properties

**
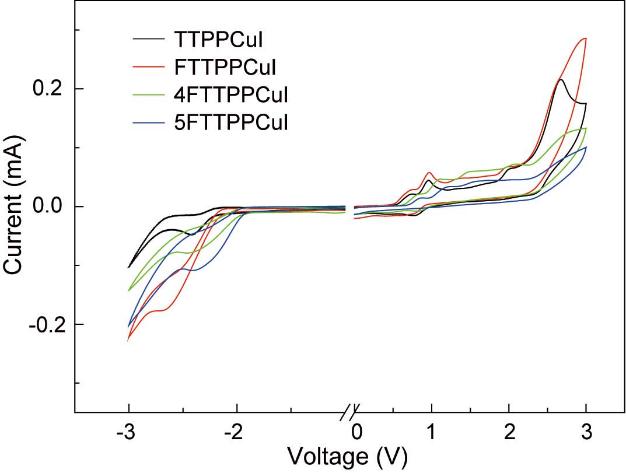
**

**Fig.** **S32.** CV of **TTPPCuI**, **FTTPPCuI**, **4FTTPPCuI**, and **5FTTPPCuI** measured at room temperature with the scanning rate of 100 mV s^-1^.

### Photophysical Properties


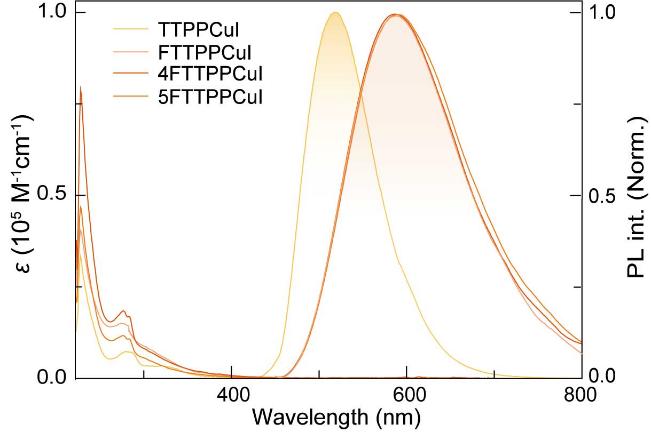


**Fig.** **S33.** Electronic absorption spectra in dilute DCM solution (10^-6^ mol L^-1^) and PL spectra of neat films for **nFTTPPCuI** at room temperature. Excitation wavelength is 350 nm. Emission peak wavelengths of **TTPPCuI**, **FTTPPCuI**, **4FTTPPCuI**, and **5FTTPPCuI** are 521, 588, 589, and 590 nm, respectively.


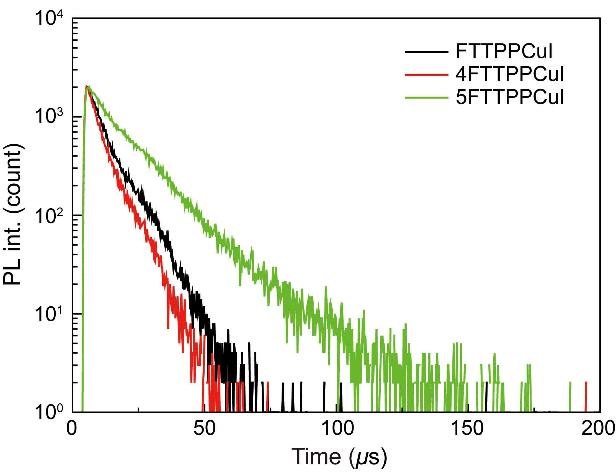


**Fig.** **S34.** Time decay curves of **nFTTPPCuI** neat films at room temperature. For **FTTPPCuI**, **4FTTPPCuI**, and **5FTTPPCuI**, emission peak wavelengths are chosen as 588, 589, and 590 nm, respectively, and excitation wavelength is 350 nm.


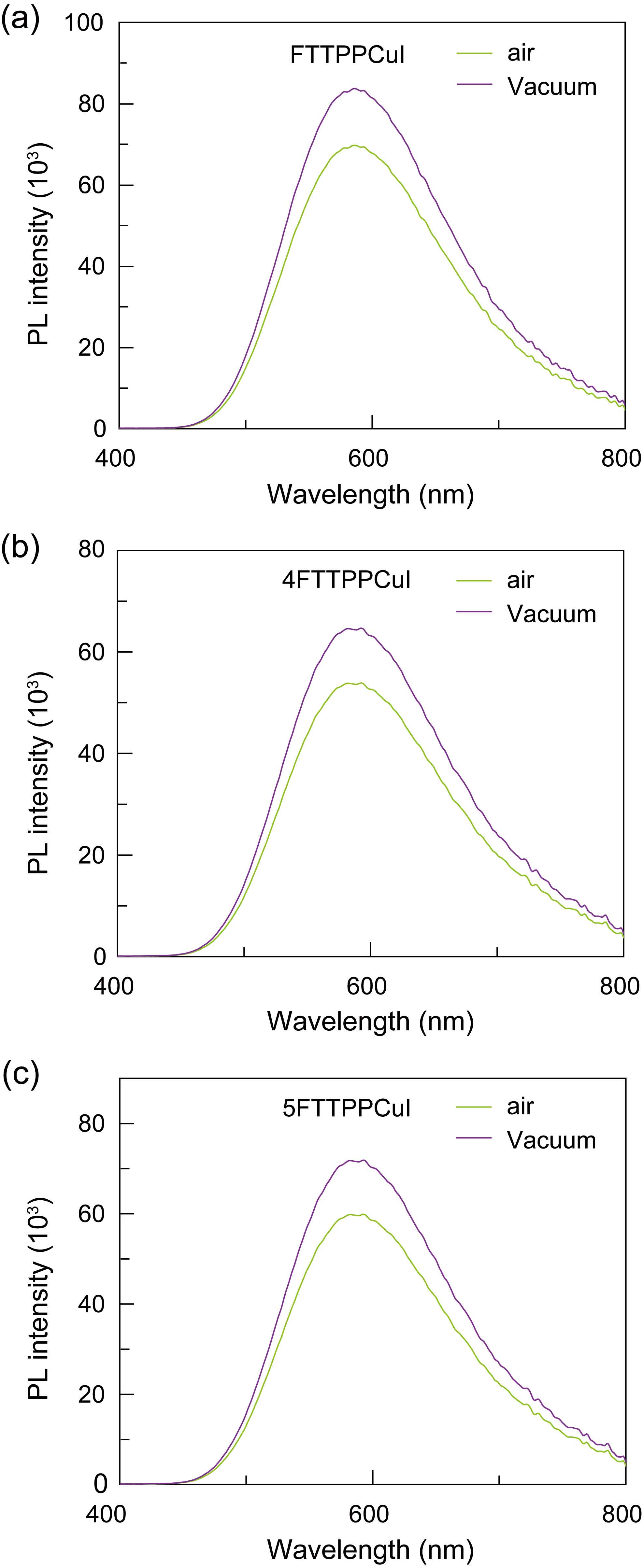


**Fig.** **S35.** PL spectra of neat films for **nFTTPPCuI** under air and vacuum at room temperature. For **FTTPPCuI**, **4FTTPPCuI**, and **5FTTPPCuI**, emission peak wavelengths are chosen as 588, 589, and 590 nm, respectively, and excitation wavelength is 350 nm. (a) **FTTPPCuI.** (b) **4FTTPPCuI.** (c) **5FTTPPCuI.**

**
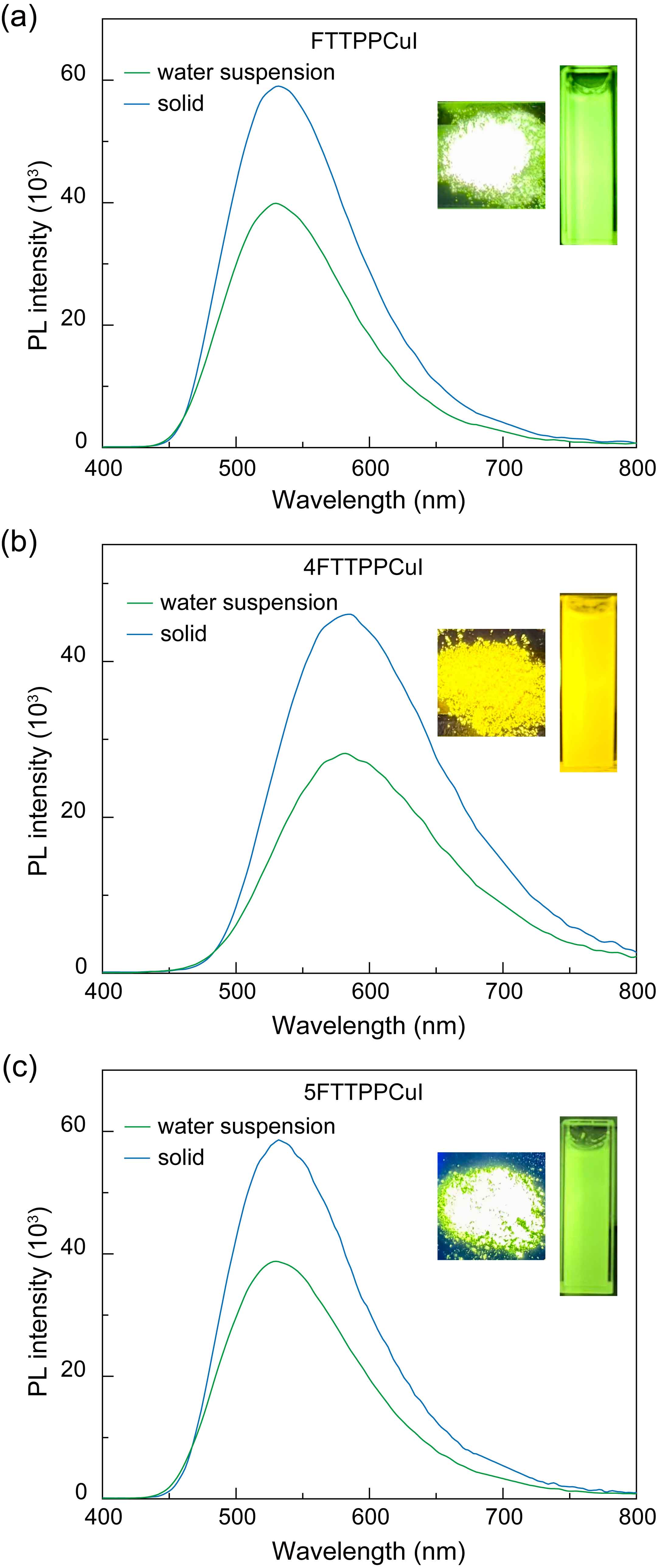
**

**Fig.** **S36.** PL spectra of **nFTTPPCuI** in the solid state and water suspension. (a) **FTTPPCuI.** (b) **4FTTPPCuI.** (c) **5FTTPPCuI.**


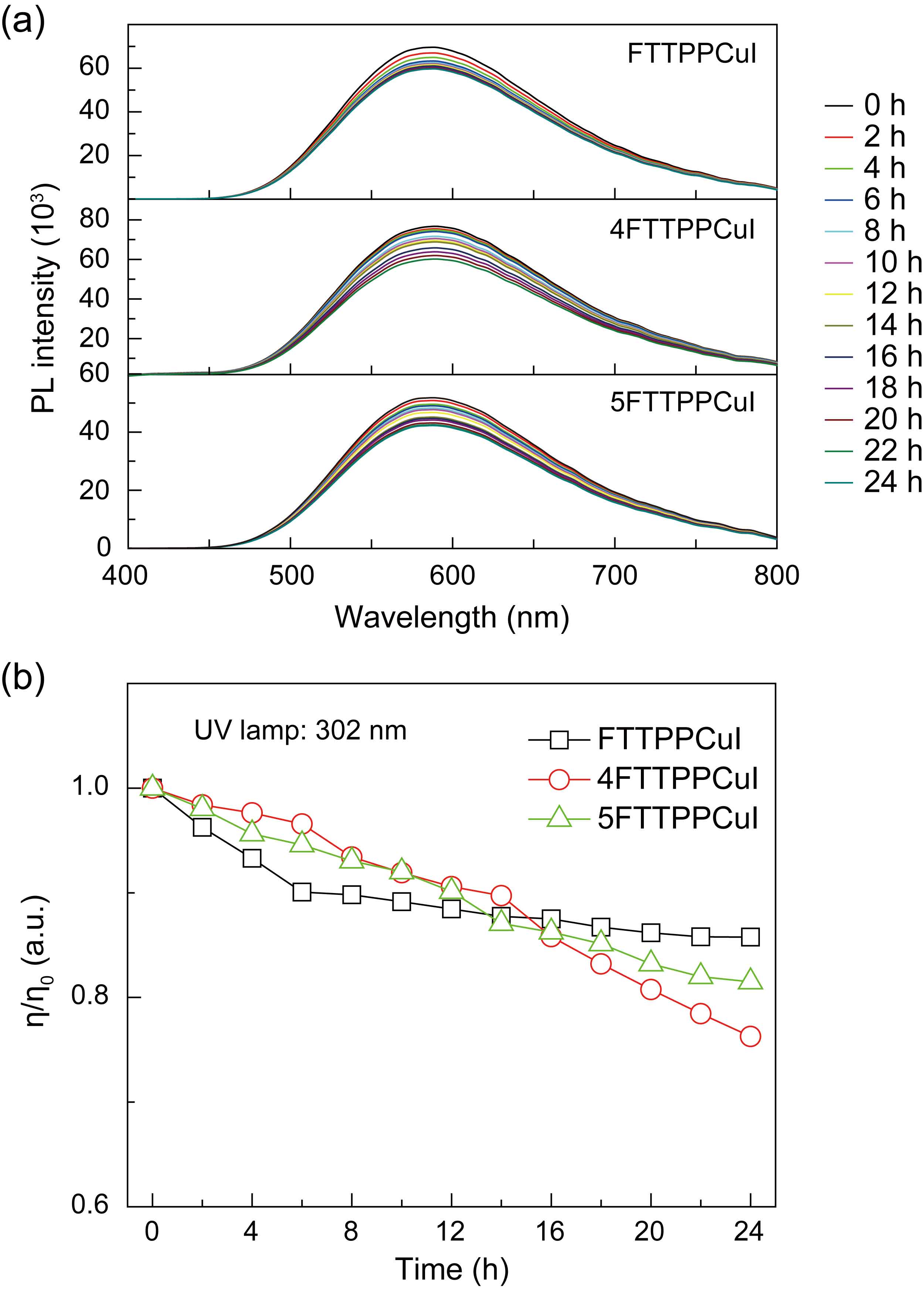


**Fig.** **S37.** Photostability test of **nFTTPPCuI**. (a) PL spectra are recorded every 2 h under ultraviolet (UV) exposure (302 nm) for 24 h. (b) PLQY (*η*) variations under UV exposure (302 nm) for 24 h. *η*_0_ refers to the original *η* before UV exposure. a.u., arbitrary units.

**
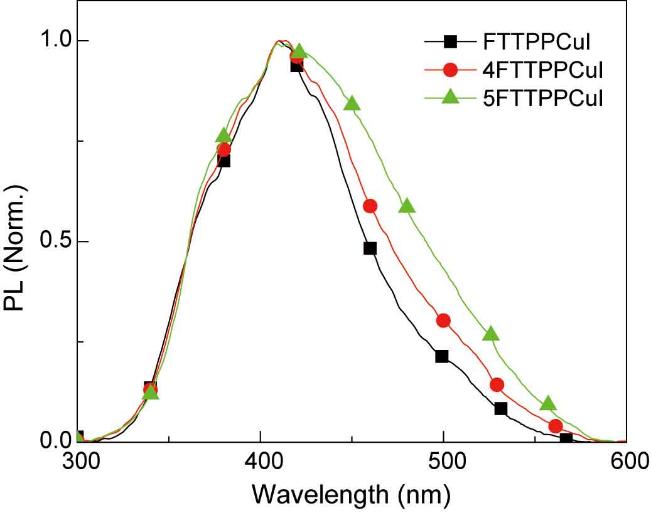
**

**Fig.** **S38.** PL spectra of **FTTPPCuI**, **4FTTPPCuI**, and **5FTTPPCuI** in dilute DCM solution (10^-6^ mol L^-1^) at room temperature. Excitation wavelength is 290 nm.

**
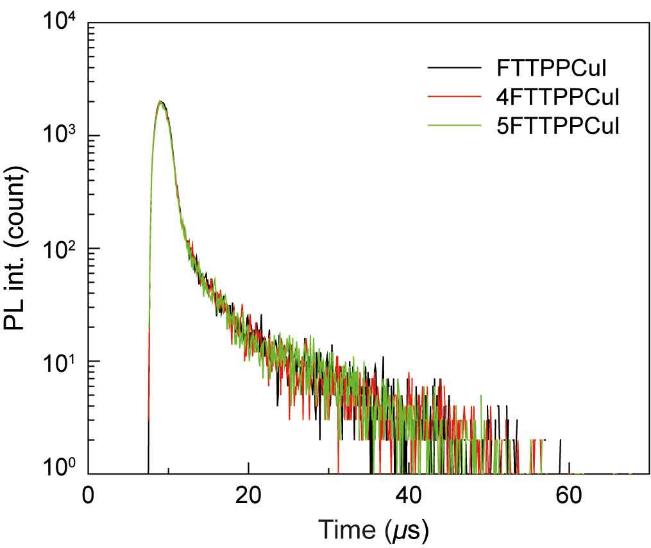
**

**Fig.** **S39.** Time decay curves of **FTTPPCuI**, **4FTTPPCuI**, and **5FTTPPCuI** in dilute DCM solution (10^-6^ mol L^-1^) at room temperature, emission peak wavelength is chosen as 410 nm, and excitation wavelength is 290 nm.


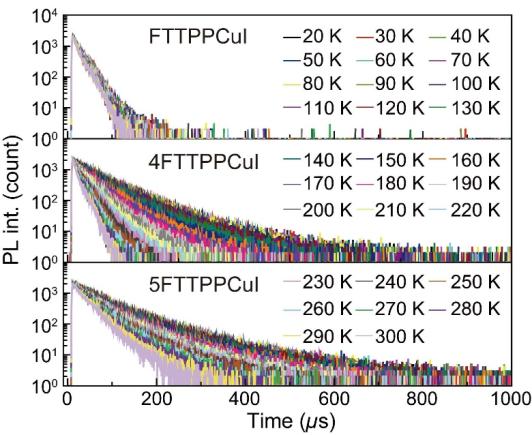


**Fig.** **S40.** Temperature-dependent time decay curves for **nFTTPPCuI** neat films in the range of 20 to 300 K with an interval of 10 K. For **FTTPPCuI**, **4FTTPPCuI**, and **5FTTPPCuI**, emission peak wavelengths are chosen as 588, 589, and 590 nm, respectively, and excitation wavelength is 350 nm.


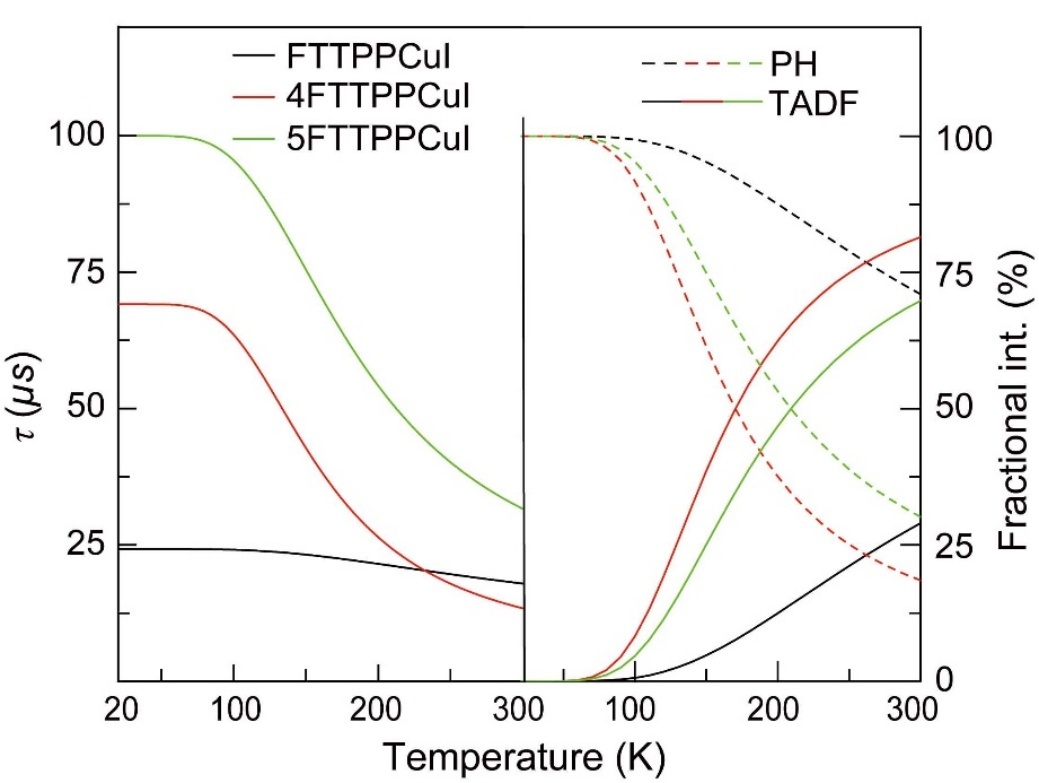


**Fig.** **S41.** Lifetime *vs*. temperature relationship (left) and temperature-dependent TADF:PH ratios (right) for **nFTTPPCuI** neat films simulated with time decay data from Fig. S22.


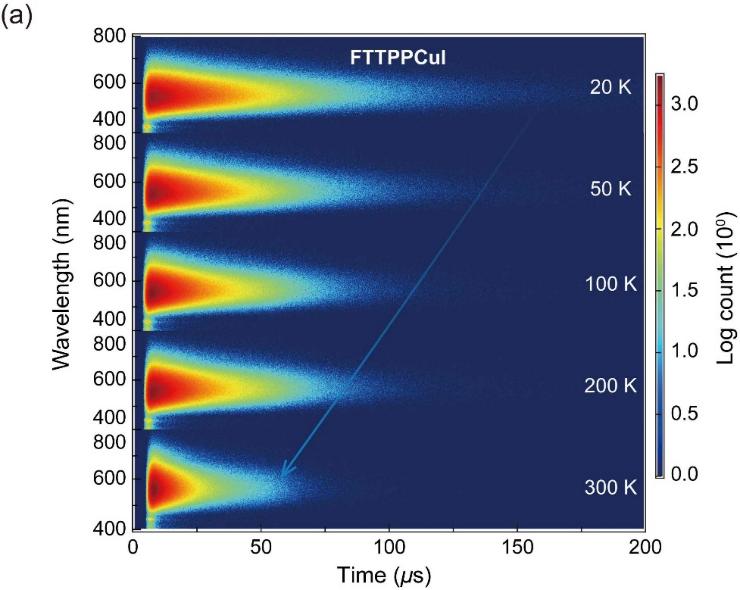

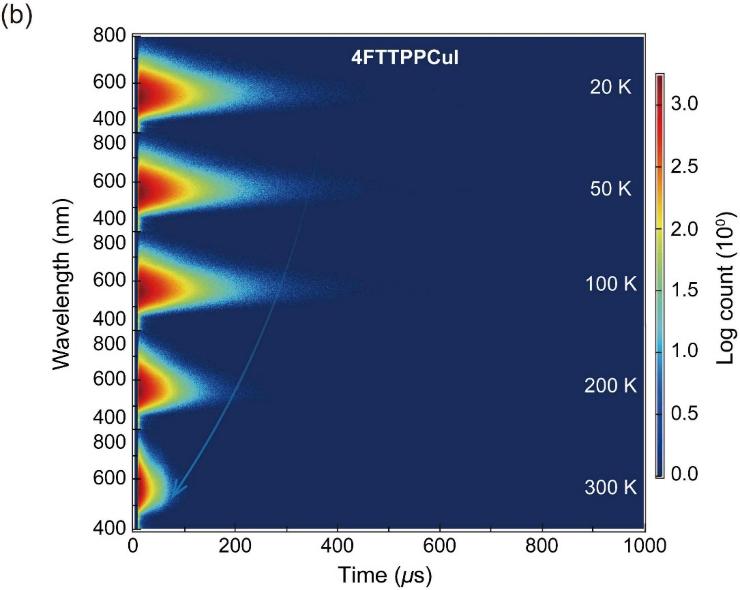


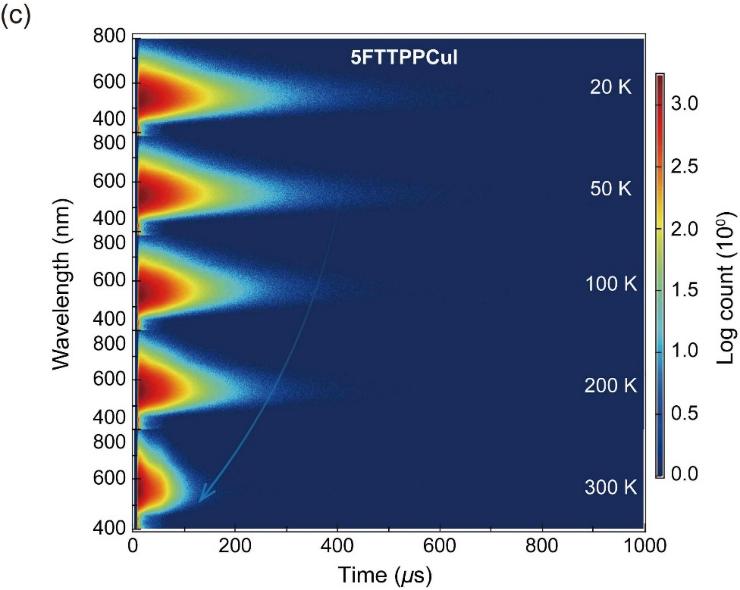


**Fig.** **S42.** TRES of neat films at 20, 50, 100, 200, and 300 K. Solid arrow indicates the variation tendency of decay duration. Excitation wavelength is 350 nm. (a) **FTTPPCuI.** (b) **4FTTPPCuI.** (c) **5FTTPPCuI.**


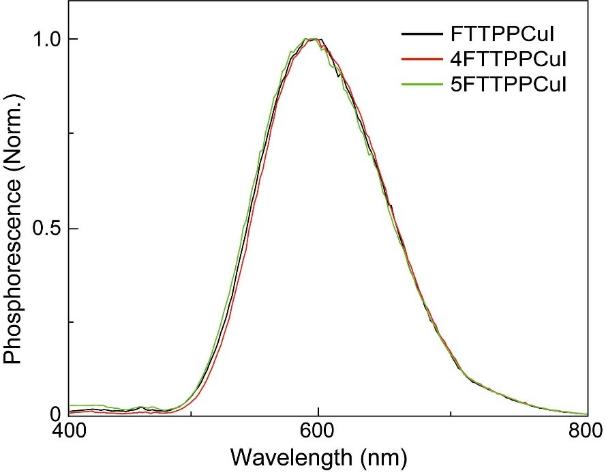


**Fig.** **S43.** Time-resolved PH spectra after 200 *µ*s of **FTTPPCuI**, **4FTTPPCuI**, and **5FTTPPCuI** neat films at 77 K. Excitation wavelength is 350 nm.

**
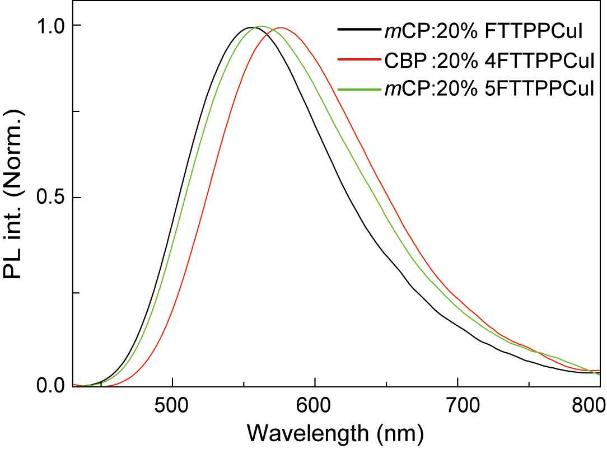
**

**Fig.** **S44.** PL spectra of **nFTTPPCuI** doped films at room temperature. Excitation wavelength is 350 nm. Emission peak wavelengths of ***m*CP:20%** **FTTPPCuI**, **CBP:20%** **4FTTPPCuI**, and ***m*CP:20%** **5FTTPPCuI** doped films are 555, 575, and 563 nm, respectively.


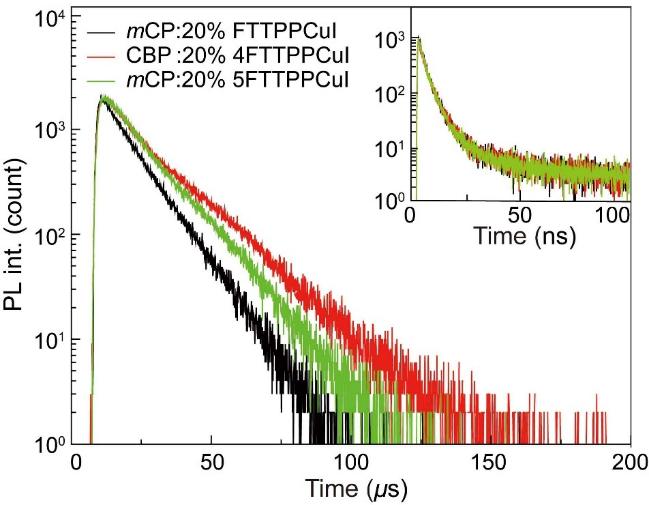


**Fig.** **S45.** Time decay curves at microsecond and nanosecond (inset) scale of **nFTTPPCuI** doped films at room temperature. For ***m*CP:20%** **FTTPPCuI**, **CBP:20%** **4FTTPPCuI**, and ***m*CP:20%** **5FTTPPCuI** doped films, emission peak wavelengths are chosen as 555, 575, and 563 nm, respectively, and excitation wavelength is 350 nm.


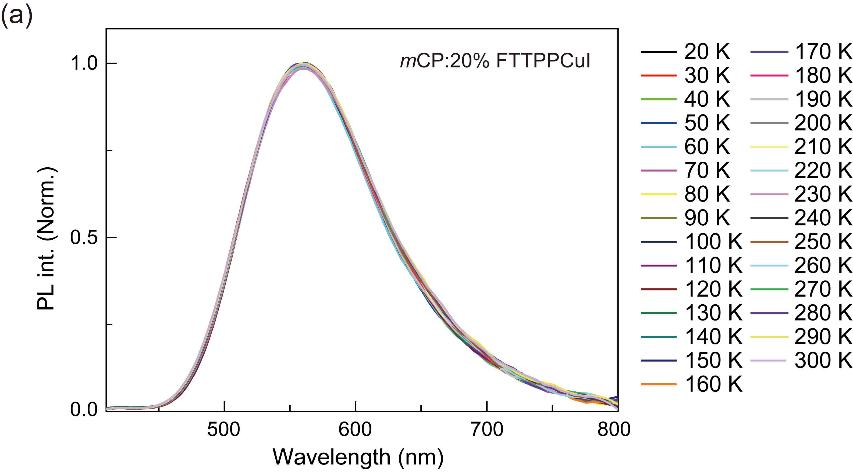


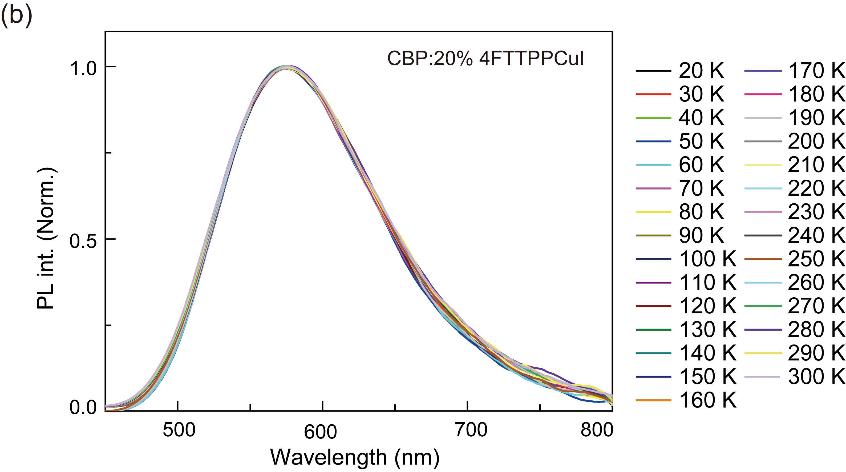


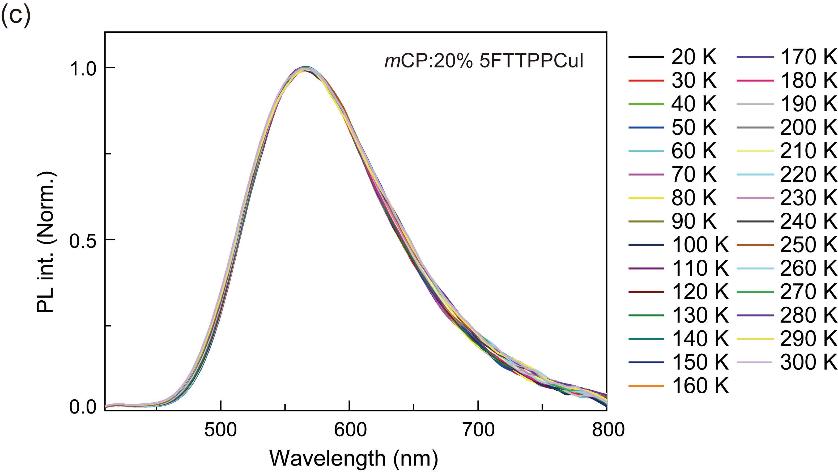


**Fig.** **S46.** Temperature-dependent PL spectra for **nFTTPPCuI** doped films during temperature increasing from 20 to 300 K with an interval of 10 K. Excitation wavelength is 350 nm. (a) ***m*CP:20%** **FTTPPCuI.** (b) **CBP:20%** **4FTTPPCuI.** (c) ***m*CP:20%** **5FTTPPCuI.**


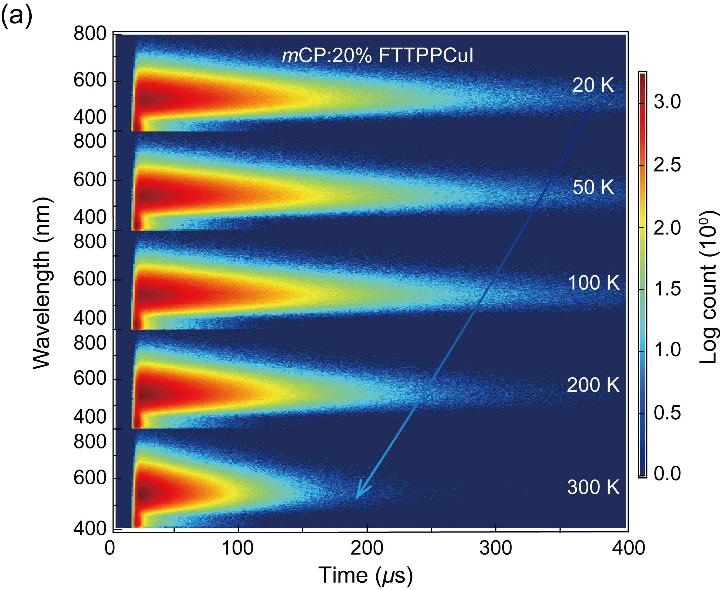

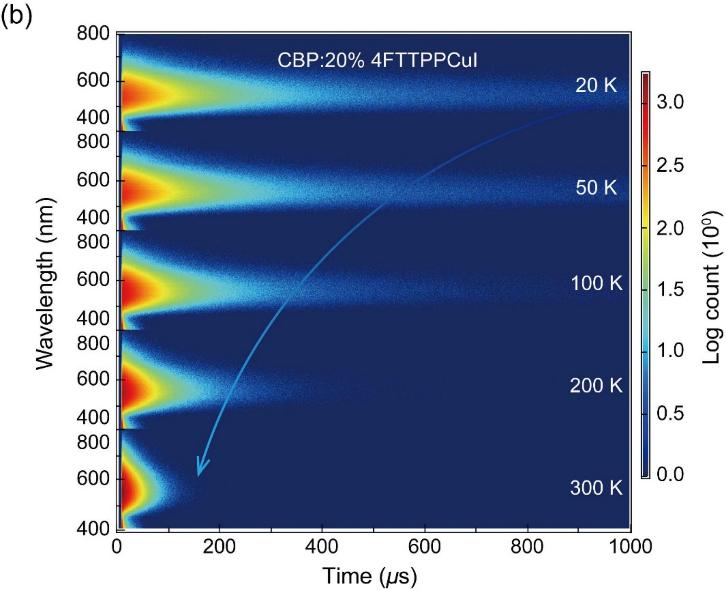

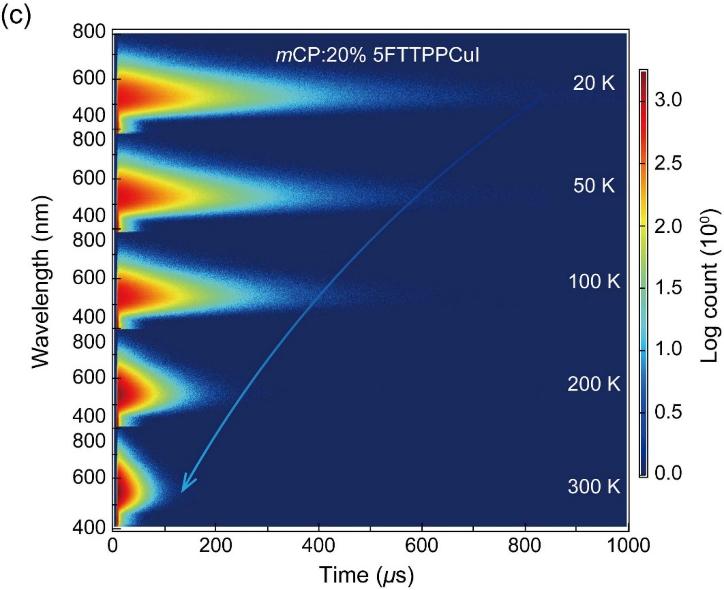


**Fig.** **S47.** TRES of **nFTTPPCuI** doped films at 20, 50, 100, 200, and 300 K. Solid arrow indicates the variation tendency of decay duration. Excitation wavelength is 350 nm. (a) ***m*CP:20%** **FTTPPCuI.** (b) **CBP:20%** **4FTTPPCuI.** (c) ***m*CP:20%** **5FTTPPCuI.**


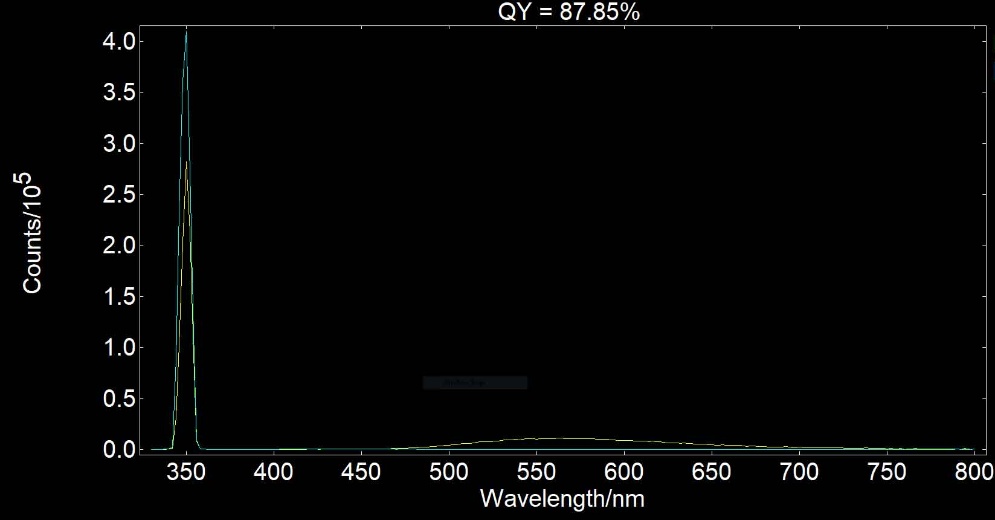


**Fig. S48**. PLQY spectra of ***m*CP:20% FTTPPCuI** film at room temperature. Excitation wavelength is 350 nm.


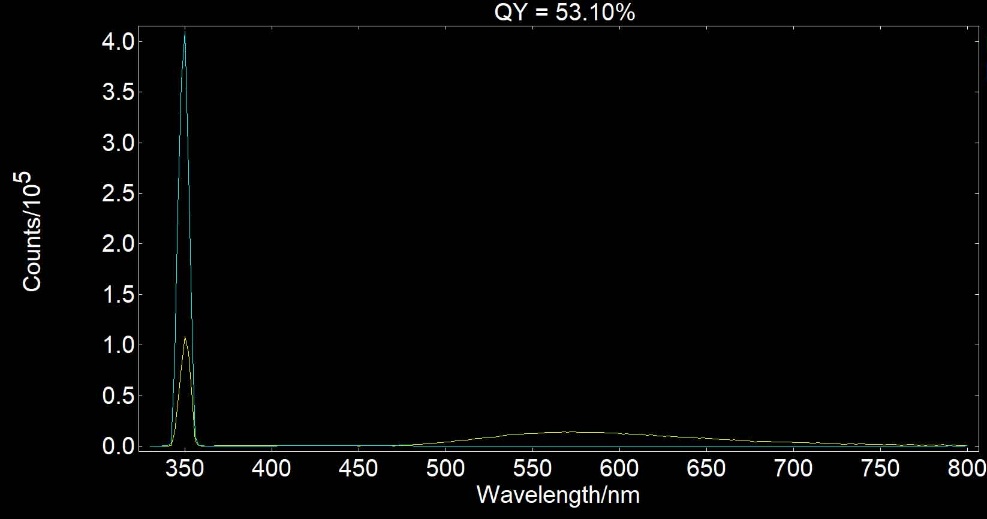


**Fig. S49.** PLQY spectra of **CBP:20% 4FTTPPCuI** film at room temperature. Excitation wavelength is 350 nm.


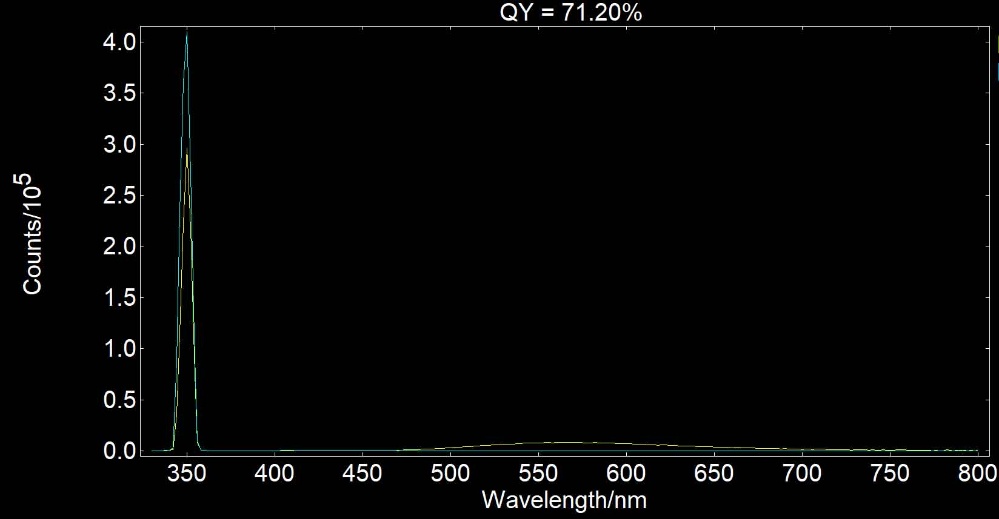


**Fig. S50.** PLQY spectra of ***m*CP:20% 5FTTPPCuI** film at room temperature. Excitation wavelength is 350 nm.

### Table S8. Physical properties of Cu(I) complexes.

| Cu(I) complexes | *λ*_Abs._ (nm) | *λ*_PL_ (nm) | S_1_ /T_1_ (eV) | Δ*E*_ST_ (eV) | *T_m_/T_d_* (^o^C) | HOMO (eV) | LUMO (eV) |
| --- | --- | --- | --- | --- | --- | --- | --- |
| **TTPPCuI** | 227, 283, 320^[a]^ | 521^[b]^ | 2.380/2.335^[c]^  2.630/2.574^[d]^ | 0.045^[c]^  0.056^[d]^ | 306/399^[e]^ | -5.31^[f]^  -4.78^[d]^ | -2.58^[f]^  -1.47^[d]^ |
| **FTTPPCuI** | 227, 280, 286^[a]^ 240, 349, 370^[b]^ | 588^[b]^ | 2.109/2.067^[c]^  2.624/2.569^[d]^ | 0.042^[c]^  0.055^[d]^ | 316/429^[e]^ | -5.26^[f]^  -4.77^[d]^ | -2.75^[f]^  -1.57^[d]^ |
| **4FTTPPCuI** | 227, 276, 284^[a]^ 240, 350, 370^[b]^ | 589^[b]^ | 2.105/2.070^[c]^  2.553/2.503^[d]^ | 0.035^[c]^  0.050^[d]^ | 317/365^[e]^ | -5.36^[f]^  -4.99^[d]^ | -2.88^[f]^  -1.88^[d]^ |
| **5FTTPPCuI** | 227,276, 285^[a]^ 240, 350, 370^[b]^ | 590^[b]^ | 2.107/2.072^[c]^  2.565/2.515^[d]^ | 0.035^[c]^  0.050^[d]^ | 322/344^[e]^ | -5.46^[f]^  -5.16^[d]^ | -2.84^[f]^  -2.07^[d]^ |

[a] In DCM solution (10^-6^ mol L^-1^); [b] in neat film; [c] estimated according the fluorescence and PH peak wavelengths; (Figs S18 and S25) [d] Gaussian simulation results of single molecules; [e] temperature at weight loss of 5%; [f] calculated according to CV results with Lippert-Mataga relationship.

### Table S9. Key transition parameters of Cu(I) complexes.

| Cu(I) complexes | *ϕ*_PL_^[a]^ (%) | *τ*_PF_^[b]^ (ns) | *τ*_DF_^[c]^ (*µ*s) | *ϕ*_PF_^[d]^ (%) | *ϕ*_DF_^[e]^ (%) | *k*_PF_^[f]^ (10^7^) | *k*_DF_^[g]^ (10^4^) | *k*_ISC_^[h]^ (10^7^) | *k*_RISC_^[i]^ (10^4^) | $k_{r}^{S}$^[j]^ (10^7^) | $k_{nr}^{S}$^[k]^ (10^7^) | $k_{r}^{S}{/k}_{nr}^{S}$^[l]^ | *ϕ*_ISC_^[m]^ (%) | *ϕ*_RISC_^[n]^ (%) |
| --- | --- | --- | --- | --- | --- | --- | --- | --- | --- | --- | --- | --- | --- | --- |
| ***m*CP:20% FTTPPCuI** | 87.85 | 30.52 | 10.30 | 32.45 | 55.40 | 1.06 | 5.38 | 0.67 | 14.56 | 0.34 | 0.048 | 7.08 | 63.07 | 95.71 |
| **CBP:20% 4FTTPPCuI** | 53.10 | 18.84 | 17.39 | 12.20 | 40.89 | 0.64 | 2.35 | 0.50 | 10.23 | 0.079 | 0.070 | 1.13 | 77.02 | 90.27 |
| ***m*CP:20% 5FTTPPCuI** | 71.20 | 26.23 | 13.20 | 20.69 | 50.51 | 0.78 | 3.82 | 0.56 | 13.17 | 0.16 | 0.066 | 2.42 | 70.95 | 92.28 |

[a]Absolute PLQY evaluated using an integrating sphere; [b]PF and [c]DF lifetime; Quantum efficiencies of [d]PF and [e]DF, estimated according to the corresponding proportions in transient decay curves; Rate constants of [f]PF, [g]DF, [h]intersystem crossing (ISC), [i]reverse intersystem crossing (RISC), [j]singlet radiation and [k]singlet non-radiation; [l]Ratio of the singlet radiative rate to non-radiative rate constant; Quantum efficiencies of [m]ISC and [n]RISC.

### Electroluminescent Performance


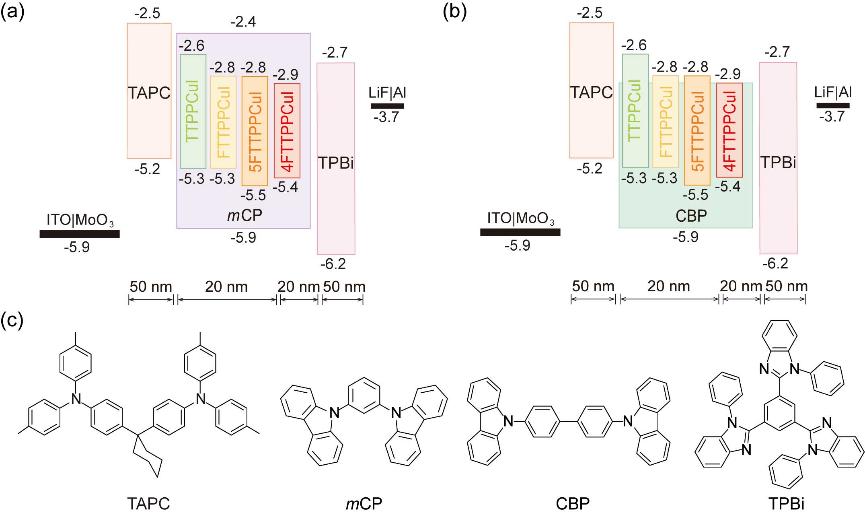


**Fig.** **S51.** Device structures and energy level diagram of the devices. (a) Device structures of ITO|MoO_3_ (6 nm)|TAPC (50 nm)|Host: *x*% nFTTPPCuI (20 nm)|TPBi (50 nm)|LiF (1 nm)|Al (100 nm), in which *m*CP was used as host. (b) Device structures of ITO|MoO_3_ (6 nm)|TAPC (50 nm)|Host: *x*% nFTTPPCuI (20 nm)|TPBi (50 nm)|LiF (1 nm)|Al (100 nm), in which CBP was used as host; (c) Chemical structures of TAPC, *m*CP, CBP, and TPBi.


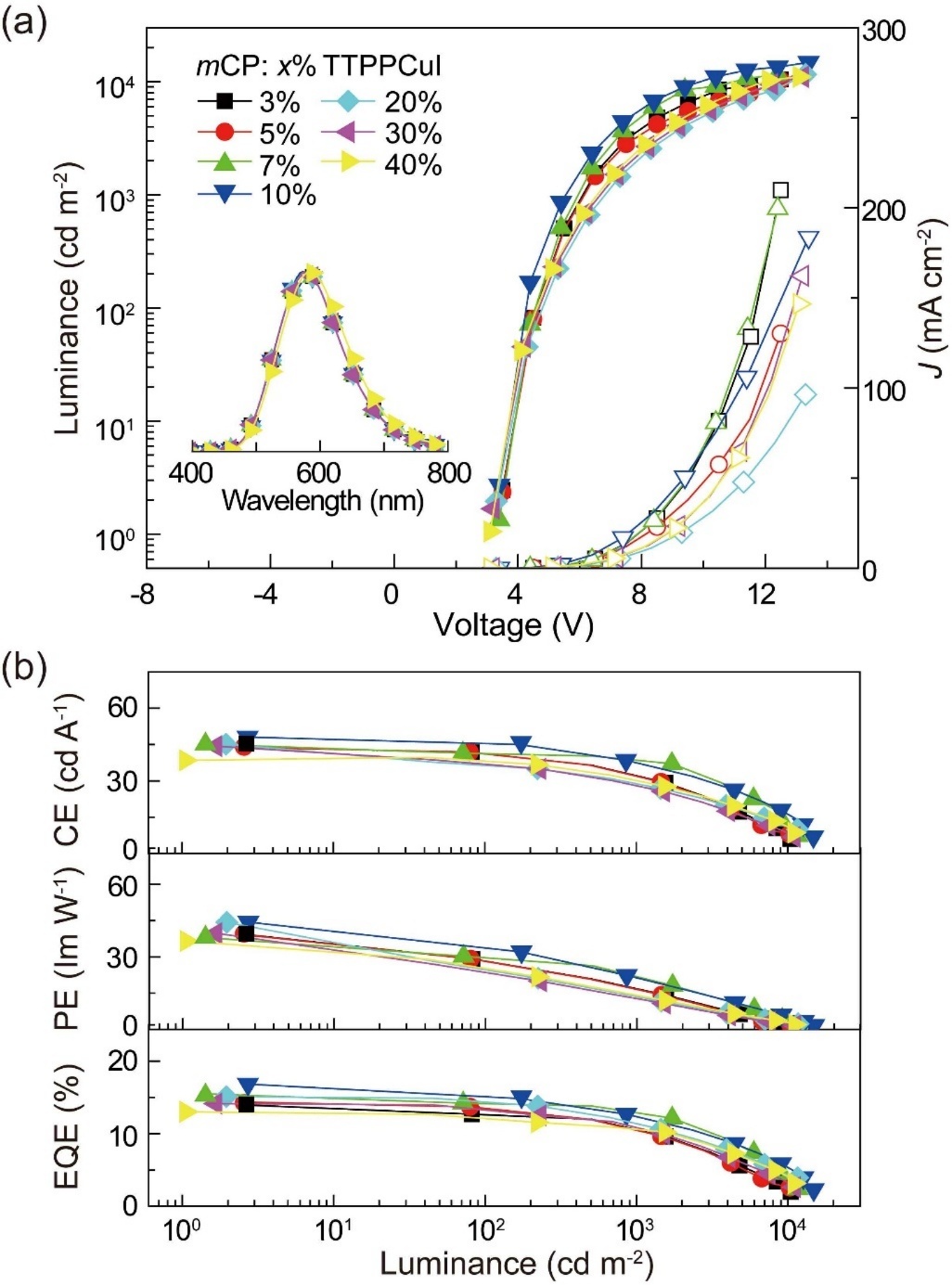


**Fig. S52.** Doping concentration dependence of EL performance for ***m*CP:*x*%** **TTPPCuI** based OLEDs (*x* = 3, 5, 7, 10, 20, 30, and 40). (a) EL spectra (inset) and Current density (*J*)-Voltage-Luminance characteristics. (b) Efficiencies *vs*. Luminance relationships of the devices.


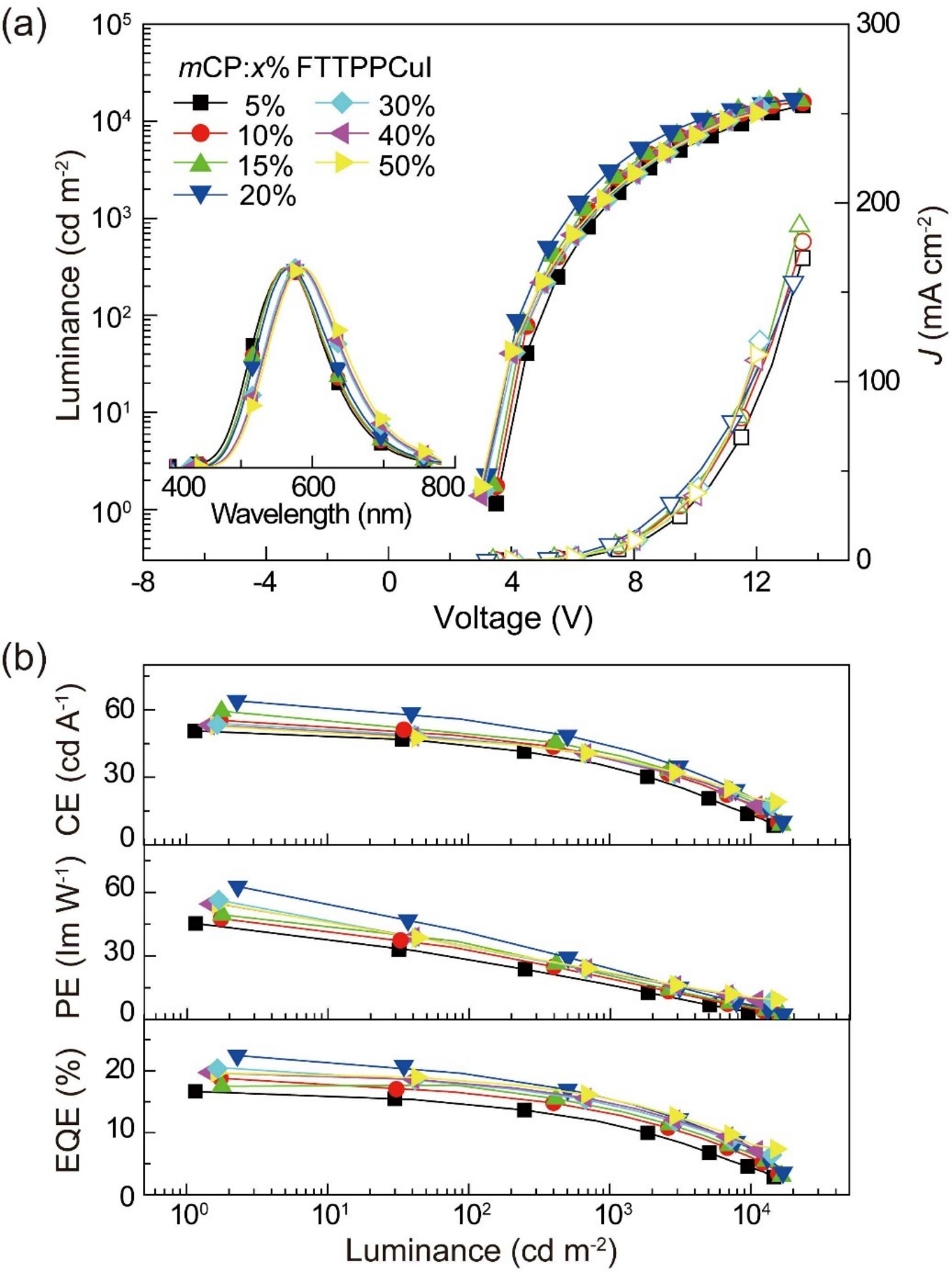


**Fig.** **S53.** Doping concentration dependence of EL performance of ***m*CP:*x*%** **FTTPPCuI** based OLEDs (*x* = 5, 10, 15, 20, 30, 40, and 50). (a) EL spectra (inset) and *J*-Voltage-Luminance characteristics. (b) Efficiencies *vs*. Luminance relationships of the devices.


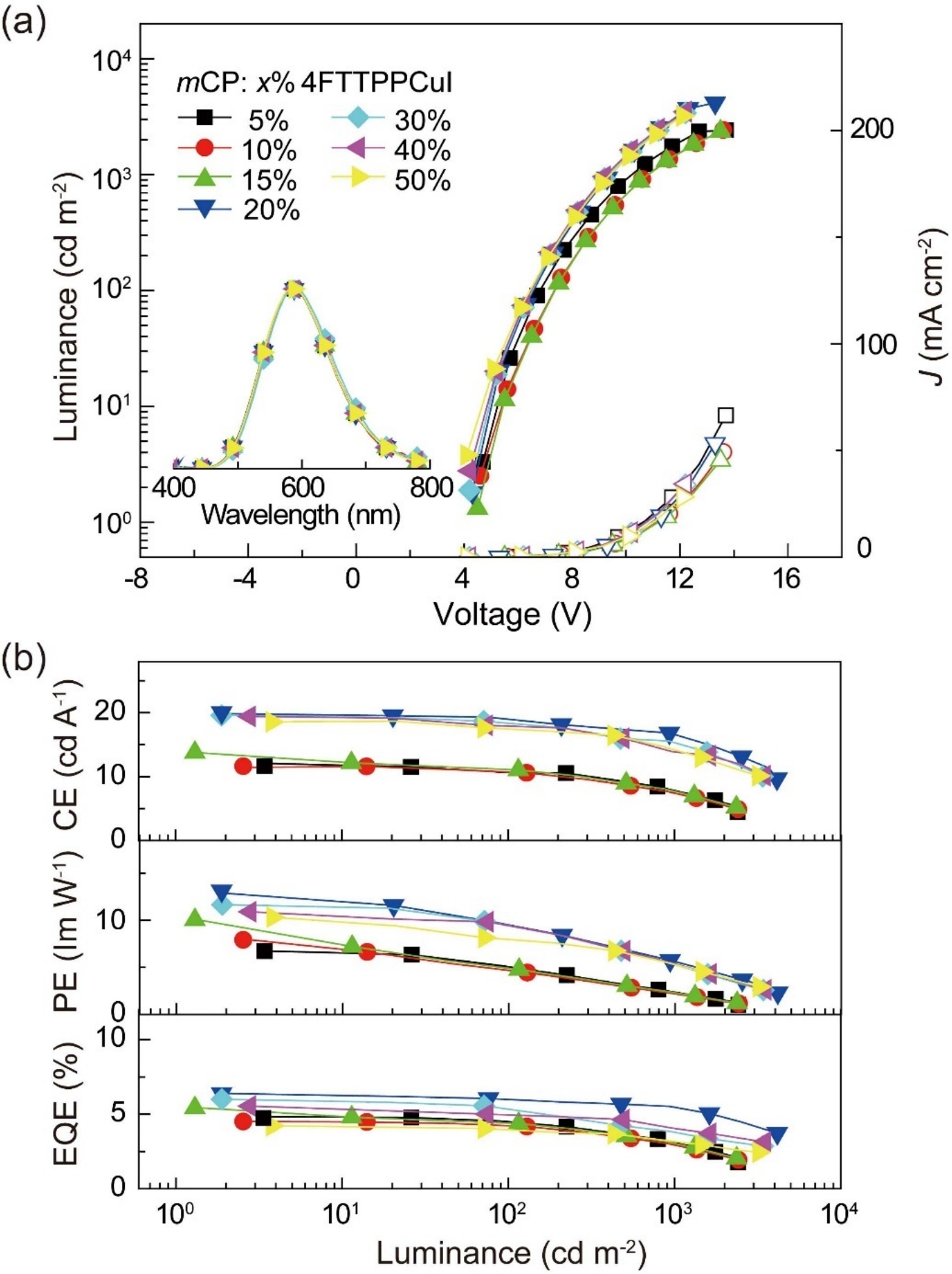


**Fig.** **S54.** Doping concentration dependence of EL performance of ***m*CP:*x*%** **4FTTPPCuI** based OLEDs (*x* = 5, 10, 15, 20, 30, 40, and 50). (a) EL spectra (inset) and *J*-Voltage-Luminance characteristics. (b) Efficiencies *vs*. Luminance relationships of the devices.


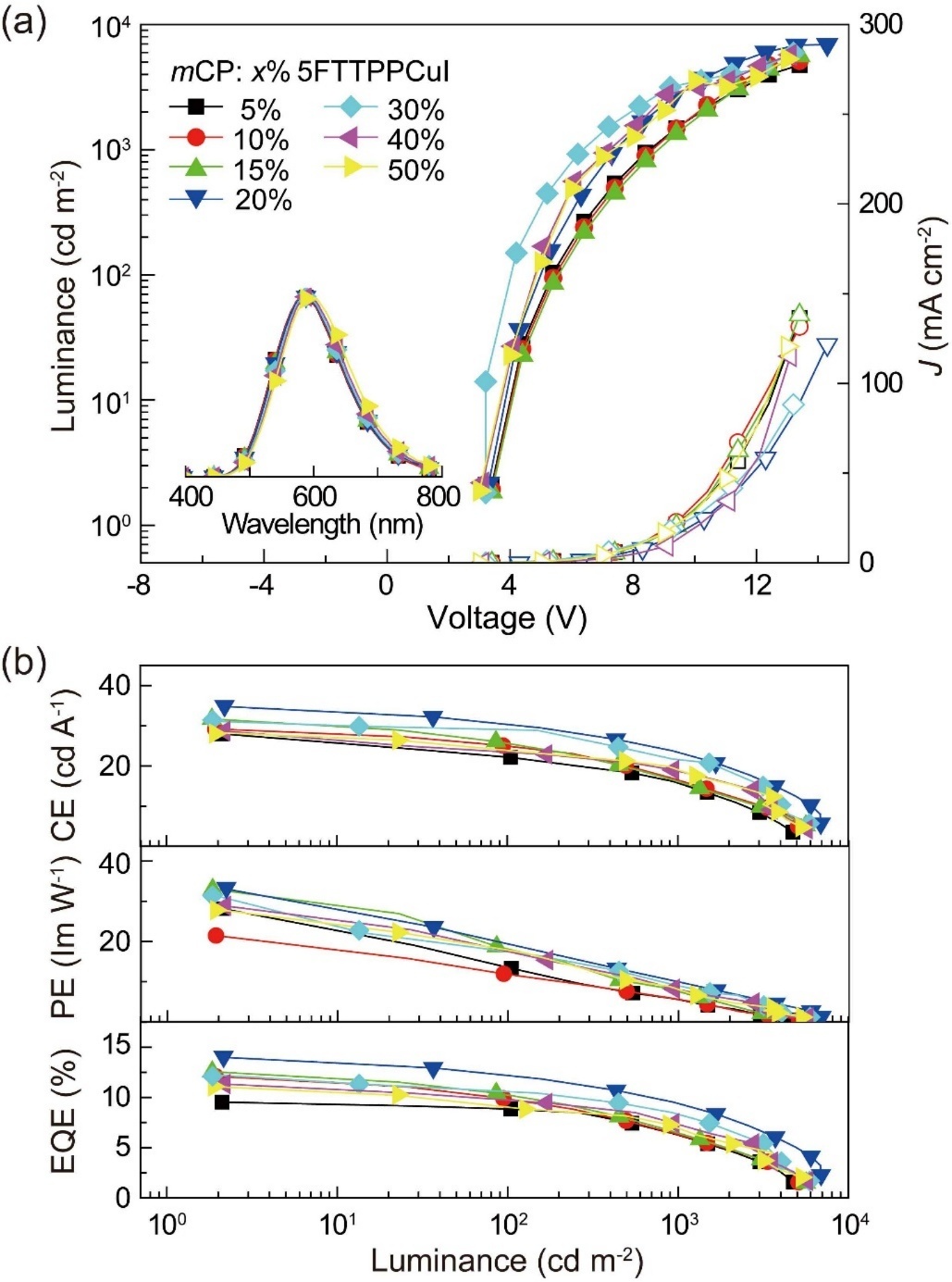


**Fig.** **S55.** Doping concentration dependence of EL performance for ***m*CP:*x*%** **5FTTPPCuI** based OLEDs (*x* = 5, 10, 15, 20, 30, 40, and 50). (a) EL spectra (inset) and -*J*-Voltage-Luminance characteristics. (b) Efficiencies *vs*. Luminance relationships of the devices.


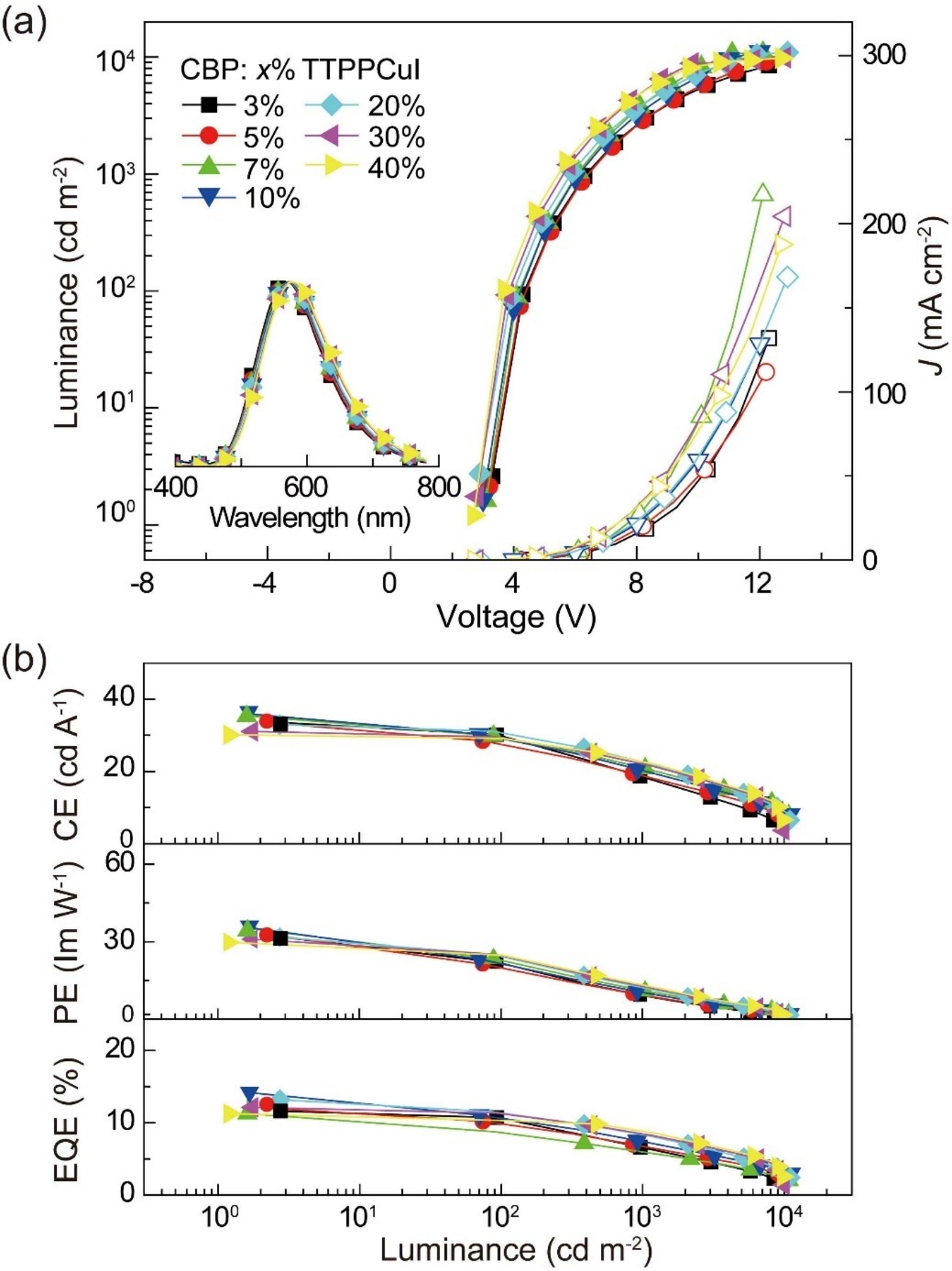


**Fig. S56.** Doping concentration dependence of EL performance for **CBP:*x*%** **TTPPCuI** based OLEDs (*x* = 3, 5, 7, 10, 20, 30, and 40). (a) EL spectra (inset) and *J*-Voltage-Luminance characteristics. (b) Efficiencies *vs*. Luminance relationships of the devices.


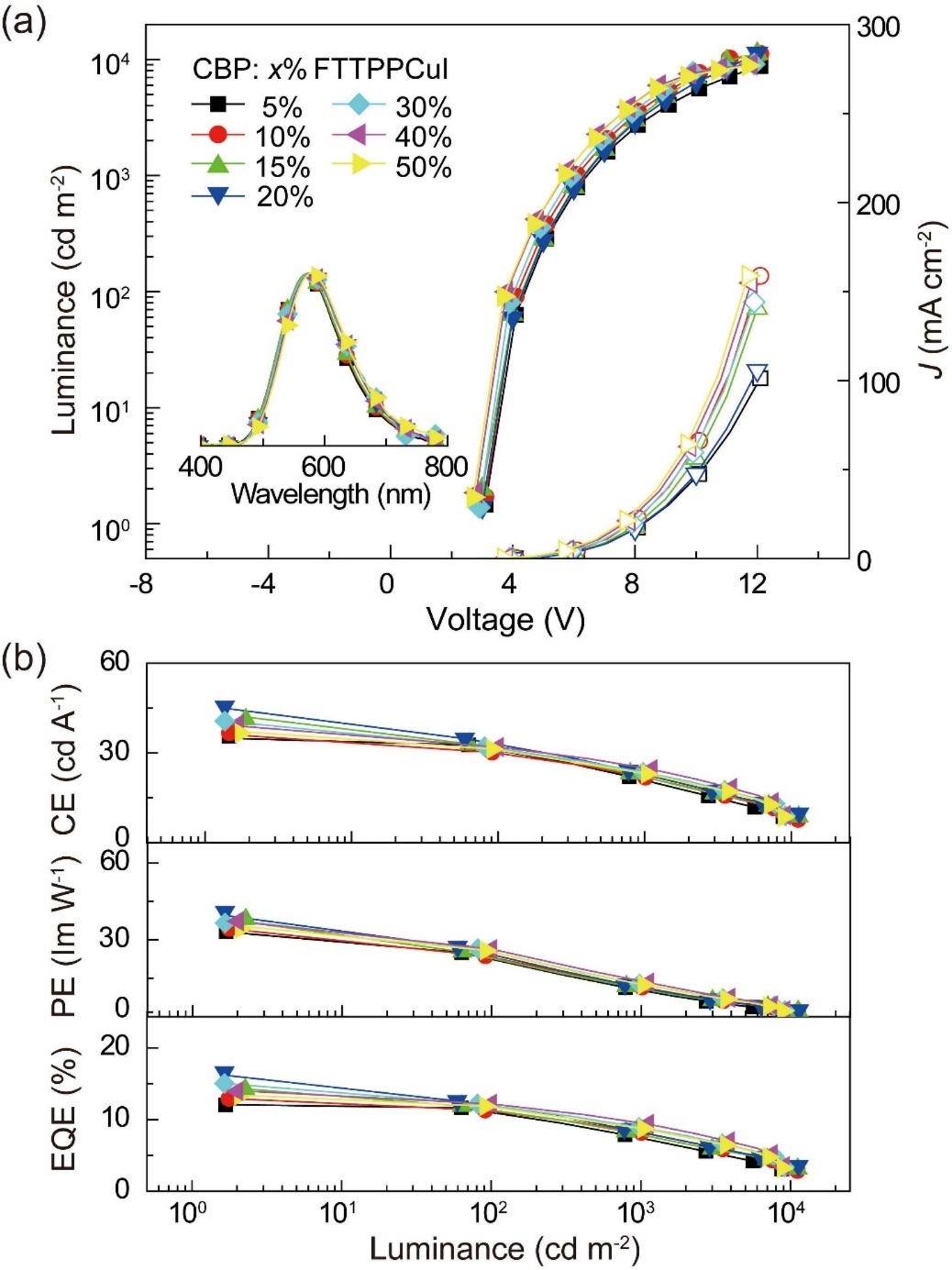


**Fig. S57.** Doping concentration dependence of EL performance for **CBP:*x*%** **FTTPPCuI** based OLEDs (*x* = 5, 10, 15, 20, 30, 40, and 50). (a) EL spectra (inset) and *J*-Voltage-Luminance characteristics. (b) Efficiencies *vs*. Luminance relationships of the devices.


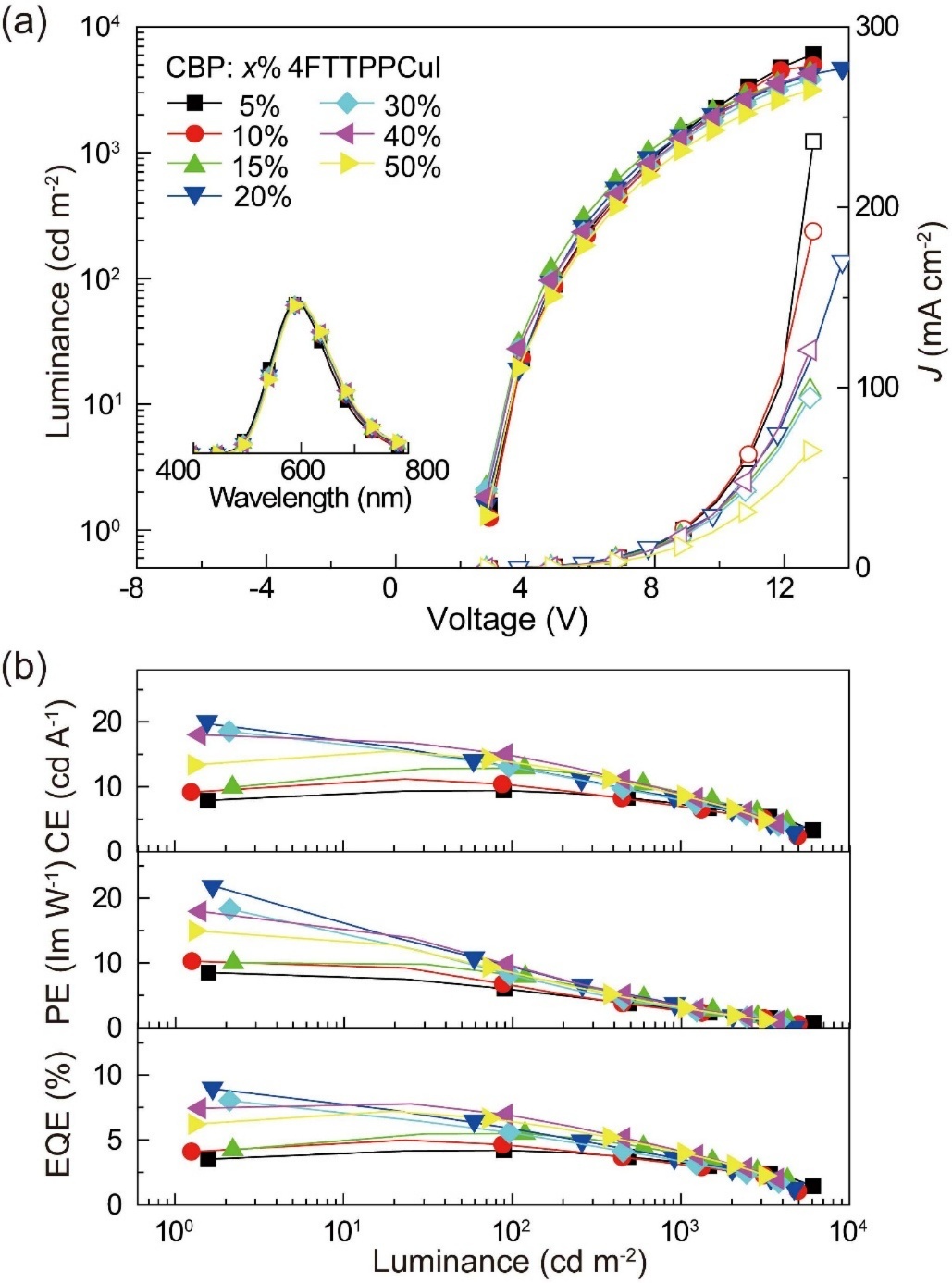


**Fig. S58.** Doping concentration dependence of EL performance for **CBP:*x*%** **4FTTPPCuI** based OLEDs (*x* = 5, 10, 15, 20, 30, 40, and 50). (a) EL spectra (inset) and *J*-Voltage-Luminance characteristics. (b) Efficiencies *vs*. Luminance relationships of the devices.


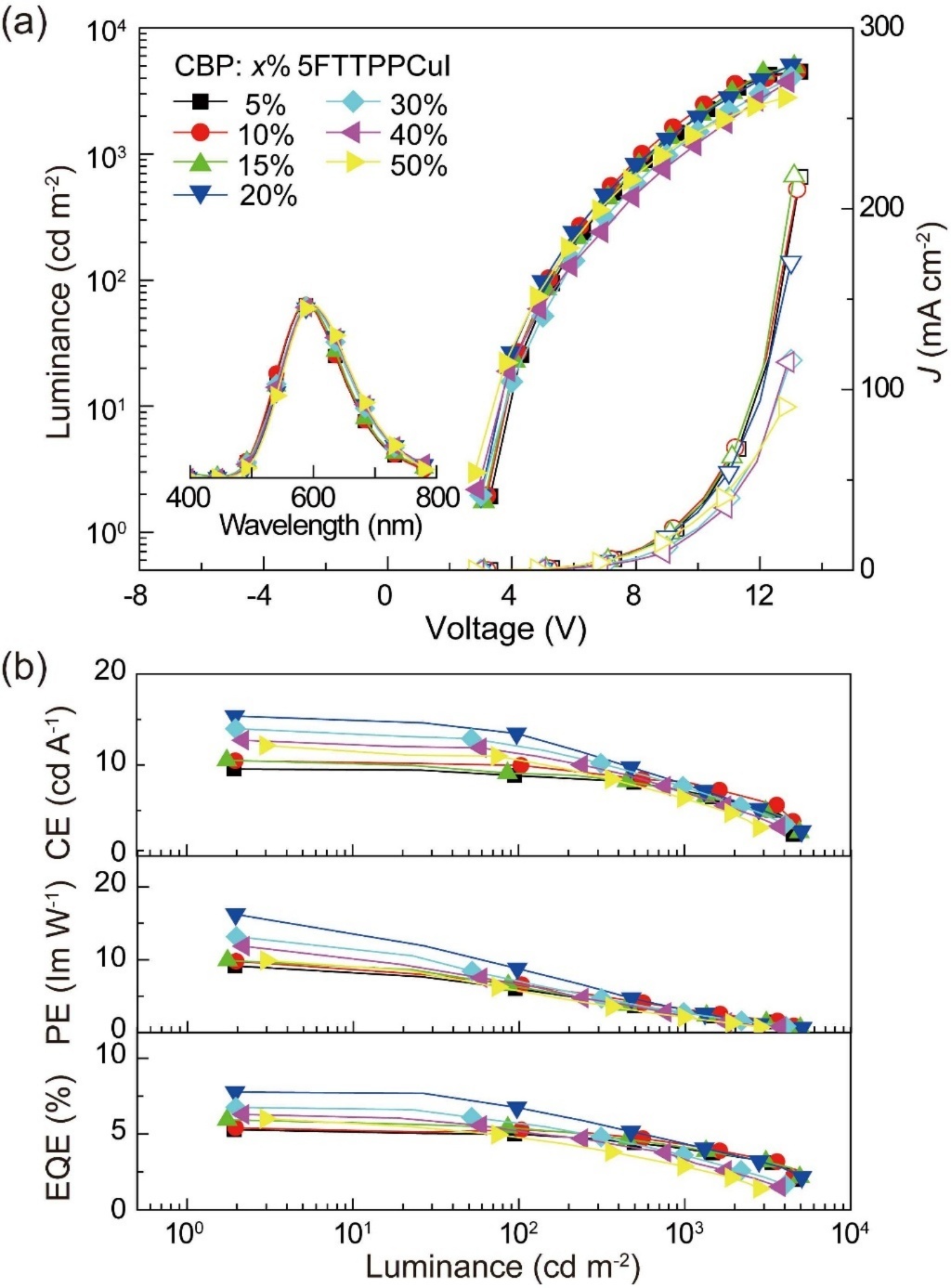


**Fig. S59.** Doping concentration dependence of EL performance for **CBP:*x*%** **5FTTPPCuI** based OLEDs (*x* = 5, 10, 15, 20, 30, 40, and 50). (a) EL spectra (inset) and *J*-Voltage-Luminance characteristics. (b) Efficiencies *vs*. Luminance relationships of the devices.


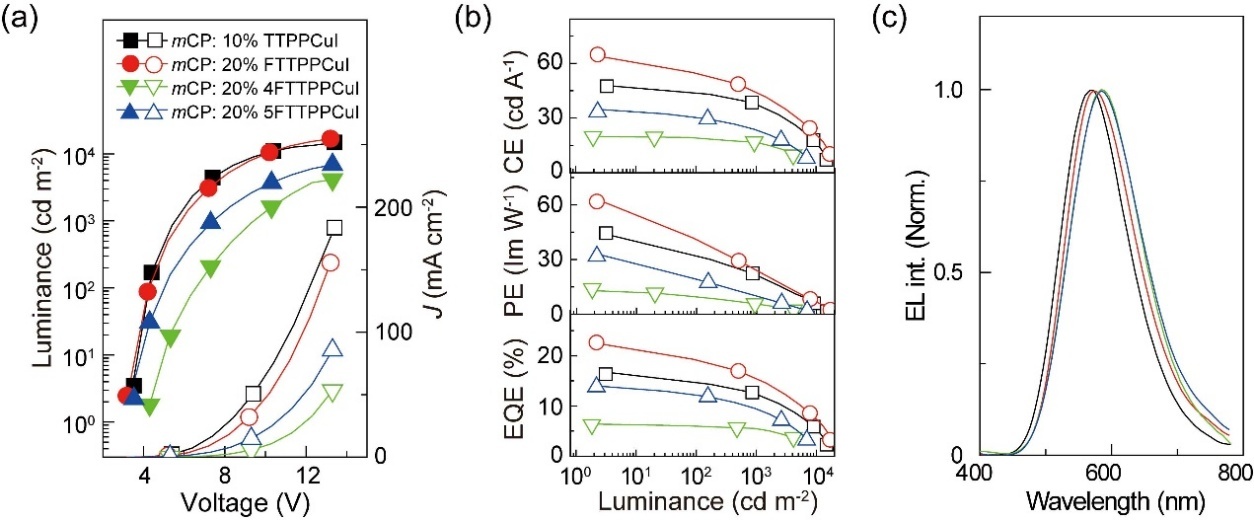


**Fig. S60.** EL optimal performance of **nFTTPPCuI** with *m*CP as host material. (a) *J* (open symbols)-Luminance (solid symbols)-Voltage characteristics of the devices at the optimal doping concentrations (*x*%); (b) Efficiencies *vs.* luminance relationships of the devices. CE and PE refer to current and power efficiencies, respectively; (c) EL spectra at the respective optimal doping concentrations.


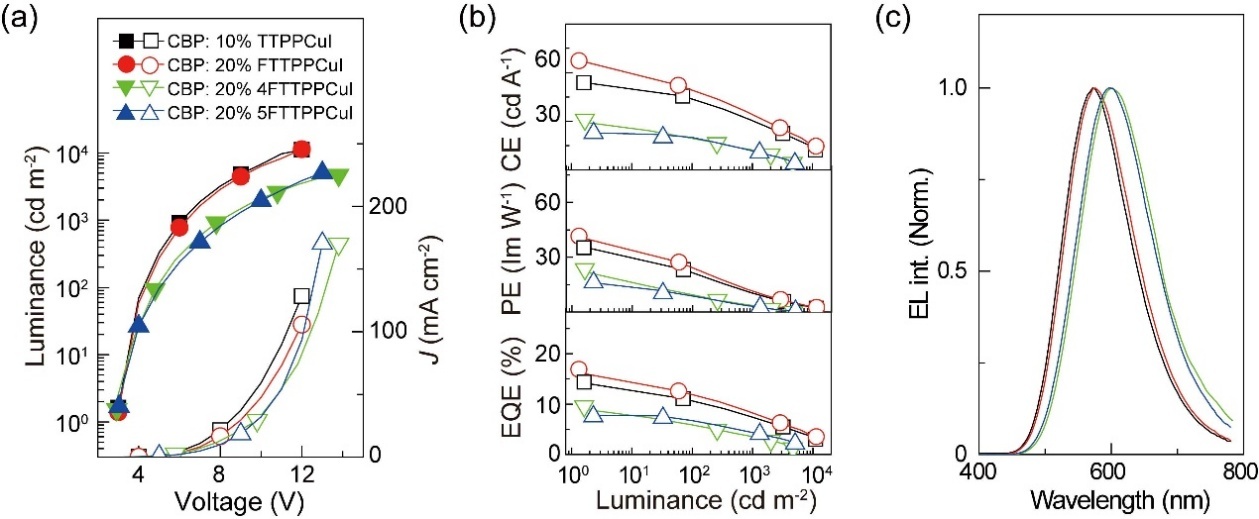


**Fig. S61.** EL optimal performance of **nFTTPPCuI** with CBP as host material. (a) *J* (open symbols)-Luminance (solid symbols)-Voltage characteristics of the devices at the optimal doping concentrations (*x*%); (b) Efficiencies *vs.* luminance relationships of the devices; (c) EL spectra at the respective optimal doping concentrations.

**
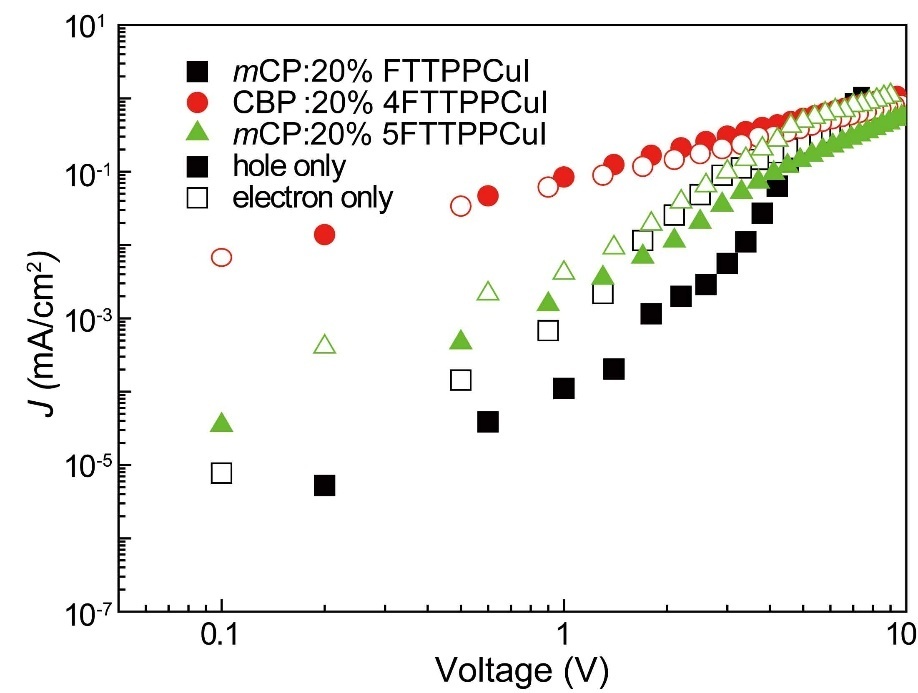
**

**Fig. S62.** Voltage-*J* curves characteristics of single-carrier-transporting devices based on nFTTPPCuI with configurations of ITO|MoO_3_ (6 nm)|Host:20% **nFTTPPCuI** (n=1, 4, and 5) (100 nm)|MoO_3_ (6 nm)|Al for hole only and ITO|LiF (1 nm)|Host:20% **nFTTPPCuI** (n=1, 4, and 5) (100 nm)|LiF (1 nm)|Al for electron only, in which MoO_3_ and LiF served as hole- and electron-injecting layers, respectively.


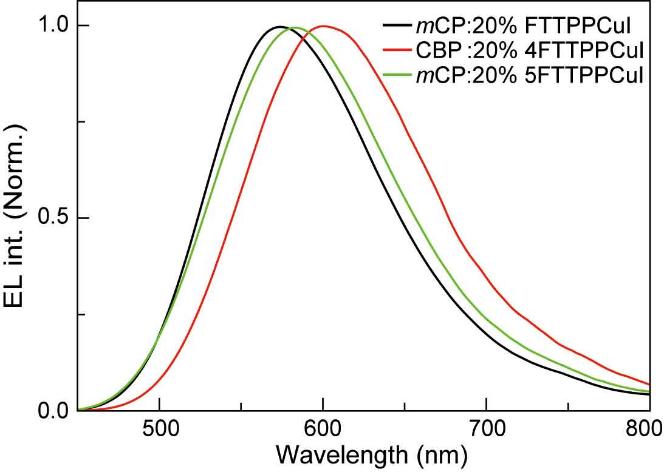


**Fig. S63.** EL spectra of **nFTTPPCuI** doped films at room temperature. Emission peak wavelengths of ***m*CP:20%** **FTTPPCuI**, **CBP:20%** **4FTTPPCuI**, and ***m*CP:20%** **5FTTPPCuI** doped films are 574, 603, and 584 nm, respectively.


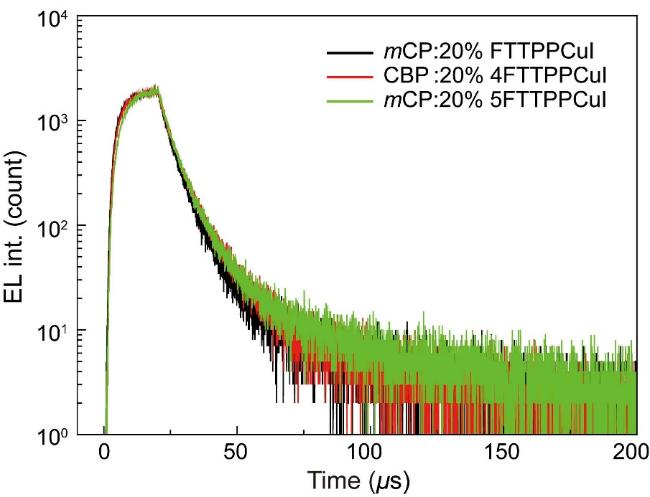


**Fig. S64.** EL time decay curves of **nFTTPPCuI** doped films at room temperature. For ***m*CP:20%** **FTTPPCuI**, **CBP:20%** **4FTTPPCuI**, and ***m*CP:20%** **5FTTPPCuI** doped films, emission peak wavelengths are chosen as 574, 603, and 584 nm, respectively.


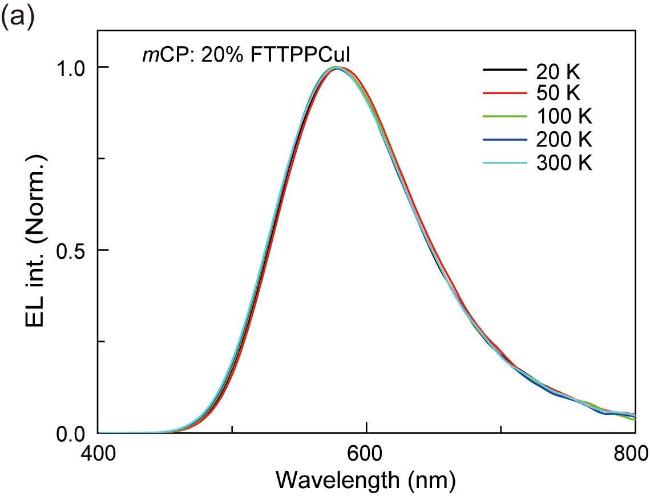


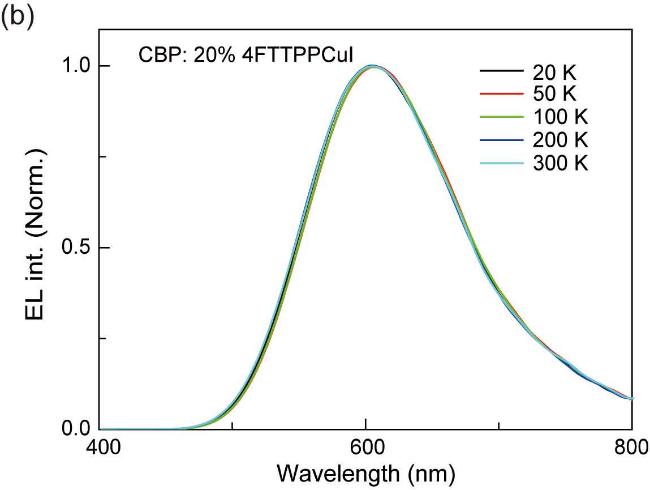


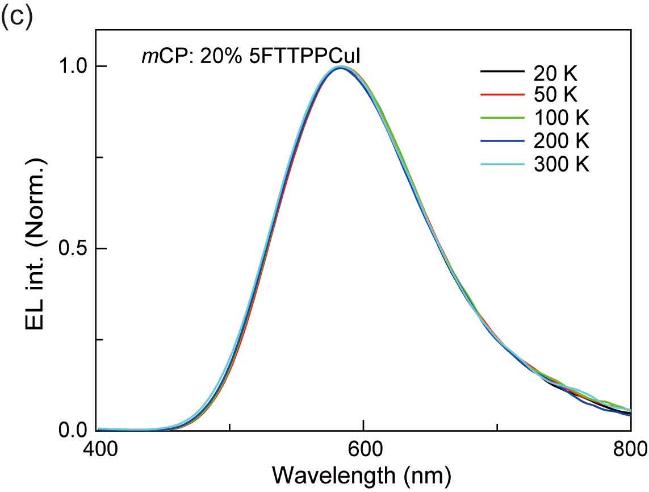


**Fig. S65.** Temperature-dependent EL spectra for **nFTTPPCuI** doped films at 20, 50, 100, 200, and 300 K. (a) ***m*CP:20%** **FTTPPCuI.** (b) **CBP:20% 4FTTPPCuI.** (c) ***m*CP:20%** **5FTTPPCuI.**

### Table S10. Electroluminescent performance of OLEDs with *m*CP as host material.

| EML | *x* (wt%) | *V*^[a]^ (V) | *L*_max_^[b]^  (cd m^-2^) | *η*^[c]^ | | | *λ*_EL_ (nm)/  CIE (x, y)^[d]^ |
| --- | --- | --- | --- | --- | --- | --- | --- |
|  |  |  |  | *η*_CE_ (cd A^-1^) | *η*_PE_ (lm W^-1^) | *η*_EQE_ (%) |  |
| ***m*CP:*x*% TTPPCuI** | 3 | 3.5, 4.6, 6.1 | 10460 | 43.9, 41.2, 32.4 | 39.4, 28.5, 17.1 | 14.0, 12.5, 10.5 | 571/(0.45, 0.51) |
|  | 5 | 3.5, 4.6, 6.2 | 10300 | 43.9, 41.0, 31.6 | 39.4, 28.5, 16.8 | 14.4, 13.5, 10.5 | 571/(0.45, 0.51) |
|  | 7 | 3.4, 4.6, 6.0 | 11620 | 43.2, 41.3, 35.6 | 38.1, 33.6, 22.5 | 15.5, 14.8, 11.5 | 572/(0.45, 0.51) |
|  | 10 | 3.4, 4.3, 5.6 | 14800 | 48.1, 45.2, 37.2 | 44.4, 33.3, 21.1 | 16.8, 15.1, 12.4 | 572/(0.45, 0.51) |
|  | 20 | 3.3, 4.8, 6.9 | 11597 | 45.1, 36.8, 29.1 | 44.3, 24.8, 14.2 | 15.1, 14.4, 11.5 | 575/(0.47, 0.51) |
|  | 30 | 3.2, 4.7, 6.7 | 11050 | 44.3, 36.8, 27.6 | 39.7, 23.6, 12.4 | 14.1, 13.3, 10.9 | 578/(0.47, 0.51) |
|  | 40 | 3.1, 4.6, 6.6 | 11020 | 38.5, 27.8, 29.4 | 36.6, 25.1, 13.5 | 13.0, 12.0, 10.5 | 580/(0.47, 0.51) |
| ***m*CP:*x*% FTTPPCuI** | 5 | 3.5, 5.0, 6.7 | 14600 | 50.6, 43.7, 34.3 | 45.4, 28.5, 16.6 | 16.7, 14.6, 11.4 | 564/(0.43, 0.52) |
|  | 10 | 3.5, 4.7, 6.4 | 15680 | 55.3, 48.4, 38.6 | 47.6, 33.2, 19.1 | 18.8, 16.3, 13.1 | 566/(0.44, 0.52) |
|  | 15 | 3.4, 4.5, 6.2 | 16520 | 59.4, 49.3, 40.5 | 49.5, 36.0, 20.9 | 17.7, 17.6, 14.0 | 570/(0.45, 0.51) |
|  | 20 | 3.2, 4.2, 5.8 | 16870 | 64.0, 55.5, 44.2 | 62.8, 42.0, 24.2 | 22.4, 19.7, 15.4 | 574/(0.47, 0.51) |
|  | 30 | 3.1, 4.6, 6.6 | 14050 | 54.1, 46.8, 38.5 | 56.8, 34.4, 21.6 | 20.6, 18.0, 14.6 | 579/(0.48, 0.48) |
|  | 40 | 3.0, 4.5, 6.5 | 13010 | 53.3, 46.1, 37.9 | 55.5, 34.7, 22.2 | 19.7, 18.0, 14.9 | 582/(0.49, 0.47) |
|  | 50 | 3.0, 4.5, 6.5 | 12110 | 52.5, 45.8, 39.0 | 54.7, 33.8, 21.9 | 19.0, 18.2, 15.5 | 585/(0.50, 0.45) |

| EML | *x* (wt%) | *V*^[a]^ (V) | *L*_max_^[b]^  (cd m^-2^) | *η*^[c]^ | | | *λ*_EL_ (nm)/  CIE (x, y)^[d]^ |
| --- | --- | --- | --- | --- | --- | --- | --- |
|  |  |  |  | *η*_CE_ (cd A^-1^) | *η*_PE_ (lm W^-1^) | *η*_EQE_ (%) |  |
| ***m*CP:*x*% 4FTTPPCuI** | 5 | 4.7, 6.8, 10.2 | 2412 | 12.1, 10.6, 7.7 | 6.7, 5.1, 2.5 | 4.8, 4.5, 3.2 | 582/(0.49, 0.47) |
|  | 10 | 4.6, 7.3, 10.9 | 2426 | 11.4, 10.6, 7.4 | 8.0, 4.7, 2.1 | 4.5, 4.3, 2.9 | 582/(0.49, 0.47) |
|  | 15 | 4.5, 7.4, 10.9 | 2366 | 13.8, 11.2, 7.6 | 10.1, 5.0, 2.3 | 5.4, 4.4, 3.0 | 584/(0.50, 0.45) |
|  | 20 | 4.3, 6.6, 9.4 | 4155 | 19.9, 10.0, 6.9 | 12.9, 9.6, 5.6 | 6.4, 6.0, 5.5 | 584/(0.50, 0.45) |
|  | 30 | 4.2, 6.5, 9.3 | 3403 | 19.6, 18.6, 15.3 | 11.6, 9.4, 5.4 | 6.0, 5.4, 3.9 | 586/(0.52, 0.44) |
|  | 40 | 4.2, 6.4, 9.3 | 3493 | 19.4, 17.9, 13.8 | 10.9, 9.4, 5.3 | 5.5, 5.0, 4.0 | 588/(0.54, 0.40) |
|  | 50 | 4.1, 6.4, 9.5 | 3204 | 18.6, 17.2, 13.8 | 10.3, 7.9, 5.3 | 4.2, 4.0, 3.1 | 588/(0.54, 0.40) |
| ***m*CP:*x*% 5FTTPPCuI** | 5 | 3.3, 5.3, 8.4 | 4747 | 28.0, 22.1, 15.9 | 28.1, 13.5, 5.6 | 9.5, 8.8, 6.3 | 582/(0.49, 0.47) |
|  | 10 | 3.3, 5.4, 8.6 | 5098 | 29.2, 25.0, 16.4 | 21.4, 12.0, 5.8 | 12.1, 9.8, 6.3 | 582/(0.49, 0.47) |
|  | 15 | 3.3, 5.4, 8.7 | 5639 | 31.7, 25.8, 16.4 | 33.1, 18.2, 7.7 | 12.6, 10.2, 6.5 | 583/(0.49, 0.46) |
|  | 20 | 3.3, 5.0, 7.4 | 6910 | 34.8, 30.5, 23.6 | 33.1, 19.6, 10.1 | 14.0, 12.2, 9.4 | 584/(0.50, 0.45) |
|  | 30 | 3.3, 5.9, 9.8 | 5921 | 31.2, 29.4, 21.9 | 31.7, 18.2, 9.0 | 12.2, 10.7, 8.3 | 584/(0.50, 0.45) |
|  | 40 | 3.0, 5.7, 9.7 | 5727 | 28.7, 23.0, 19.0 | 28.9, 17.6, 8.2 | 11.3, 9.7, 7.4 | 586/(0.52, 0.44) |
|  | 50 | 3.0, 5.4, 9.0 | 5307 | 28.3, 24.0, 19.4 | 28.0, 17.7, 7.9 | 11.1, 8.8, 7.0 | 588/(0.52, 0.44) |

[a] Operation voltages for turn on, and at 100 and 1000 cd m^-2^; [b] the maximum luminance; [c] Efficiencies at the maximum, 100 and 1000 cd m^-2^; [d] EL peak wavelengths and CIE coordinates at 1000 cd m^-2^

### Table S11. Electroluminescent performance of OLEDs with CBP as host material.

| EML | *x* (wt%) | *V*^[a]^ (V) | *L*_max_^[b]^  (cd m^-2^) | *η*^[c]^ | | | *λ*_EL_ (nm)/  CIE (x, y)^[d]^ |
| --- | --- | --- | --- | --- | --- | --- | --- |
|  |  |  |  | *η*_CE_ (cd A^-1^) | *η*_PE_ (lm W^-1^) | *η*_EQE_ (%) |  |
| **CBP:*x*% TTPPCuI** | 3 | 3.3, 4.4, 6.4 | 8458 | 33.6, 29.8, 18.5 | 32.0, 21.7, 9.6 | 11.6, 10.6, 6.6 | 572/(0.45, 0.51) |
|  | 5 | 3.2, 4.4, 6.4 | 9110 | 33.6, 27.3, 19.0 | 33.0, 19.9, 9.2 | 12.0, 9.8, 6.8 | 572/(0.45, 0.51) |
|  | 7 | 3.1, 4.2, 6.0 | 10810 | 35.4, 29.6, 21.0 | 35.1, 23.4, 11.0 | 13.3, 8.7, 6.0 | 573/(0.45, 0.51) |
|  | 10 | 3.0, 4.3, 6.1 | 11140 | 36.0, 29.0, 20.3 | 35.5, 21.5, 10.1 | 14.2, 10.6, 7.4 | 574/(0.47, 0.51) |
|  | 20 | 2.9, 4.0, 5.8 | 11000 | 33.0, 30.7, 22.7 | 31.7, 24.4, 12.5 | 13.2, 11.3, 8.4 | 575/(0.47, 0.51) |
|  | 30 | 2.8, 3.9, 5.6 | 9920 | 31.1, 29.1, 21.9 | 30.9, 24.4, 12.9 | 12.1, 11.2, 8.3 | 576/(0.47, 0.51) |
|  | 40 | 2.7, 3.7, 5.4 | 9801 | 30.2, 29.0, 22.7 | 29.7, 24.7, 13.2 | 11.3, 10.2, 8.7 | 577/(0.48, 0.49) |
| **CBP:*x*% FTTPPCuI** | 5 | 3.1, 4.4, 6.5 | 8766 | 34.9, 31.3, 21.1 | 34.3, 22.3, 10.1 | 12.1, 11.0, 7.4 | 572/(0.45, 0.51) |
|  | 10 | 3.1, 4.2, 6.1 | 11150 | 35.9, 30.3, 22.6 | 34.4, 23.6, 11.6 | 13.0, 11.2, 8.1 | 573/(0.45, 0.51) |
|  | 15 | 3.0, 4.3, 6.3 | 11210 | 41.9, 32.1, 22.6 | 37.4, 22.9, 11.0 | 14.4, 11.3, 8.1 | 574/(0.47, 0.51) |
|  | 20 | 3.0, 4.3, 6.4 | 11350 | 44.9, 33.3, 23.3 | 40.9, 24.5, 11.1 | 16.2, 12.0, 8.2 | 576/(0.47, 0.51) |
|  | 30 | 2.9, 4.0, 6.0 | 9110 | 40.6, 31.9, 24.2 | 39.4, 25.5, 12.7 | 15.0, 12.0, 8.8 | 582/(0.49, 0.47) |
|  | 40 | 2.8, 4.0, 6.0 | 9060 | 38.9, 31.9, 25.1 | 37.4, 26.7, 13.8 | 14.1, 12.0, 9.4 | 584/(0.50, 0.45) |
|  | 50 | 2.7, 3.8, 5.7 | 8850 | 37.2, 31.0, 22.9 | 35.9, 25.4, 12.6 | 13.5, 11.7, 8.6 | 586/(0.52, 0.44) |

| EML | *x* (wt%) | *V*^[a]^ (V) | *L*_max_^[b]^  (cd m^-2^) | *η*^[c]^ | | | *λ*_EL_ (nm) /  CIE (x, y)^[d]^ |
| --- | --- | --- | --- | --- | --- | --- | --- |
|  |  |  |  | *η*_CE_ (cd A^-1^) | *η*_PE_ (lm W^-1^) | *η*_EQE_ (%) |  |
| **CBP:*x*% 4FTTPPCuI** | 5 | 2.9, 5.0, 8.1 | 6069 | 9.4, 9.4, 7.4 | 6.0, 5.7, 3.0 | 4.2, 4.1, 3.3 | 596/(0.57, 0.36) |
|  | 10 | 2.9, 5.0, 8.3 | 4945 | 11.2, 10.3, 7.0 | 10.3, 6.6, 2.7 | 5.0, 4.6, 3.2 | 596/(0.57, 0.36) |
|  | 15 | 2.8, 4.7, 7.8 | 4283 | 12.9, 12.9, 9.2 | 10.1, 8.2, 3.9 | 5.5, 5.5, 3.9 | 600/(0.58, 0.36) |
|  | 20 | 2.8, 4.5, 7.8 | 4688 | 19.7, 13.2, 8.4 | 22.1, 9.3, 3.8 | 9.0, 6.0, 3.6 | 603/(0.58, 0.35) |
|  | 30 | 2.8, 4.9, 8.3 | 3804 | 18.5, 13.0, 8.0 | 18.3, 8.2, 3.1 | 8.0, 5.6, 3.5 | 603/(0.58, 0.35) |
|  | 40 | 2.8, 4.8, 8.2 | 4280 | 18.0, 14.6, 8.8 | 18.0, 9.4, 3.3 | 7.8, 7.0, 4.2 | 604/(0.59, 0.35) |
|  | 50 | 2.8, 5.2, 8.7 | 3143 | 15.5, 13.3, 8.4 | 15.0, 8.0, 3.0 | 7.2, 6.2, 4.0 | 604/(0.59, 0.35) |
| **CBP:*x*% 5FTTPPCuI** | 5 | 3.3, 5.3, 8.5 | 4498 | 9.5, 8.9, 7.3 | 9.1, 5.9, 2.8 | 5.2, 5.0, 4.0 | 588/(0.52, 0.44) |
|  | 10 | 3.2, 5.2, 8.2 | 4509 | 10.5, 9.9, 8.2 | 9.8, 6.7, 3.2 | 5.4, 5.3, 4.3 | 590/(0.53, 0.42) |
|  | 15 | 3.1, 5.2, 8.5 | 4939 | 10.6, 9.0, 6.9 | 9.9, 6.2, 2.7 | 5.9, 5.4, 4.1 | 592/(0.55, 0.40) |
|  | 20 | 3.0, 5.0, 8.4 | 5073 | 15.4, 13.4, 7.8 | 16.2, 8.7,3.0 | 7.8, 6.8, 4.4 | 596/(0.57, 0.36) |
|  | 30 | 3.0, 5.6, 9.1 | 4040 | 14.0, 12.1, 7.8 | 13.2, 7.0, 2.9 | 6.8, 5.7, 3.8 | 596/(0.57, 0.36) |
|  | 40 | 2.9, 5.6, 9.5 | 3727 | 12.7, 11.2, 7.1 | 11.9, 6.4, 2.2 | 6.3, 5.2, 3.4 | 598/(0.57, 0.36) |
|  | 50 | 2.8, 5.1, 9.0 | 2804 | 12.1, 10.4, 6.3 | 9.9, 5.6, 2.2 | 6.0, 4.7, 2.8 | 600/(0.58, 0.36) |

[a] Operation voltages for turn on, and at 100 and 1000 cd m^-2^; [b] the maximum luminance; [c] Efficiencies at the maximum, 100 and 1000 cd m^-2^; [d] EL peak wavelengths and CIE coordinates at 1000 cd m^-2^

### Table S12. Electroluminescent performance of representative Cu(I) complexes.

| Cu(I) complexes | Device structure | *V*^[a]^  (V) | *L*_max_^[b]^  (cd m^-2^) | *η*^[c]^ | | | *λ*_EL_ (nm)/  CIE (x, y)^[d]^ | Ref. |
| --- | --- | --- | --- | --- | --- | --- | --- | --- |
|  |  |  |  | *η*_CE_ (cd A^-1^) | *η*_PE_ (lm W^-1^) | *η*_EQE_ (%) |  |  |
|  | ITO\|PEDOT\|TCCz: 15% **Cu[NN1][PP1]** (80 nm)\|TPBi (40 nm)\|LiF (1 nm)\|Al | 9 | 930 | 6.4/0.9/- | -/-/- | 4.5/-/- | 630  (0.61, 0.39) | [9] |
|  | ITO\|MoO_3_ (1 nm)\|CBP (35 nm)\|5% **CuI:[TCIQ]** (20 nm)\|TPBi (65 nm)\|LiF (1 nm)\|Al | 3.2 | 7100 | 10.4/-/- | 10.2/-/- | 4.1/-/- | ~580  (0.49, 0.49) | [10] |
|  | ITO\|MoO_3_ (1 nm)\|HTL (35 nm)\|5% **CuI[CPzPC]** (20 nm)\|TPBi (65 nm)\|LiF (1 nm)\|Al (150 nm) | 3.6 | 8619 | 15.9/15.0/- | 14.0/10.1/- | 6.6/6.3/- | 590 | [11] |
|  | ITO\|PEDOT:PSS\|PYD2: 5% **Cu[NN2][PP2]** (60 nm)\|DPEPO (5 nm)\|TPBi (40 nm)\|LiF (1.2 nm)\|Al | 4 | 5580 | 43.3/-/- | 19.8/-/- | 15.6/-/- | 574  (0.48, 0.51) | [12] |
|  | (ITO)\|PEDOT: PSS\|PVK: **Cu[NN3][PP1**]\|TPBi (30nm)\|Ca (30nm)\|Al (150 nm) | 10.6 | 197 | -/-/- | -/-/- | 0.8/-/- | ~600  (0.42;0.46) | [13] |
|  | ITO\|MoO_3_ (6 nm)\|TAPC (50 nm)\|*m*CP: 10% **TTPPCuI** (20 nm)\|TPBi (50 nm)\|LiF (1 nm)\|Al (100 nm) | 3.5 | >10000 | 40.8/-/- | 35.9/-/- | 16.3/-/- | ~575  (0.48, 0.49) | [14] |
|  | ITO\|MoO_3_ (6 nm)\|TAPC (50 nm)\|*m*CP: 10% **TTPPCuBr** (20 nm)\|TPBi (50 nm)\|LiF (1 nm)\|Al (100 nm) | 3.5 | ~9000 | 32.7/-/- | 28.8/-/- | 12.4/-/- | ~575  (0.47, 0.50) | [14] |
|  | ITO\|MoO_3_ (6 nm)\|TAPC (50 nm)\|*m*CP: 10% **TTPPCuCl** (20 nm)\|TPBi (50 nm)\|LiF (1 nm)\|Al (100 nm) | 3.5 | ~10000 | 24.0/-/- | 21.4/-/- | 9.6/-/- | ~575  (0.47, 0.50) | [14] |
|  | ITO\|PEDOT: PSS\|PYD2: 12% **Cu[dppnc][Y]** (60 nm) \|DPEPO (10 nm)\|TPBi (40 nm)\|LiF (1.2 nm)\|Al (100 nm) | - | 17600 | 35.3/- 31.9/ | 10.0/-/8.0 | 14.3/-/13.1 | 582  (0.51, 0.48) | [15] |
|  | ITO\|PEDOT: PSS\|PYD2: 8% **Cu[dppnc][R1]** (60 nm) \|DPEPO (10 nm)\|TPBi (40 nm)\|LiF (1.2 nm)\|Al (100 nm) | - | 5280 | 12.7/-/ 6.7 | 8.2/-/ 3.9 | 9.0/-/4.6 | 622  (0.57, 0.38) | [15] |
|  | ITO\|PEDOT: PSS\|CBP: 4% **Cu[dppnc][R2]** (60 nm) \|DPEPO (10 nm)\|TPBi (40 nm)\|LiF (1.2 nm)\|Al (100 nm) | - | 4630 | 11.3/-/3.9 | 4.1/-/1.1 | 10.2/-/3.5 | 631  (0.61, 0.38) | [15] |
|  | ITO\|PEDOT: PSS (35 nm)\|TCTA: 10% **Cu[MD]** (50 nm)\|TPBI (40 nm)\|LiF (1 nm)\|Al (100 nm) | 4.3 | 2466 | 3.2/-/- | 1.5/-/- | 2.2/-/- | 637  (0.50, 0.45) | [16] |
|  | ITO\|PEDOT: PSS (40 nm)\|CBP: 20 % **CuI[dpna]** (30 nm)\|TPBi (40 nm)\|LiF (1 nm)\|Al (100 nm) | ~7.5 | ~100 | 0.04/-/- | 0.01/-/- | 0.1/-/- | ~650  (0.62, 0.38) | [17] |
|  | ITO\|NPB (20 nm)\|CBP: 10% **CuI[NP][TPP]** (50 nm)\|BCP (10 nm)\|Alq_3_ (30 nm)\|LiF (1 nm)\|Al (100 nm) | 3.5 | 1806 | 10.0/-/- | -/-/- | ~3.4%/-/- | 613  (0.52, 0.46) | [18] |
|  | ITO)\|MoO_3_ (1 nm)\|CBP (35 nm)\|**CuI:2CzPQ** (20 nm)\|TPBi (65 nm)\|LiF (1 nm)\|Al (100 nm) | 3.7 | 7521 | 14.1/-/- | 11.7/-/- | 6.7/-/- | 600 | [19] |
|  | ITO\|PEDOT:PSS (40 nm)\|poly-TPD (50 nm)\|**Cu[PP]** (30 nm)\|TPBi (40 nm)\|LiF (1 nm)\|Al (100 nm) | - | ~10 | -/-/- | -/-/- | 0.3%/-/- | ~580  (0.44, 0.47) | [20] |
|  | ITO\|PEDOT:PSS (40 nm)\|TCTA (20 nm)\|**CuCl[SBF]** (20 nm)\|TPBi (50 nm)\|Liq (1 nm)\|Al (120 nm) | 5.1 | 6799 | 24.3/-/- | 12.9/-/- | 9.5/-/- | ~590 | [21] |
|  | ITO\|NPB (30 nm)\|CBP: 10% **CuI[PNP]** (50 nm)\|BCP (10 nm)\|Alq_3_ (30 nm)\|LiF (1 nm)\|Al (100 nm) | 4 | 1970 | 8.2/-/- | -/-/- | 2.7/-/- | 645  (0.65, 0.35) | [22] |
|  | ITO\|HAT-CN (10 nm)\|NPB (10 nm)\|TCTA (20 nm)\|**CuI[*β*-aza-SBF]** (20 nm)\|Cz35PyDCb (5 nm)\|TmPyPB (50 nm)\|Liq (1  nm)\|Al (100 nm) | 4.5 | 6740 | 7.6/5.9/- | 11.3/12.8/- | 16.5/5.4/- | 588 | [23] |
|  | ITO\|PEDOt: PSS (25 nm)\|Poly-TPD (30 nm)\|TcTA: **Cu[OXD]C2**\|Al (100 nm) | 8.0 | 396 | 0.05/-/- | -/-/- | 0.02/-/- | 578  (0.45, 0.48) | [24] |
|  | ITO\|PEDOT:PSS (50 nm)\|OTPD (4 nm)\|PYD2: **Cu[NN4][PP1]** (60 nm)\|DPEPO (10 nm)\|TPBi (40 nm)\|LiF (1.2 nm)\|Al (100 nm) | - | ~1000 | -/-/- | -/-/- | 8.0/-/- | ~580  (0.51, 0.49) | [25] |
|  | ITO\|MoO_3_ (2 nm)\|TAPC (80 nm)\|*m*CP (10 nm) CBP: TPBi: 1.0% **Cu[MAC1]** (20 nm)\|TPBi (10 nm)\|3TPYMB (60 nm)\|LiF (1 nm)\|Al (140 nm) | 2.9 | 16900 | 30.2/30.2/27.5 | 30.1/23.6/15.0 | 20.7/20.6/18.8 | 622  (0.58, 0.42) | [26] |
|  | ITO\|MoO_3_ (2 nm)\|TAPC (80 nm)\|*m*CP (10 nm) CBP: TPBi: 1.5% **Cu[MAC2]** (20 nm)\|TPBi (10 nm)\|3TPYMB (60 nm)\|LiF (1 nm)\|Al (140 nm) | 3.1 | 17706 | 31.2/31.1/29.6 | 28.9/22.3/15.1 | 21.1/21.0/20.1 | 628  (0.58, 0.42) | [26] |
|  | ITO\|MoO_3_ (2 nm)\|TAPC (80 nm)\|*m*CP (10 nm) CBP: TPBi: 3.0% **Cu[MAC3]** (20 nm)\|TPBi (10 nm)\|3TPYMB (60 nm)\|LiF (1 nm)\|Al (140 nm) | 3.1 | 9230 | 19.9/19.8/17.6 | 18.4/13.3/8.0 | 17.6/17.5/15.8 | 629  (0.57, 0.43) | [26] |
|  | ITO\|PEDOT: PSS\|CBP: **Cu[iTMCs]**\|Al | - | 152.8 | -/-/- | 0.1/-/- | 0.1/-/- | 626 (0.58/0.41) | [27] |
|  | ITO\|HAT-CN (5 nm)\|TAPC (50 nm)\|TCTA (10 nm)\|TCTA:TPBi: **Cu[CMA1]** (20 nm)\|HBL (10 nm)\|TPBi (40 nm)\|LiF (1 nm)\|Al (100 nm) | ~2.5 | 222200 | 44.7/-/- | 46.2/-/- | 18.7/-/18.3 | 582  (0.51, 0.48) | [28] |
|  | ITO\|HAT-CN (5 nm)\|TAPC (50 nm)\|TCTA (10 nm)\|TCTA:TPBi: **Cu[CMA3]** (20 nm)\|HBL (10 nm)\|TPBi (40 nm)\|LiF (1 nm)\|Al (100 nm) | ~2.5 | 155000 | 22.1/-/- | 22.0/-/- | 14.4/-/13.8 | 619  (0.58, 0.42) | [28] |
|  | ITO\|EDOT:PSS (40 nm)\|*m*CP: **CuCl[X-3]** (25 nm)\|TPBi (30 nm)\|LiF (1 nm)\|Al (100 nm) | 7.3 | 1030 | -/-/- | -/-/- | 2.3/-/- | 572  (0.44, 0.50) | [29] |
|  | ITO\|HAT-CN (5 nm)\|TAPC (50 nm)\|TCTA (10 nm)\|TCTA : DPEPO: 10% **CuI-4** (25 nm)\|DPEPO (10 nm)\|TPBi (40 nm)\|LiF (1 nm)\|Al (100 nm) | 4.0 | 845 | 33.44/32.41/- | 21.63/17.85/- | 14.6/14.1/- | 584  (0.46, 0.50) | [30] |
|  | ITO\|PEDOT: PSS (40 nm)\|*m*CP: 10% **QCzTTPPCuI** (20 nm)\|DBDPO (35 nm)\|LiF (1 nm)\|Al (100 nm) | 4.7 | 3075 | 44.9/44.6/32.0 | 33.5/23.9/12.4 | 17.5/17.3/14.3 | 570/(0.46, 0.51) | [31] |
|  | ITO\|PEDOT: PSS (40 nm)\|*m*CP: 10% **QtBCzTTPPCuI** (20 nm)\|DBDPO (35 nm)\|LiF (1 nm)\|Al (100 nm) | 4.7 | 5954 | 76.8/76.1/68.7 | 56.0/42.3/28.4 | 29.1/28.7/25.9 | 570/(0.45, 0.52) | [31] |
|  | ITO\|HATCN (5 nm)\|TAPC (30 nm)\|TCTA (15 nm)\|mCBP (10 nm)\|DMIC-TRZ: 1% **CuI[DBC]** (45 nm)\|POT2T (20 nm)\|ANT-BIZ (30 nm)\|Liq (2 nm)\|Al (100 nm) | 2.8 | 232325 | 78.6/-/- | 76.8/-/- | 29.5/-/- | 576 (0.48, 0.51) | [32] |
|  | ITO\|MoO_3_ (6 nm)\|TAPC (50 nm)\|*m*CP: 10% **FTTPPCuI** (20 nm)\|TPBi (50 nm)\|LiF (1 nm)\|Al (100 nm) | 3.2 | 16870 | 64.0/55.5/44.2 | 62.8/42.0/24.2 | 22.4/19.7/15.4 | 574/(0.47, 0.51) | This work |
|  | ITO\|MoO_3_ (6 nm)\|TAPC (50 nm)\|CBP: 10% **4FTTPPCuI** (20 nm)\|TPBi (50 nm)\|LiF (1 nm)\|Al (100 nm) | 2.8 | 4688 | 19.7/13.2/8.4 | 22.1/9.3/3.8 | 9.0/6.0/3.6 | 603  (0.58, 0.35) | This work |
|  | ITO\|MoO_3_ (6 nm)\|TAPC (50 nm)\|*m*CP: 10% **5FTTPPCuI** (20 nm)\|TPBi (50 nm)\|LiF (1 nm)\|Al (100 nm) | 3.3 | 6910 | 34.8/30.5/23.6 | 33.1/19.6/10.1 | 14.0/12.2/9.4 | 584  (0.50, 0.45) | This work |

[a] Operation voltages for turn on, and at 100 and 1000 cd m^-2^; [b] the maximum luminance; [c] Efficiencies at the maximum, 100 and 1000 cd m^-2^; [d] EL peak wavelengths and CIE coordinates at 1000 cd m^-2^

### References

1. Becke AD. Densityfunctional thermochemistry. III. The role of exact exchange. *J. Chem. Phys.* 1993;**98**(7):5648-5652. <http://doi.org/10.1063/1.464913>.
2. Lee C, Yang WT, Parr RG. Development of the Colle-Salvetti correlation-energy formula into a functional of the electron density. *Phys. Rev. B.* 1988;**37**(2):785-789. <https://doi.org/10.1103/PhysRevB.37.785>.
3. Hay PJ, Wadt WR. Ab initio effective core potentials for molecular calculations. Potentials for the transition metal atoms Sc to Hg. *J. Chem. Phys.* 1985;**82**(1):270-283. <http://doi.org/10.1063/1.448799>.
4. Martin RL. Natural transition orbitals. *J. Chem. Phys.* 2003;**118**(11):4775-4777. <https://doi.org/10.1063/1.1558471>.
5. Frisch MJ, Trucks GW, Schlegel HB, Scuseria GE, Robb MA, Cheeseman JR, Scalmani G, Barone V, Mennucci B, Petersson GA, et al. Gaussian 09 (Gaussian). 2009;<https://gaussian.com/>.
6. Chuen CH, Tao YT, Wu FI, Shu CF. White organic light-emitting diodes based on 2,7-bis(2,2-diphenylvinyl)-9,9′-spirobifluorene: Improvement in operational lifetime. *Appl. Phys. Lett.* 2004;**85**(20):4609-4611. <https://doi.org/10.1063/1.1824178>.
7. Yang SH, Huang TL. High fluorescence efficiency of dual-wavelength white OLED with NPB emission and triplet annihilation. *Opt. Mater.* 2021;**111**:110725. <https://doi.org/10.1016/j.optmat.2020.110725>.
8. Hofbeck T, Monkowius U, Yersin H. Highly Efficient Luminescence of Cu(I) Compounds: Thermally Activated Delayed Fluorescence Combined with Short-Lived Phosphorescence. *J. Am. Chem. Soc.* 2015;**137**(1):399-404. <https://doi.org/10.1021/ja5109672>.
9. Zhang QS, Ding JQ, Cheng YX, Wang LX, Xie ZY, Jing XB, Wang FS. Novel Heteroleptic CuI Complexes with Tunable Emission Color for Efficient Phosphorescent Light-Emitting Diodes. *Adv. Funct. Mater.* 2007;**17**(15):2983-2990. <https://doi.org/10.1002/adfm.200601053>.
10. Liu XC, Zhang T, Ni TC, Jiang N, Liu ZW, Bian ZQ, Lu ZH, Huang CH. Co-deposited Cu(I) Complex for Tri-layered Yellow and White Organic Light-Emitting Diodes. *Adv. Funct. Mater.* 2014;**24**(34):5385-5392. <https://doi.org/10.1002/adfm.201400685>.
11. Wei F, Qiu J, Liu XC, Wang JQ, Wei HB, Wang ZB, Liu ZW, Bian ZQ, Lu ZH, Zhao YL, et al. Efficient orange-red phosphorescent organic light-emitting diodes using an in situ synthesized copper(I) complex as the emitter. *J. Mater. Chem. C* 2014;**2**(31):6333-6341. <https://doi.org/10.1039/c4tc00410h>.
12. Cheng G, So GKM, To WP, Chen Y, Kwok CC, Ma CS, Guan XG, Chang XY, Kwok WM, Che CM. Luminescent zinc(II) and copper(I) complexes for high-performance solution-processed monochromic and white organic light-emitting devices. *Chem. Sci.* 2015;**6**(8):4623-4635. <https://doi.org/10.1039/c4sc03161j>.
13. Bizzarri C, Fléchon C, Fenwick O, Cacialli F, Polo F, Gálvez-López MD, Yang C-H, Scintilla S, Sun Y, Fröhlich R, et al. Luminescent Neutral Cu(I) Complexes: Synthesis, Characterization and Application in Solution-Processed OLED. *ECS J. Solid State Sci. Technol.* 2016;**5**(6):R83. <https://doi.org/10.1149/2.0021606jss>.
14. Zhang J, Duan CB, Han CM, Yang H, Wei Y, Xu H. Balanced Dual Emissions from Tridentate Phosphine-Coordinate Copper(I) Complexes toward Highly Efficient Yellow OLEDs. *Adv. Mater.* 2016;**28**(28):5975-5979. <https://doi.org/10.1002/adma.201600487>.
15. So GKM, Cheng G, Wang J, Chang XY, Kwok CC, Zhang HX, Che CM. Efficient Color-Tunable Copper(I) Complexes and Their Applications in Solution-Processed Organic Light-Emitting Diodes. *Chem. Asian J.* 2017;**12**(13):1490-1498. <https://doi.org/10.1002/asia.201700081>.
16. Yang XL, Yan Xg, Guo HR, Liu BA, Zhao J, Zhou GJ, Wu Y, Wu ZX, Wong WY. Charged dinuclear Cu(I) complexes for solution-processed single-emitter warm white organic light-emitting devices. *Dyes Pigm.* 2017;**143**:151-164. <https://doi.org/10.1016/j.dyepig.2017.04.036>.
17. Liu LP, Li Q, Xiang SP, Liu L, Zhong XX, Liang C, Li GH, Hayat T, Alharbi NS, Li FB, et al. Near-saturated red emitters: four-coordinate copper(I) halide complexes containing 8-(diphenylphosphino)quinoline and 1-(diphenylphosphino)naphthalene ligands. *Dalton Trans.* 2018;**47**(28):9294-9302. <https://doi.org/10.1039/d1dt03759e>.
18. Jiao BJ, Wang JY, Huang J, Cao MM, Liu CM, Yin GJ, Zhu YY, Zhang B, Du CX. Design and synthesis of stable cuprous complexes bearing P^∧^N-type ligands for vapor-deposited organic light-emitting device. *Org. Electron.* 2019;**64**:158-165. <https://doi.org/10.1016/j.orgel.2018.10.011>.
19. Li JY, Li XY, Tan Y, Yu X, Yuan FL, Liu ZW, Bian ZQ, Jin QH, Lu ZH, Huang CH. Construction of High-Quality Cu(I) Complex-Based WOLEDs with Dual Emissive Layers Achieved by an "On-and-Off" Deposition Strategy. *Adv. Optical Mater.* 2019;**7**(10):1801612. <https://doi.org/10.1002/adom.201801612>.
20. Liu LP, Zhang R, Liu L, Zhong XX, Li FB, Wang L, Wong WY, Li GH, Cong HJ, Alharbi NS, et al. A new strategy to synthesize three-coordinate mononuclear copper(I) halide complexes containing a bulky terphenyl bidentate phosphine ligand and their luminescent properties. *New J. Chem.* 2019;**43**(8):3390-3399. <https://doi.org/10.1039/c8nj05605f>.
21. Yang LQ, Xu XX, Zhang PP, Chen MY, Chen G, Zheng YQ, Wei B, Zhang JH. Photophysical properties and stability of binuclear emissive Copper(I) complexes Co-deposited with CuX (X=Cl, Br, I) and aza-9,9′-spirobifluorenes. *Dyes Pigm.* 2019;**161**:296-302. <https://doi.org/10.1016/j.dyepig.2018.09.024>.
22. Song YL, Jiao BJ, Liu CM, Peng XL, Wang MM, Yang Y, Zhang B, Du CX. Synthesis, structures and luminescent properties of red emissive neutral copper(I) complexes with bisphosphino-substituted benzimidazole. *Inorg. Chem. Commun.* 2020;**112**:107689. <https://doi.org/10.1016/j.inoche.2019.107689>.
23. Guo JF, Zhang Z, Wu P, Zhu J, Dou D, Liao ZC, Xia RR, Wang K, Wang ZX. Co-deposited copper(I) complexes integrating phosphorescence and TADF properties for highly efficient OLEDs. *J. Lumin.* 2021;**239**:118354. <https://doi.org/10.1016/j.jlumin.2021.118354>.
24. Salla CAM, Farias G, Pereira A, Cremona M, Quirino WG, Legnani C, de Souza B, Bechtold IH. Enhanced Performance of All-Solution Processed Multilayer OLEDs by Photoluminescence Lifetime Reduction of Cu(I) Complex Emitters Containing Chalcogenolate-Diimine Ligands. *Eur. J. Inorg. Chem.* 2021;**2021**(33):3412-3418. <https://doi.org/10.1002/ejic.202100438>.
25. Teng T, Xiong JF, Cheng G, Zhou CJ, Lv XL, Li K. Solution-Processed OLEDs Based on Thermally Activated Delayed Fluorescence Copper(I) Complexes with Intraligand Charge-Transfer Excited State. *Molecules* 2021;**26**(4):1125. <https://doi.org/10.3390/molecules26041125>.
26. Ying A, Huang YH, Lu CH, Chen ZX, Lee WK, Zeng X, Chen TH, Cao XS, Wu CC, Gong SL, et al. High-Efficiency Red Electroluminescence Based on a Carbene–Cu(I)–Acridine Complex. *ACS Appl. Mater. Interfaces* 2021;**13**(11):13478-13486. <https://doi.org/10.1021/acsami.0c22109>.
27. Fresta E, Mahoro GU, Cavinato LM, Lohier JF, Renaud J, Gaillard S, Costa RD. Novel Red-Emitting Copper(I) Complexes with Pyrazine and Pyrimidinyl Ancillary Ligands for White Light-Emitting Electrochemical Cells. *Adv. Optical Mater.* 2022;**10**(3):2101999. <https://doi.org/10.1002/adom.202101999>.
28. Tang R, Xu S, Lam TL, Cheng G, Du L, Wan Q, Yang J, Hung F, Low KH, Phillips DL, et al. Highly Robust CuI‐TADF Emitters for Vacuum‐Deposited OLEDs with Luminance up to 222 200 cd m^-2^ and Device Lifetimes (LT90) up to 1300 hours at an Initial Luminance of 1000 cd m^-2^. *Angew. Chem. Int. Ed.* 2022;**61**:e202203982. <https://doi.org/10.1002/anie.202203982>.
29. Wei Q, Gong FF, Zhang R, Liu L, Zhong XX, Wang L, Li FB, Wong WY, Qin HM. Mononuclear Cu(I) halide complexes with two thiophenyl rings triphosphine: Structure and photophysical properties. *J. Lumin.* 2022;**250**:119098. <https://doi.org/10.1016/j.jlumin.2022.119098>.
30. Liu YL, Zhu RQ, Liu L, Zhong XX, Li FB, Zhou GJ, Qin HM. High-performance TADF-OLEDs utilizing copper(I) halide complexes containing unsymmetrically substituted thiophenyl triphosphine ligands. *Inorg. Chem. Front.* 2025;**12**(3):1139-1155. <https://doi.org/10.1039/d4qi02415j>.
31. Niu JX, Gao FF, Wang Y, Lu W, Zhang J, He JX, Lou XJ, Ma YX, Duan CB, Han CM, et al. Equalized dual emissions from copper complexes via multichannel balanced intersystem crossing: towards 100% quantum efficiencies. *Angew. Chem. Int. Ed.* 2025;**64**(28):e202508667. <https://doi.org/10.1002/anie.202508667>.
32. Zhang QZ, Li NQ, Wan XT, Song XF, Zhang Y, Liu H, Miao JS, Zou Y, Yang CL, Li K. Harnessing of Cooperative Cu⋅⋅⋅H Interactions for Luminescent Low-Coordinate Copper(I) Complexes towards Stable OLEDs. *Angew. Chem. Int. Ed.* 2025;**64**(7):e202419290. <https://doi.org/10.1002/anie.202419290>.
